# CRISPR RNA architecture steers Cas9 catalysis and fidelity via a guide repeat clasp motif

**DOI:** 10.1093/nar/gkag710

**Published:** 2026-08-14

**Authors:** Ramadevi Chilamkurthy, Sruthi Sudhakar, Adrian A Pater, Michael S Bosmeny, Francis Stabile, Adam Katolik, S Harikrishna, Fatima El Azzouzi, Rolf Turk, Masad J Damha, Sabrina Leslie, Sergey Korolev, P I Pradeepkumar, Keith T Gagnon

**Affiliations:** Department of Biochemistry, Wake Forest University School of Medicine, Winston-Salem, NC, United States; Department of Chemistry, Indian Institute of Technology Bombay, Powai, Mumbai, India; Department of Biochemistry, Wake Forest University School of Medicine, Winston-Salem, NC, United States; Department of Biochemistry, Wake Forest University School of Medicine, Winston-Salem, NC, United States; Department of Physics, McGill University, Montreal, Quebec, Canada; Department of Chemistry, McGill University, Montreal, Quebec, Canada; Department of Chemistry, Indian Institute of Technology Bombay, Powai, Mumbai, India; Department of Biochemistry, Wake Forest University School of Medicine, Winston-Salem, NC, United States; Integrated DNA Technologies, Coralville, IA, United States; Department of Chemistry, McGill University, Montreal, Quebec, Canada; Department of Physics, McGill University, Montreal, Quebec, Canada; Michael Smith Laboratory and Department of Physics and Astronomy, University of British Columbia, Vancouver, British Columbia, Canada; Department of Biochemistry and Molecular Biology, School of Medicine, Saint Louis University, St. Louis, MO, United States; Department of Chemistry, Indian Institute of Technology Bombay, Powai, Mumbai, India; Department of Biochemistry, Wake Forest University School of Medicine, Winston-Salem, NC, United States

## Abstract

A widely adopted modification of CRISPR–Cas9 is fusion of the naturally occurring two-component dual guide RNA (dgRNA) to create an artificial single guide RNA (sgRNA). Here we find that these guide architectures induce differential catalysis, gene editing, and specificity. Spacer sequence and RNA structural features could not predict guide architecture editing preference across 255 endogenous targets. We used cryo-EM and molecular dynamics to identify a new Cas9 structural motif, the guide repeat clasp (GRC), that checks guide RNA repeat structure and coordinates with R-loop sensing checkpoint mechanisms to help license cleavage. Limited mutagenesis of GRC residues significantly altered Cas9 editing and specificity, supporting a key role in catalysis. To further understand the role of the GRC and guide RNA repeat dynamics, we created guide repeat-truncated sgRNAs, or grtRNAs, which conferred some dgRNA properties onto sgRNA, including generally lower off-target editing for targets with PAM-proximal mismatches. dgRNAs and grtRNAs could be combined with a new high-fidelity Cas9 variant called ZiFY, rationally designed to reduce editing of targets with PAM-distal mismatches, to generate broader mismatched target discrimination. These results uncover a previously unknown mechanism that steers Cas9 catalysis and demonstrate the potential to improve Cas9 fidelity by modulating guide repeat interactions.

## Graphical Abstract

**Graphical Abstract:**
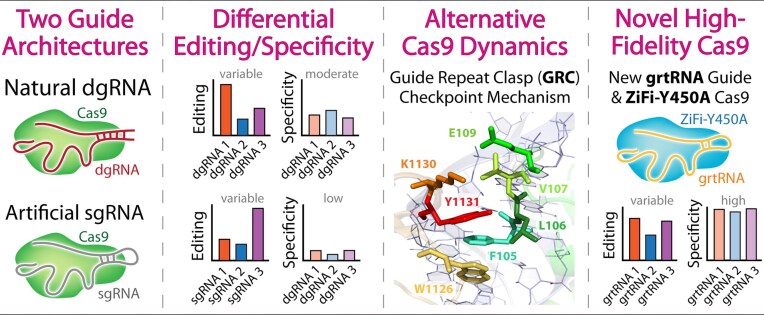

## Introduction

Cas9 from *Streptococcus pyogenes* (SpCas9) is a prototypical CRISPR-associated effector nuclease used for gene editing in biotechnology and therapeutics [1–14]. SpCas9 evolved to naturally use two distinct RNAs, CRISPR RNA (crRNA) and *trans*-activating crRNA (tracrRNA), to guide DNA cleavage [15–19]. An early modification was fusion of the dual guide RNA (dgRNA) system into an artificial single guide RNA (sgRNA) architecture [20], which simplified design and delivery. The use of sgRNA has become the conventional approach [18, 21–27]. Conversely, dgRNA has often been considered less efficient [18, 20, 28] despite successful use and commercialization [29–33]. Consequently, differences in the activity or mechanisms between these two guide RNA architectures have been largely overlooked and not investigated systematically [18, 20, 25, 34, 35].

Despite extensive characterization of SpCas9, no structural studies with dgRNA have been previously reported, and tools to predict the best SpCas9 guides for editing efficiency and specificity are trained with sgRNA data [23, 24, 36, 37]. However, some applications may benefit from dgRNAs [38]. For example, chemical modification of crRNA and tracrRNA is easier and less resource-intensive compared to longer sgRNA [30, 32, 39], which could enable certain CRISPR-based therapeutics [29, 40, 41]. Studying SpCas9 guided by natural dgRNA may also uncover new mechanisms of regulation or provide insights into molecular evolution or CRISPR biology [38].

In this study, we performed a systematic comparison of SpCas9 activity when guided by dgRNA or sgRNA. We found that dgRNAs often performed as well or better across multiple spacer sequences and usually induced lower off-target editing. Trends in spacer sequence and guide RNA structure predictions could not predict guide architecture editing preferences. By using model spacers, biochemical and cell-based assays, cryo-electron microscopy (cryo-EM), molecular dynamics (MD) simulations, and modification of SpCas9 and guide RNA, we identified a previously uncharacterized mechanism, which we refer to as the guide repeat clasp (GRC). The GRC senses SpCas9 interaction with the guide RNA repeat, the only structural difference between sgRNA and dgRNA. Our results support the coordination of the GRC with previously known checkpoint mechanisms involved in R-loop sensing, thereby explaining the codependence of spacer sequence and guide architecture in steering SpCas9 catalysis. Combining both guide architectures into a guide repeat-truncated sgRNA design, called grtRNA, created a smaller sgRNA that typically conferred dgRNA properties onto sgRNA, specifically a moderate reduction in off-target editing. We also found that dgRNA and grtRNA could be favorably paired with a new high-fidelity variant of SpCas9 that we created, referred to as ZiFY, to further enhance specificity. Thus, guide RNA interactions with SpCas9 GRC residues represent a new mechanism that can be explored, in combination with other known mechanisms, to create higher-fidelity enzymes.

## Materials and methods

### RNA synthesis

DNA oligonucleotides, crRNAs, tracrRNAs, and sgRNAs were chemically synthesized and HPLC-purified by Integrated DNA Technologies (IDT). Chemically modified tracrRNA was custom synthesized and HPLC-purified following our previously published protocols [42, 43]. All RNA and DNA oligonucleotide sequences can be found in Supplementary Table S1.

### Preparation of Cas9 enzymes

Plasmid encoding *Streptococcus pyogenes* Cas9 (SpCas9) with a C-terminal fusion of a nuclear localization signal (NLS) and a 6×-Histidine tag (pET-Cas9-NLS-6×His) [44] was obtained from Addgene (62933). A catalytically inactive “dead” Cas9 (dCas9) and other mutants were prepared by performing site-directed mutagenesis on this plasmid to generate H840A and D10A mutations (pET-dCas9-NLS-6×His). Site-directed mutagenesis was performed using an inverse PCR cloning strategy. Briefly, mutagenic primers containing the desired nucleotide substitutions and 15 bp of overlapping overhang sequence were used to amplify the entire plasmid with Q5 High-Fidelity DNA Polymerase (New England Biolabs) with a two-step PCR cycle and annealing temperature of 72°C. The Takara Bio primer design tool for mutagenesis (https://www.takarabio.com/learning-centers/cloning/primer-design-and-other-tools) was used for primer design. The resulting linear PCR products were then ligated into circular plasmids using the In-Fusion cloning system (Takara Bio) and transformed into chemically competent NEB Stable *E. coli* cells (New England Biolabs). Cas9 proteins were prepared similarly to previously described methods [45] with some alterations. Briefly, protein expression was induced in Rosetta (DE3) cells with 0.4 mM IPTG at 18°C for 16 h. Cell pellets were resuspended in chilled binding buffer (20 mM Tris–HCl, pH 7.5, 250 mM NaCl, 1 mM PMSF, 5 mM imidazole). Resuspended cells were lysed by either sonication or French press and the resulting lysate was clarified by centrifugation. His-Pur Cobalt-CMA resin (Thermo Scientific) was equilibrated with binding buffer, and the supernatant was added and rotated at 4°C for 1 h. The supernatant was washed sequentially with at least three bed volumes each of increasing concentrations of NaCl in wash buffer (Tris–HCl, pH 7.5, 0.25/0.5/0.75/1.0 M NaCl, 10 mM imidazole). Protein was eluted with elution buffer (Tris–HCl, pH 7.5, 250 mM NaCl, 130 mM imidazole). Purified Cas9 enzyme was concentrated and buffer-exchanged into gel filtration-running buffer (20 mM Tris–HCl, pH 7.5, 200 mM KCl, 0.5 mM DTT) using Vivaspin 15 centrifugal concentrators (Sartorius, 30K MWCO). Concentrated protein was injected into pre-equilibrated Superdex 200 column, and 1 ml fractions were collected. Fractions with Cas9 were concentrated, buffer exchanged into 2× storage buffer (40 mM Tris–HCl, pH 7.5, 400 mM KCl, 1 mM DTT), and an equal volume of glycerol was added to obtain final 50% glycerol in 1× storage buffer. Concentration was determined by UV absorbance at 280 nm using a calculated extinction coefficient (120 450 M^−1^ cm^−1^) [46] and Beer’s law.

### RNA secondary structure prediction and statistical analysis

Minimum free energy (MFE) secondary structures of guide RNA spacers were predicted in both the crRNA (dgRNA) and sgRNA scaffold contexts using RNAfold from the ViennaRNA package (v2.7.2) at 37°C with the default Turner 2004 nearest-neighbor parameters [47, 48]. Each 20 nt spacer was appended to the 5′ end of the crRNA repeat scaffold (40 nt construct) or the full sgRNA scaffold (96 nt construct) for the 255 reference spacers and the five EGFP spacers (E1 through E5). Structural features, including MFE, fraction of paired nucleotides, hairpin count, maximum nesting depth, GC content, total and 5′-end spacer pairing, and spacer pairing to the scaffold, stem-loop 1 (SL1), and repeat-antirepeat (RAR) units, were extracted from the predicted dot-bracket structures with custom Python scripts, and pairwise base-pair distances were computed with the ViennaRNA RNA.bp_distance() function.

A composite structural score was derived by multiple regression of z-scored structural features against the editing efficiency differential (Δ%EE = %EE dgRNA − %EE sgRNA). Sequence logos for spacers stratified by architecture preference were generated with WebLogo 3 [49]. Differences in structural features across preference groups were assessed by the Kruskal–Wallis test and associations between structural metrics and editing efficiency by Pearson correlation, with a significance threshold of α = 0.05.

Unless otherwise indicated, cell-based and biochemical comparisons used *n* = 3 or more experimental replicates and unpaired two-tailed t-tests, with significance thresholds reported in the figure legends (n.s., not significant; *P < .05; **P < .01; ***P < .005; ^****^P < .0001). Sample sizes were selected based on the expectation that three or more replicates are representative. Error is reported as standard error of the mean (SEM) or standard deviation (SD) as indicated in each figure legend.

### *In vitro* Cas9 cleavage activity assays

*In vitro* cleavage assays were performed as previously described [32, 50]. Two versions of EGFP target gene (EGFP, non-codon optimized; EGIP, codon optimized) were used to access the intrinsic enzyme activity of Cas9 loaded with different guide RNAs. Plasmid DNA containing the EGFP target gene (pSMART-EGFP-Cut1c) was linearized with either HindIII or KasI restriction enzymes and then purified by phenol-chloroform extraction and ethanol precipitation. A 1 kb fragment of the target EGIP gene was PCR-amplified using “pEGIP_vitro target” primers from pEGIP plasmid DNA (Addgene, 26777) and purified by phenol-chloroform extraction and ethanol precipitation.

Briefly, a master mix containing final concentrations of 0.75 µM Cas9, 0.25 µM tracrRNA, 0.3 µM crRNA, or 0.75 µM Cas9 and 0.25 µM sgRNA in a 1× cleavage buffer (20 mM Tris–HCl, pH 7.5, 100 mM KCl, 5% glycerol, 1 mM DTT, 0.5 mM EDTA, 2 mM MgCl_2_) supplemented with 0.1 mg/ml of purified yeast tRNA was incubated at 37°C for 10 min to allow the RNP to assemble. The concentration of tracrRNA or sgRNA was purposely set as the limiting component of the RNP complex and used to predict final RNP concentration. To each reaction tube containing 1 µl of target DNA (100 ng/µl), 39 µl of pre-assembled RNP was added at 37°C, and reactions were stopped at specified time points by addition of 2% LiClO_4_ in acetone and placed on ice. The samples were allowed to precipitate at −20°C for at least 1 h, pelleted by centrifugation, and washed with acetone. Air-dried pellets were resuspended in TE buffer or ddH_2_O and then treated with 10 μg of RNase A (Thermo Scientific) for 15 min, followed by 20 μg of proteinase K (Thermo Scientific) for 15 min at room temperature. Samples were mixed with 6× Purple Gel Loading Dye (New England Biolabs) and the cleavage products were resolved on 1% or 1.5% Tris-borate-EDTA (TBE) agarose gels. Agarose gels were stained with ethidium bromide and visualized using a UV imager. The fractions of target cleaved were quantified using ImageJ software (v1.43u). The band intensity for the cleavage product band was divided by the combined intensity of cleavage product and uncut substrate bands and reported as fraction cleaved (i.e. “cut” / “cut + uncut”). Time course cleavage assay results were plotted using GraphPad Prism software and fit to a two-phase association equation, which was used to calculate maximum cleavage (Cleav_max_) and time required to achieve 50% Cleav_max_ (t_½_). An average observed rate constant (*k*_obs_) was calculated using *k*_fast_, *k*_slow_, and %Fast parameters generated in Prism after non-linear regression with two-phase association using the equation *k*_obs_ = [((*k*_fast_)*(%Fast))+((*k*_slow_)*(%Slow))]/100, which can be considered the mean reaction time [51].

### Dot-blot filter binding assays for dCas9 target DNA binding

Synthetic crRNA and target double-stranded DNA lack 5′ phosphates and were directly radiolabeled. One hundred pmol of tracrRNA, sgRNA, or target strand DNA was radiolabeled with [γ-^32^P]-ATP using T4 Polynucleotide Kinase (New England Biolabs) following the manufacturer’s recommended protocol. Reactions were phenol–chloroform extracted, and radiolabeled RNA or DNA was gel-purified on 15% denaturing polyacrylamide gels (1× TBE, 7 M urea) by the crush-and-soak method. Gel-purified radiolabeled RNA and DNA were quantified by scintillation counting.

The active concentration of dCas9 was empirically determined as previously described [52]. Briefly, increasing amounts of dCas9 were titrated with 40 pmol (1 µM final) of cold (unlabeled) crRNA:tracrRNA complex in which a negligible amount of radiolabeled crRNA (1000 cpm) was spiked. Dot blots were performed to identify the volume of dCas9 corresponding to 40 pmol (complete binding of crRNA) in the reactions described. For target DNA binding by dCas9 RNP complexes, radiolabeled duplex target DNA (1000–1500 cpm/reaction) was combined with increasing concentrations of a pre-assembled dCas9-tracrRNA-crRNA or dCas9-sgRNA complex in a final reaction of 60 µl in 1× cleavage buffer (see above) and 0.1 mg/ml tRNA. After incubation at 37°C for 15 min, reactions were vacuum filtered over nitrocellulose membrane (Protran Premium NC, Amersham) using a 96-well dot blot apparatus. Wells were washed twice with 200 µl of 1× cleavage buffer. Membrane was then removed and washed with 1× phosphate-buffered saline (PBS) and air-dried at room temperature. Binding of radioactive target DNA was then visualized by phosphorimager. Spots were quantified with ImageQuant software, plotted in Prism (GraphPad), and fit to a one-site binding hyperbola equation. Error bars for all quantified data represent experimental replicates, not technical replicates. Sample size was selected based on the expectation that three or more replicates will be representative.

### Single molecule target DNA binding by dCas9

For RNP assembly, 36 nM of dCas9 and 30 nM of Cy3-sgE2 were mixed in 20 mM Tris–HCl (pH 7.5), 100 mM NaCl, 0.5 mM MgCl_2_, and 0.5 mM DTT. The sample was then incubated at 37°C for 10 min. For dual guide experiments, the Cy3-sgE2 was replaced with 30 nM of Cy3-crE2 and 30 nM of tracrRNA. While the RNP sample was incubated, 21.1 nM of pSMART-EGFP-Cut1c plasmid [50] was mixed in 12 mM Tris-acetate (pH 7.5), 2.5 μM yeast tRNA, 0.2% Tween-20, 2.5 mM protocatechuic acid (PCA), 50 nM protocatechuate 3,4-dioxygenase (PCD), and 2 mM Trolox. The PCA/PCD oxygen scavenging system and Trolox are added for anti-bleaching and photostability. For control experiments, the pIDT-EGFP-Cut1c plasmid was replaced by an unrelated plasmid of similar molecular weight (pTRE3G plasmid), which does not include the EGFP target sequence.

Microscopy setup, data acquisition, and image analysis were performed using convex lens-induced confinement (CLiC) microscopy as described previously [53]. After incubation, the RNP sample was diluted to 750 pM in the plasmid sample, mixed, and then flowed using positive air pressure into the CLiC device. The sample enters a flow cell consisting of two coverslips separated by 10 µm double-sided spacer tape (Nitto Denko). The bottom coverslip contains an array of 3 µm diameter wells with 500 nm depth that were etched into the coverslip using microfabrication methods [53], while the top coverslip is featureless. In this microscopy technique, a convex lens pushes down on the top coverslip, deforming it, and bringing it into contact with the bottom coverslip. When the top coverslip contacts the bottom, it traps the molecules in the wells, allowing them to diffuse freely while simultaneously keeping them within the field of view. The sample was kept at 37°C by heaters attached to both the convex and objective lenses. Videos were taken at an exposure of 30 ms once every minute for ~1 h. In between video acquisitions, the top coverslip was raised, allowing new molecules to diffuse into the field of view to replace the previously viewed molecules, and then brought back into contact with the bottom coverslip.

RNPs bound to plasmids were manually counted in each video and then plotted versus time. Bound RNPs were distinguished from free RNPs by differences in their diffusion, size, and intensity. RNPs bound to plasmids appear on video as a slowly diffusing, diffraction-limited spot, while free RNPs appear as fast-moving blurs that uniformly illuminate the wells. Clumps of RNPs were ignored in the binding count. Clumps were identified by size and intensity, as they were larger and had more fluorophores than the singly labeled bound RNPs. Dark or dim pits outside of the laser beam were excluded from analysis to avoid under-counting molecules. To determine the cut-off position, a chi-squared test was used to compare the total number of molecules detected in each pit over a dataset (∼60 samplings) to an expected uniformly distributed set of molecules in those same pits. A cutoff radius was selected that optimized the chi-squared value while maximizing statistics and excluding any molecules beyond that radius from analysis. Because the pit positions are not constant between datasets, results were normalized per 100 pits.

### Thermal denaturation monitored by UV absorbance

Cas9 alone or assembled as an RNP at 1 µM final concentration (equimolar concentration of all components) was incubated at room temperature for 10 min in degassed 1× UV melt buffer (20 mM sodium cacodylate, pH 7.5, 150 mM KCl, 1 mM MgCl_2_). Samples were melted in a Cary 400 UV/Vis spectrophotometer at a ramp rate of 1°C/min while UV absorbance at 280 nm was collected every 1 min. Experiments were repeated in duplicate. Melting temperatures were determined using Van’t Hoff calculations and error determined by standard error of the mean using two experimental replicates for each sample.

### Generation of HEK 293T cells stably expressing EGFP

HEK 293T cells stably expressing EGFP (known as EGIP) were a kind gift from Dr Wen Xue (Yin, H. *et al*. 2017). In addition, we used CRISPR-based AAVS1 safe harbor knock-in for generating HEK 293T cells stably expressing an alternative codon-optimized version of EGFP. The EGFP gene was synthesized as a gBlock (Integrated DNA Technologies, IDT) with 15 bp extensions complementary to the ends of pAAVS1-EF1a-BSD-DNR vector (ORIGENE, GE100048) after digestion with KpnI (Thermo Scientific). Insertion was accomplished using the In-Fusion HD Cloning Kit (Clontech/Takara) following the manufacturer’s recommended protocol. Ligated vector was then transformed into NEB stable cells following the manufacturer’s recommended protocol. Single colonies were selected from LB agar-carbenicillin plates and plasmid prepared by midi-prep. A guide sequence targeting AAVS1 locus was synthesized by IDT as two complementary oligonucleotides and annealed to prepare a DNA insert. The pSpCas9 (Addgene 62988) vector was digested with FastDigest BbsI (Thermo Scientific) and the DNA oligo encoding the sgRNA was inserted into the linear vector using T4 DNA ligase (Thermo Scientific) following the manufacturer’s recommended protocol. All clones were verified by Sanger sequencing. HEK 293T cells were co-electroporated (Invitrogen Neon transfection system) with pAAVS1-EF1a-BSD-DNR carrying the EGFP donor template and pSpCas9 expressing sgRNA and Cas9 enzyme. Cells were passaged for 3 weeks before antibiotic selection to dilute cells containing the donor in non-stable episomal form. Cells were treated with 5 µg/ml of blasticidin for 2–3 days to enrich edited cells. Cells were further selected for EGFP fluorescence with fluorescence-assisted cell sorting.

### Cell-based editing quantified by flow cytometry

HEK 293T cells expressing EGFP versions were grown in Dulbecco’s modified eagle’s medium (DMEM) with 1× non-essential amino acids (NEAA), 5% cosmic calf serum (CCS), and 5% fetal bovine serum (FBS) without antibiotics. HEK 293T cells (3 × 10^6^) were electroporated (Invitrogen Neon transfection system) with 12 µg (200 ng/5 × 10^4^ cells) of SpCas9-expressing plasmid (pLentiV_Cas9_Puro, Addgene, 108100) in a T75 flask and incubated in a 5% CO_2_ incubator for 36 h in order to allow the plasmid to transiently express SpCas9 prior to guide RNA transfection. SpCas9 mutant plasmids were prepared by site-directed mutagenesis as described above using the pLentiV_Cas9_Puro vector. HEK 293T cells expressing SpCas9 or SpCas9 mutants and EGFP (5 × 10^4^ cells) were reverse transfected in four replicates with 10 pmol of crRNA:tracrRNA complex or sgRNA using RNAiMAX (Invitrogen) following the manufacturer’s recommended protocol. Opti-MEM was replaced with full media after 12 h and cells were allowed to grow for an additional 4 days. Cells were then trypsinized, washed with 1× PBS and resuspended in 200 µl of 1× PBS for flow cytometry analysis (Attune NxT Focusing Cytometer, Thermo Fisher). EGFP was detected using the blue laser (BL1 channel) for at least 20 000 events and analyzed by Attune software (v4.12). A non-transfected control was used for gating (Supplementary Fig. S1) and to calculate the total editing efficiency (loss of EGFP) of guide RNAs transfected.

### Cell-based editing quantified by rhAmpSeq

Spacer sequences were selected by retrieving the top pre-designed guide RNA spacer sequences from the first alphabetically ordered 2000 genes from IDT’s gRNA design tool (https://www.idtdna.com/site/order/designtool/index/CRISPR_PREDESIGN). A sub-selection of 255 spacers were identified for an even distribution of the following attributes: PAM sequence, on-target and off-target scores (from IDT’s gRNA design tool), homopolymer stretches, and spacer sequence GC content (Supplementary Table S2). Alt-R crRNAs and sgRNAs generally contain 2′-O-methyl and phosphorothioate-modified termini to protect from exonucleases. Dual guide RNAs were prepared by mixing equimolar amounts of crRNA and tracrRNA in IDT duplex buffer, heating to 95°C, and slow-cooling to room temperature. Single guide RNAs were prepared by rehydrating in IDT duplex buffer. Ribonucleoprotein complexes were formed by combining the CRISPR–Cas9 nuclease (Alt-R SpCas9 Nuclease V3, IDT) and the guide RNA in a 1:1.2 molar ratio and incubating at room temperature for 30 min. RNPs were delivered into Jurkat cells via electroporation as described previously [33]. Editing events were quantified using the rhAmpSeq system (IDT), Illumina sequencing, and counting of reads with indels, as described previously [33, 54].

### On-target and off-target editing quantified by nanopore amplicon sequencing

HEK 293T cells expressing EGFP were grown and transfected with Cas9 plasmid and guide RNAs as described above for cell-based editing and quantification by flow cytometry. However, 1 × 10^5^ cells were reverse transfected in three replicates with 40 pmols of crRNA:tracrRNA complex or sgRNA using RNAiMAX (Invitrogen) following the manufacturer’s recommended protocol in a 48-well plate. Opti-MEM was replaced with full media after 12 h and cells were allowed to grow for an additional 3 days. Cells were then trypsinized, washed with 1× PBS and processed for genomic DNA extraction using a Monarch Genomic DNA purification kit (#T3010L) following the manufacturer’s recommended protocol. DNA was quantified using Qubit dsDNA BR kit.

For each sample, PCR reactions were generated with a set of primers designed to generate ∼1000 bp amplicons with an on- or off-target Cas9 cut site near the center of the amplicon (Supplementary Table S3). PCR was performed in standard Q5 polymerase (New England Biolabs, NEB) conditions, with 0.5 μM final concentration (unless otherwise noted) of primers and ~30 ng of genomic DNA/sample. Each PCR reaction was 22 cycles of 98°C for 10 s, 67°C for 30 s, and 72°C for 60 s. Primers for each site are listed below, as well as any deviations from the 0.5 μM final concentration to obtain more equivalent amplicon representation when using multiple primer sets in a single PCR reaction.

Standard nanopore protocol for amplicon barcoding (Native Barcoding Expansion 96 Kit, Oxford Nanopore Technologies, ONT) was followed. Briefly, PCR amplicons are concentrated and purified via SPRI bead cleanup. Amplicons are then “end-prepped” to add a single deoxyadenosine (dA) tail to each fragment with Ultra II enzyme (NEB). Unique barcodes with deoxy thymidine (dT) tails are then ligated to amplicons in each sample. Barcoded amplicons are then pooled, and the overhangs on the ligated barcodes are used to ligate a sequencing adapter. This adapter-ligated and barcoded sample was sequenced on a PromethION P2 solo using MinKNOW (v24.02.16) following standard nanopore protocol. During sequencing and base-calling, quantification of each amplicon in sample is accomplished using RAMPART software (https://github.com/artic-network/rampart), with a goal of at least 200× coverage of each amplicon in each sample. After sequencing, percentage of amplicons with Cas9 activity is calculated using the CRISPResso WGS function of the CRISPresso2 software suite (https://github.com/pinellolab/CRISPResso2), then normalized against a control sample with no Cas9 activity. Commands and scripts available upon request.

### *In vitro* sequence preference determination with partially randomized target DNA and guide RNA

Partially randomized 200 nt ssDNA ultramers were obtained from Integrated DNA Technologies (IDT). DNA amplification of ssDNA ultramers was conducted using the forward primer pEGIP_5C3_F: /5SpC3/ATGCGATGGAGTTTCCCCA and the reverse primer pEGIP_5C3_R: /5SpC3/CCGCTTTACTTGTACAGCTCG. PCR reactions (25 µl total volume) contained 50 pmol of ssDNA ultramer, 0.5 µM of each primer, 200 µM of dNTPs, 0.02 U/µl of Q5 Hot Start High-Fidelity DNA Polymerase, and 1× Q5 reaction buffer (NEB). The PCR cycling conditions were as follows: 98°C for 25 s, 25 cycles of 94°C for 8 s, 65°C for 10 s, and 72°C for 8 s, followed by a final extension at 72°C for 30 s. Following PCR amplification, 1 µl (20 U) of Exonuclease I (NEB) was added and incubated at 37°C for 20 min to remove primers and ssDNA. The PCR reactions were bead purified using 3:1 ratio of AMPure XP beads (Beckman Coulter) to reaction volume following the manufacturer’s instructions and eluted in nuclease-free water. Purified PCR products were quantified using Qubit 1× dsDNA High Sensitivity (HS) Assay Kit (ThermoFisher Scientific) according to manufacturer’s protocol. The PCR products were visualized on a 2%–3% agarose gel stained with ethidium bromide and imaged under UV illumination.

*In vitro* cleavage assays were performed as previously described [32, 50] with minor changes. Two versions of PCR amplified targets (E2 and E2-Rdm8 or E4 and E4-Rdm8) were used to assess the intrinsic enzyme activity of Cas9 loaded with regular or partially randomized guide RNAs. Briefly, a master mix containing final concentrations of 0.32 µM Cas9, 0.32 µM tracrRNA, 0.32 µM crRNA, or 0.32 µM Cas9 and 0.32 µM sgRNA in a 1× cleavage buffer (20 mM Tris–HCl, pH 7.5, 100 mM KCl, 5% glycerol, 1 mM DTT, 0.5 mM EDTA, 2 mM MgCl_2_) supplemented with 0.1 mg/ml of purified yeast tRNA was incubated at 37°C for 10 min to allow the RNP to assemble. Target DNA (2.73 µl of 100 ng/µl) was added to 77.27 µl of pre-assembled RNP and incubated at 37°C, and reactions were stopped by phenol-chloroform extraction and ethanol precipitation.

Library preparation was performed using PCR Barcoding Kit (SQK-PCB111.24, ONT) and Native Barcoding Kit 24 V14 (SQK-NBD114-24, ONT). Target DNA cleavage products were end-prepped using 0.75 μl Ultra II End-prep Enzyme mix (NEB) and 1.75 μl Ultra II end-prep reaction buffer (NEB). Reactions were incubated at 20°C for 10 min followed by 65°C for 10 min using a thermal cycler. The PCR products were purified using 3:1 ratio of AMPure XP beads (Beckman Coulter) to reaction volume and eluted in 16 μl of elution buffer (Omega Bio-Tek). Each sample was quantified using Qubit 1× dsDNA High Sensitivity (HS) Kit Assay (Thermo Fisher Scientific). For barcode adapter ligation, 15 μl of end-prepped DNA was mixed with 10 μl of the barcode adapter (BCA) and 25 μl of Blunt/TA Ligase Master Mix (NEB) and incubated at room temperature for 30 min. The BCA-ligated DNA was purified using 3:1 ratio of AMPure XP beads (Beckman Coulter) to reaction volume and eluted in 23 μl. The DNA samples were PCR amplified using 1 μl of BC barcode, 1 μl of the forward pEGIP_F primer (0.4 μM), and 25 μl of LongAmp Taq 2× Master Mix (NEB). The PCR cycling conditions were: 95°C for 25 s, then 13 cycles of 94°C for 8 s, 62°C for 10 s, and 65°C for 10 s, followed by a final extension at 65°C for 30 s. PCR reactions were purified using 3:1 ratio of AMPure XP beads and eluted in 16 of elution buffer (Omega Bio-Tek). The PCR-amplified samples underwent a second end preparation as previously described and were eluted as previously described in 10 μl of elution buffer (Omega Bio-Tek). The cleaned-up PCR barcoded samples were quantified using Qubit 1× dsDNA High Sensitivity (HS) Assay Kit (Thermo Fisher Scientific) and equimolar amount of DNA for each sample was taken forward for native barcoding. Individual end-prepped DNA was barcoded using Native Barcoding Kit 24 V14 (SQK-NBD114-24) by combining 7.5 μl of end-prepped DNA, 2.5 μl of Native Barcode, 10 μl of Blunt/TA Ligase Master Mix (NEB) and incubated at room temp for 20 min. The reactions were stopped by adding 2 μl of 0.5 M EDTA and pooled together. The pooled samples were purified using a 2.8:1 ratio of AMPure XP beads (Beckman Coulter) to reaction volume eluted in 30 μl of elution buffer (Omega Bio-Tek). Adapter ligation was performed by combining 30 μl of pooled barcoded DNA, 5 μl of native adapter (NA), 10 μl of 5× NEBNext Quick Ligation Reaction Buffer (NEB), and 5 μl of Quick T4 DNA Ligase (NEB) and incubated at room temperature for 30 min. The reactions were purified using 2.2:1 ratio of AMPure XP beads and washed with Short Fragment Buffer (SFB). The final library was loaded on a PromethION R10.4.1 DNA flow cell and sequenced on a PromethION P2 solo using MinKNOW (v24.02.16).

POD5 files were base-called using Dorado (v0.8.1) with the dna_r10.4.1_e8.2_400bps_sup@v5.0.0 model, using the parameters no-trim and minimum q-score set to 8. The base-called reads were demultiplexed using Dorado demux (v0.8.1) with the SQK-PCB111.24 and no-trim parameters. Custom Python scripts utilizing the Biopython SeqIO module were used to identify reads matching the expected cleaved TCAAGGT and uncleaved TCACCG sequences as well as their reverse complements. For each matched read, 30 nt upstream or downstream of the match were retained as flanking sequence. The reads were subsequently filtered to retain only those of a uniform length and further filtered to retain sequences upstream of the randomized region, containing CACCATGGTGAGGCAA in the forward or TTGCTCACCATGGTG as its reverse complement. Seqtk (v1.4) (https://github.com/lh3/seqtk) was used to generate reverse complements for consistent orientation in downstream analyses. The filtered forward and reverse DNA sequences were concatenated and then trimmed to retain only the initial 8 nucleotides at the 5′ end of the gRNA binding site on the DNA target. WebLogos of fasta files were generated using WebLogo 2 [49] to produce probability-based logos.

### Cryo-EM structural studies

Cas9 was assembled with RNA guides and DNA target substrates (ternary complexes) at room temperature for 15 min in 20 mM Tris, pH 7.5, 200 mM KCl, 0.5 mM DTT, and 5 mM EDTA. Complexes were prepared at a final of 1.0 to 1.6 mg/ml, placed on ice, and grids prepared within 2–3 h. Cryo-EM grids were Quantifoil 2/2 holey grids, glow-discharged prior to vitrification that was carried out on an FEI Vitrobot Mark IV (FEI) at Washington University Center for Cellular Imaging (St. Louis). Each grid was inspected on a 200-kV Glacios equipped with a Falcon IV direct electron detector, and preliminary datasets were collected for 2–3 h for each grid with acceptable density of particles and lacking visible aggregates. For both sgE2 and dgE2 complexes, the optimal concentration was found to be at 1.4 mg/ml. Final datasets for the best grids were collected on a 300-kV Titan Krios G3 cryo-TEM equipped with a GIF BioQuantum 968 energy filter and a Gatan K3 direct electron detector. 5 008 movies were collected for sgE2 complex with pixel size of 0.7 Å, total dose of 51.37 e/Å^2^, 49 frames, and acquisition time of 4.28 s. Two data sets with a total of 11 890 movies were collected for the dgE2 complex with similar parameters.

Images were processed with cryoSPARC software. Motion-corrected and ctf-estimated images were curated, resulting in 4393 movies for the sgE2 complex and 9251 movies for the dgE2 complex. Several rounds of iterative particle picking using blob and template picking routines followed by the topaz training method and particle selection using 2D classification, *ab initio* modeling, 3D refinement, and 3D classification were performed for each data set. For the sgE2 complex, the best resolution of 3.12 Å was obtained from a final set of 64 788 particles using “Local Resolution” refinement.

In the case of the dgE2 complex, processing of the first data set (5714 total movies, 4345 curated movies) yielded a map of 3.31 Å resolution calculated from 105 360 particles using non-uniform refinement. The refined structure represented a “closed” conformation similar to that of the sgE2 complex. Notably, unlike sgE2 data, processing of dgE2 data consistently yielded high-contrast 2D classes corresponding to a proposed “open” conformation of the protein alone or in complex with guide RNA only. 3D maps for this class were of lower resolution, above 4 Å, which permits placing major structural domains of Cas9 but not fine nucleic acid detail. In an attempt to improve resolution, we collected a second data set of 6176 movies from the same grid. 4906 movies were selected after curation. Additional data slightly improved resolution of the map for the closed form to 3.26 Å using 215 418 particles and local refinement. These particles were subjected to 3D classification routine implemented in cryoSPARC v4.1.2. Three identical maps of highest resolution were used as input. One class of 108 048 particles yielded a map of an improved resolution of 3.11 Å. Additional polishing steps of global CTF refinement further improved resolution to 3.03 Å.

Additional data collection did not offer improved resolution of an open form. The highest obtained resolution was for 3.67 Å for 141 467 particles with an overall B-factor of 140 Å^2^.

DeepEMhancer maps were generated to aid modeling and refinement. Models were built and refined using the Refmac program implemented in Phenix combined with manual modeling using Coot. A crystal structure (PDB ID: 4UN3) was used as a starting model for building and refinement of the sgE2 structure. Refined sgE2 structure was modified and used for model building and refinement of the dgE2 structure. PDB ID 7S3H was used for modeling of dgE2 in an open conformation. Only partial RNA strands were modeled into density, including nucleotides 40–70 of crRNA, in a conformation similar to those of corresponding sgRNA loops in 7S3H, as well as 7–26 of crRNA and 21–38 of tracrRNA, which were not previously observed in open-conformation complexes. Final structures and experimental data were deposited in PDB and EMBD with PDB ID: 8G1I and EMD-29671 for sgE2; and PDB ID: 8FZT and EMD-29639 for dgE2 in closed conformation; PDB ID: 9B2K and EMD-44111 for dgE2 in open conformation.

### Protein and nucleic acid model generation

The single and dual RNA-guided Cas9 systems with protein in the catalytically active state were generated for computational studies. The model was prepared in MODELLER [55] using crystal structures in PDB files 5F9R [56] and 5Y36 [57] as templates. The 5F9R structure corresponds to a precatalytic state of the Cas9, in which the HNH domain is away from the target DNA strand, and in the pseudoactive 5Y36 structure, the HNH domain is very close to the target strand. The precatalytic model of Cas9 cannot capture the structural features associated with the cleavage of the DNA, which is required for the current study. But the nucleic acid strands are more complete in the precatalytic crystal structure. Therefore, a chimeric protein model using the above crystal structures was created, and then 5 rounds of steepest descent minimization (5000 steps) and 5 rounds of conjugate gradient minimization (5000 steps) were carried out. Five low energy structures generated after minimization were subjected to short 5 ns MD simulations, where harmonic restraints were applied to the protein backbone atoms. Finally, the model with low energy structure corresponding to the catalytic state was used for further studies. The nucleic acid coordinates were obtained from the 5F9R crystal structure. The sequences were changed as per the experimental spacer sequences E1, E2, and E3 in the UCSF Chimera program [58]. The loop of the single guide system was shortened (by removing 9 base pairs) to match the experimental sequence. To prepare the dual guide system, the first loop was removed (4 bases) and refined from the template nucleic acid structure. The catalytic Mg^2+^ ions were retained from the 5Y36 model. This model generation protocol finally yielded three different models of E1, E2, and E3 sgRNA and dgRNA systems.

The catalytic model of the protein and refined nucleic acid structures were then combined in tleap. The Amber ff14SB [59] force field for protein, the parmbsc1 force field [60] for DNA, and the ff99bsc0*+χOL3* force field [61] for RNA were used. The TIP3P model has been employed for water. The system was solvated using a 10 Å rectangular water box, and NaCl salt concentration of 100 mM was added to the complexes in tleap.

### Molecular dynamics and energy calculations

The molecular dynamics protocol previously reported by Palermo and co-workers [62] was used with slight modifications. The system was initially subjected to 10 000 steps (5000 steps of steepest descent along with 5000 steps of conjugate gradient) of minimization with a restraint of 300 kcal/mol·Å^2^ on the protein, nucleic acid, and Mg^2+^ ions. Then the restraints were removed, and the complexes were again minimized for 10 000 steps (5000 steps of steepest descent along with 5000 steps of conjugate gradient). The time step used for the simulation was 2 fs and SHAKE algorithm was applied to the bonds containing hydrogens. The cutoff for long-range electrostatic interactions was 10 Å. The heating was done in four stages where systems were heated up from 0 to 50 K and 50 to 100 K by running two NVT ensemble simulations of 50 ps each, imposing restraints of 100 kcal/mol·Å^2^ on the protein, RNA, DNA, and Mg^2+^ ions. The temperature was then increased to 200 K in 100 ps using NVT ensemble in which the restraint was reduced to 25 kcal/mol·Å^2^. Finally, the system temperature was raised to 300 K in an NPT-ensemble simulation of 500 ps without any restraints. One nanosecond of NPT-ensemble equilibration was done on the system, followed by a test production run of 10 ns NPT ensemble. After observing the 10 ns trajectory of the system, production runs were extended to 100 ns using NVT ensemble in the GPU-accelerated version of PMEMD [63, 64] in AMBER 18 [65]. The final structure after the 100 ns MD was used as the starting point of the Gaussian accelerated molecular dynamics (GaMD) [66]. A dual-boost scheme was used for the GaMD. Approximately 90 ns of GaMD equilibration followed by 500 ns of GaMD production were carried out for all the six systems. The boost potential was updated every 1.6 ns.

The six trajectories were then analyzed using the CPPTRAJ [67] module of AmberTools 19 and VMD 1.9.4. [68]. The RMSD, RMSF, and distance calculations were carried out in the CPPTRAJ module. The cutoff for hydrogen bond calculation was 3.5 Å and 135. The potential of mean force (PMF) is calculated upon accurate reweighting of the simulations using cumulant expansion to the 2nd order. The reweighting was carried out using the PyReweighting toolkit [69]. The parameters were altered to match our calculations. The PMF calculations were performed on the GaMD trajectories using amino acid distances, which were previously determined experimentally and can characterize the catalytic state of the protein. The convergence of the simulation was checked by calculating the PMF plots by varying bin sizes.

The MMGBSA [70] calculations with per residue energy decomposition were carried out using the GaMD trajectory, considering every 100 frames. The MMPBSA.py script in AmberTools 19 was used for the purpose. The energy decomposition can be represented as the following:


\begin{eqnarray*}
{\mathrm{Energy\ of\ Base\ X}} &=& {\mathrm{Sum\ of\ internal\ energy\ of\ base\ X}}\\&& +\, {\mathrm{Sum\ of\ Interaction\ with\ bases}} \\&&+\, {\mathrm{Sum\ of\ interaction\ with\ amino\ acid}} \\&&\, {\mathrm{\ residues\ (cutoff\ 6\ }}{\mathring{\rm A}}{\mathrm{)}}.
\end{eqnarray*}

Further, difference between the energy of the same base in the single and dual guide systems was calculated as:


\begin{eqnarray*}
{\mathrm{\Delta \Delta }}{E}_{sg - dg} &=& {\mathrm{\Delta }}{E}_{{\mathrm{sg}}} - {\mathrm{\Delta }}{E}_{{\mathrm{dg}}};{\mathrm{\Delta }}{E}_{{\mathrm{sg}}}{\mathrm{\ is\ the\ per\ residue\ energy\ of}} \\&& {\mathrm{\ the\ bases\ in\ the\ sg\ and\ \Delta }}{E}_{{\mathrm{dg}}}{\mathrm{\ is\ the\ per}}\\&&{\mathrm{\ residue\ energy of\ the\ bases\ in\ the\ dg}}.
\end{eqnarray*}

The trajectory was visualized in UCSF Chimera and the images were rendered using PyMOL 2.4.1.

## Results

### Guide RNA architecture controls Cas9 activity

**Figure 1.**
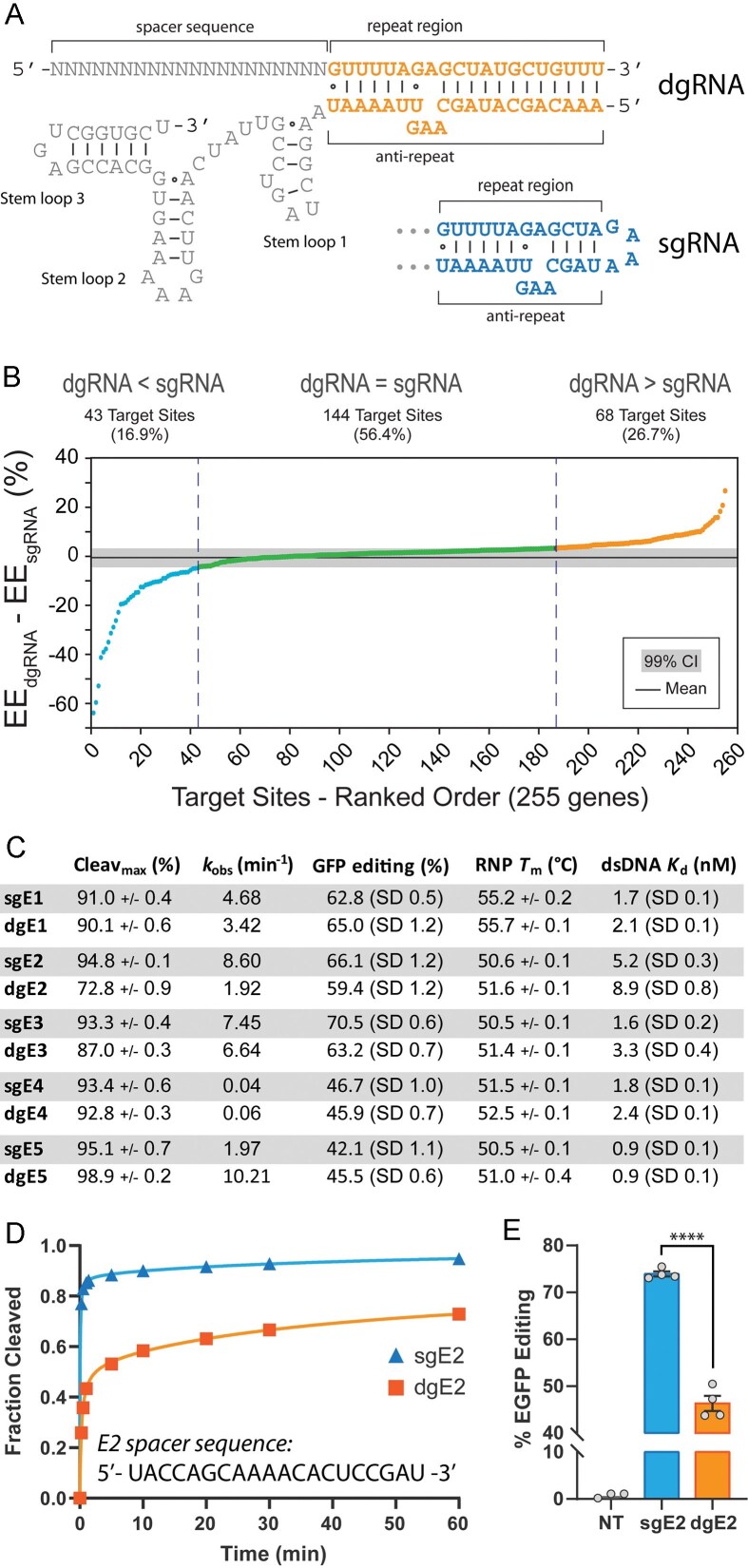
Single and dual guide Cas9 complexes exhibit differential gene editing and target cleavage activity depending on guide spacer sequence. (**A**) Schematic representation of dgRNA and sgRNA secondary structures. (**B**) Cell-based editing at 255 endogenous loci with matched single guide (sg) or dual guide (dg) CRISPR–Cas9. The difference in editing efficiency (EE) between dgRNA and sgRNA is plotted in ranked order across all sites. (**C**) Summary of *in vitro* cleavage activity (Cleav_max_ and *k*_obs_) (*n* = 2), editing of an EGFP reporter gene in cells (*n *= 4), ribonucleoprotein thermal stability (RNP *T*_m_) (*n* = 2), and target DNA binding affinity (dsDNA *K*_d_) (*n* = 2) for matched sg and dg CRISPR–Cas9 complexes using five model spacers, E1 through E5. Values presented are derived from data shown in panels (**D** and **E**) and Supplementary Figs S3 and S4. Range of values (±) or standard deviation (SD) is reported. (D) Comparison of sgRNA- and dgRNA-guided Cas9 with an E2 spacer sequence in *in vitro* time course cleavage assays (*n* = 2) and (E) cell-based EGFP editing assays (*n* = 4). Error is reported as SEM. Unpaired t-test *P*-value: ^****^ >.0001. NT, no treatment.

We began by comparing editing between dgRNA and sgRNA (Fig. 1A) across a large set of endogenous target sites. Using a commercially available chemically modified guide RNA system (Alt-R CRISPR–Cas9), which incorporates 2′-O-methyl and phosphorothioate modifications near the guide RNA termini to improve nuclease resistance, we measured editing at 255 endogenous loci with matched dgRNA and sgRNA spacers delivered as ribonucleoproteins (RNPs). Of the 255 spacers, 68 (26.7%) edited more efficiently as dgRNAs, 43 (16.9%) edited more efficiently as sgRNAs, and the remaining 144 (56.4%) performed comparably (Fig. 1B and Supplementary Table S2). To validate these observations with defined reagents, we selected 15 spacers spanning the range of architecture preferences and resynthesized them as native, unmodified guides (Supplementary Fig. S2A). We found that chemical modification generally and moderately improved editing efficiency for all fifteen guides but did not generally change their spacer-dependent editing preferences (*R*^2^ = 0.95) (Supplementary Fig. S2B). Results were similar for the unmodified guides: six edited better as dgRNAs, five performed similarly, and four were better as sgRNAs (Supplementary Fig. S2C). Because guide RNA architecture was the only variable across these matched comparisons, these results establish that dgRNAs can match or exceed sgRNA editing and reveal spacer-dependent differential activity between the two guide RNA architectures.

To explore differential activity in greater detail, we selected five new spacers, E1 through E5, that target an EGFP gene [32, 39, 50]. We determined maximum cleavage at 60 min (Cleav_max_) and apparent observed rate constants (*k*_obs_) with *in vitro* cleavage assays, as well as gene editing in HEK 293T cells via knockout of an EGFP reporter (Fig. 1C). E1 and E4 guides displayed nearly identical cleavage kinetics and EGFP knockout for both guide architectures, although E4 yielded slower kinetics (Fig. 1C and Supplementary Fig. S3). E2 and E3 sgRNAs had higher cleavage and EGFP editing (Fig. 1C–E and Supplementary Fig. S3), while dgRNA was more active for E5 (Fig. 1C and Supplementary Fig. S3). The E2 guide showed the greatest divergence in activity, highlighting the impact of guide RNA architecture on Cas9 function (Fig. 1D and E).

Single and dual guides assemble into two and three component RNPs, respectively, suggesting that global stability of the complex may affect activity. Therefore, we compared RNP melting temperatures (*T*_m_) by monitoring Cas9 stability with 280 nm absorbance [32] and using E1 through E5 guides. RNP thermal stability was quite similar for the two guide architectures (Fig. 1C and Supplementary Fig. S4). We also performed limited trypsin hydrolysis of Cas9 bound to sgE2 or dgE2 and a complementary DNA target [71] but observed no obvious changes in global trypsin cleavage patterns of Cas9 (Supplementary Fig. S5). These results suggest that global stability or large structural alterations do not drive differential activity [72]. To test whether guide architecture alters target binding affinity, we determined dissociation constant (*K*_d_) values for target DNA interaction using equilibrium filter-binding assays (Fig. 1C and Supplementary Fig. S4). Though *K*_d_ values from bulk equilibrium binding did not reveal clear differences, dgE2 binding affinity was notably reduced compared to sgE2 (Fig. 1C).

To determine if stability of the guide RNA repeat structure itself affects activity, we chemically modified the tracrRNA (for dgRNA architecture) with two locked nucleic acid (LNA) residues near the 5′ terminus, thereby increasing the hybridization affinity of crRNA and tracrRNA in the repeat duplex [73] (Supplementary Fig. S6A). The global stability for Cas9 RNP when assembled with modified tracRNA was unchanged compared to unmodified tracrRNA (Supplementary Fig. S6B). However, the modified tracrRNA conferred EGFP editing efficiencies that trended toward that of the sgRNA, typically falling between dgRNA and sgRNA (Supplementary Fig. S6C). These results suggest that differential activity is linked to repeat structure stability or dynamics.

### Spacer sequence and predicted guide RNA structure poorly predict target preference

**Figure 2.**
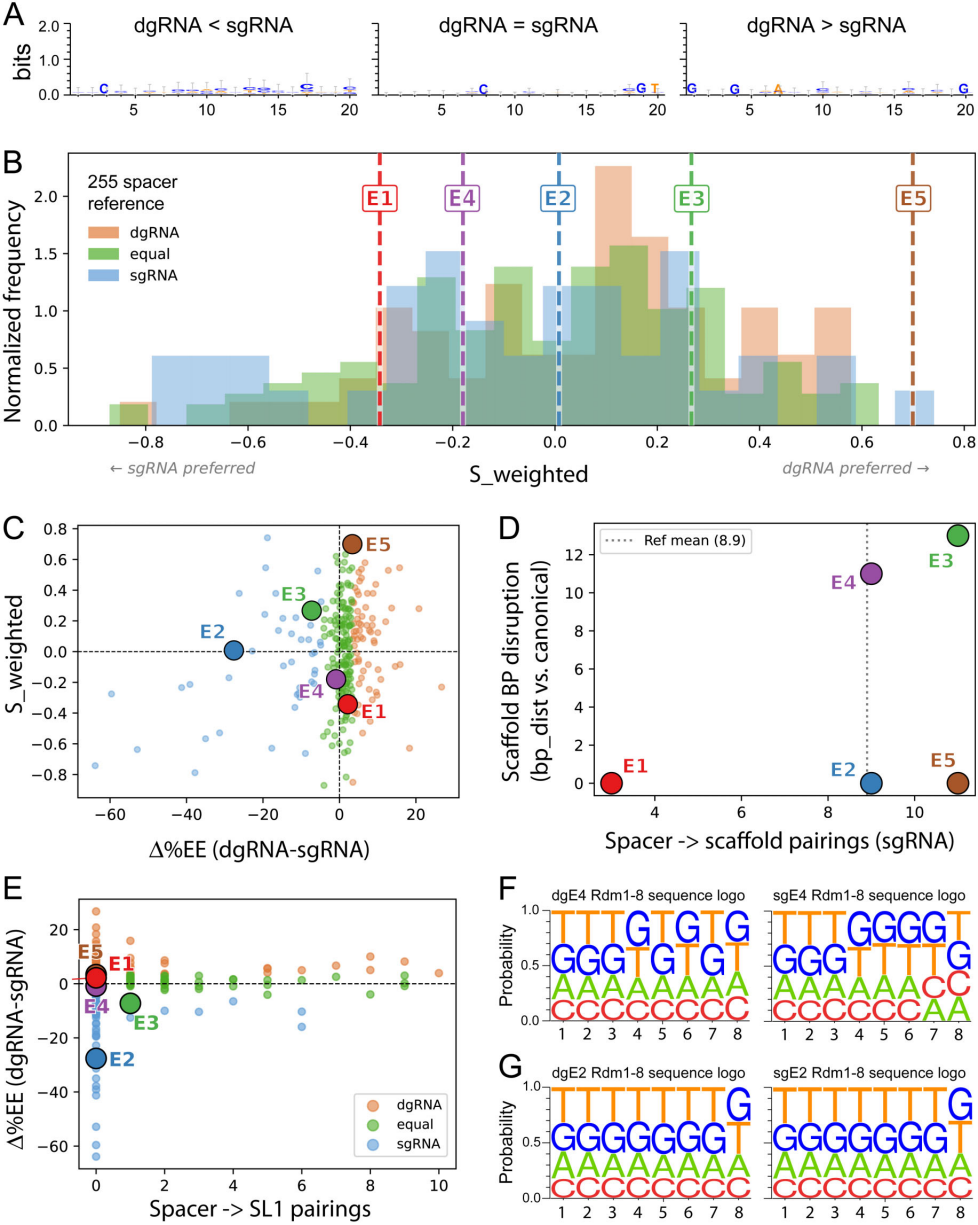
Sequence and predicted structural features of sgRNA and dgRNA correlate poorly with editing efficiency and target preference. (**A**) Sequence logo prediction using WebLogo 3 with spacers and editing results from 255 endogenous loci. Generated logos do not indicate strong sequence preferences across spacer classes. (**B**) Normalized frequency distribution of the composite structural score (S_weighted) across the 255 endogenous spacers for sgRNA and dgRNA, separated by experimentally determined preference. Bar height represents the proportion of spacers per unit score interval, normalized so total area sums to 1.0. Dashed vertical lines indicate S_weighted values calculated for EGFP guides E1–E5. (**C**) Scatter plot of S_weighted versus differential editing efficiency (Δ%EE = dgRNA − sgRNA) for all 255 reference spacers, with EGFP guides overlaid as labeled circles. (**D**) Scaffold base-pair disruption (bp distance from the canonical scaffold fold in the sgRNA context) versus the number of spacer nucleotides pairing to the sgRNA scaffold, for EGFP guides E1–E5 relative to the 255 reference spacer mean. (**E**) Spacer–SL1 pairing (sgRNA scaffold) count versus Δ%EE for all 255 reference spacers, with EGFP guides highlighted. (**F, G**) Dual randomization of the 8 PAM-distal nt of E4 and E2 dgRNA and sgRNA with target DNA to identify potential sequence preferences. *In vitro* cleavage products were sequenced to identify nt preferences at each position. Mean sequence logos from *n* = 2 experimental replicates are shown across the 8 randomized positions.

The differential activity between dgRNA and sgRNA prompted us to ask whether simple spacer sequences or predicted guide RNA structural features could explain or predict guide architecture targeting preferences. We first generated sequence logos from the 255-spacer screen, stratified by architecture preference, but observed no strong nucleotide preferences distinguishing dgRNA from sgRNA activity (Fig. 2A). We then predicted the minimum free energy (MFE) secondary structure of the crRNA (dgRNA) and sgRNA scaffold for each spacer and extracted a panel of structural features using RNAfold and ViennaRNA [47]. Among ten global structural features, none differed significantly across preference groups (Kruskal–Wallis, all *P* >.05) (Fig. 2B and Supplementary Fig. S7). A composite structural score combining the three features most associated with differential editing efficiency (Δ%EE = dgRNA − sgRNA) correlated only weakly with measured Δ%EE across all 255 spacers (Pearson *r* = 0.22, *R*² = 0.05, *P* < .001) (Fig. 2C and Supplementary Fig. S8). Scaffold-disrupting and stem-loop 1(SL1)-complementary spacer motifs in sgRNA, previously linked to reduced sgRNA activity [74–76], as well as repeat-antirepeat pairing in crRNA (dgRNA), were largely absent from this pre-selected, active spacer set and did not distinguish the architecture groups (Fig. 2C–E and Supplementary Fig. S8). Notably, the E2 spacer with the greatest differential in activity between sgRNA and dgRNA for EGFP-targeting guides showed a nearly zero S_weighted score and was neutral in scaffold disruption metrics (Fig. 2B–E).

To experimentally test whether guide architecture itself imposes a sequence preference, we performed *in vitro* cleavage and deep sequencing of products from E4 and E2 guides paired with targets in which the PAM-distal 8 base pairs were randomized (Fig. 2F and G and Supplementary Fig. S9). Attempts to fully randomize the entire 20 nt spacer-target duplex abrogated detectable cleavage (Supplementary Fig. S10). Sequence logos of cleaved products showed only slight differences between architectures, with modest bias at position 8 (Fig. 2F and G and Supplementary Fig. S9). Together, these results indicate that spacer sequence and typical predicted guide RNA structural features are weak predictors of target preference, consistent with prior reports that thermodynamic folding explains only a small fraction of guide RNA activity [77, 78]. They also support engagement of Cas9 with each guide architecture, for example through PAM-proximal spacer interactions or the guide RNA repeat and subsequent target DNA binding as likely sources of differential activity.

### Guide architecture modulates Cas9 conformational dynamics

**Figure 3.**
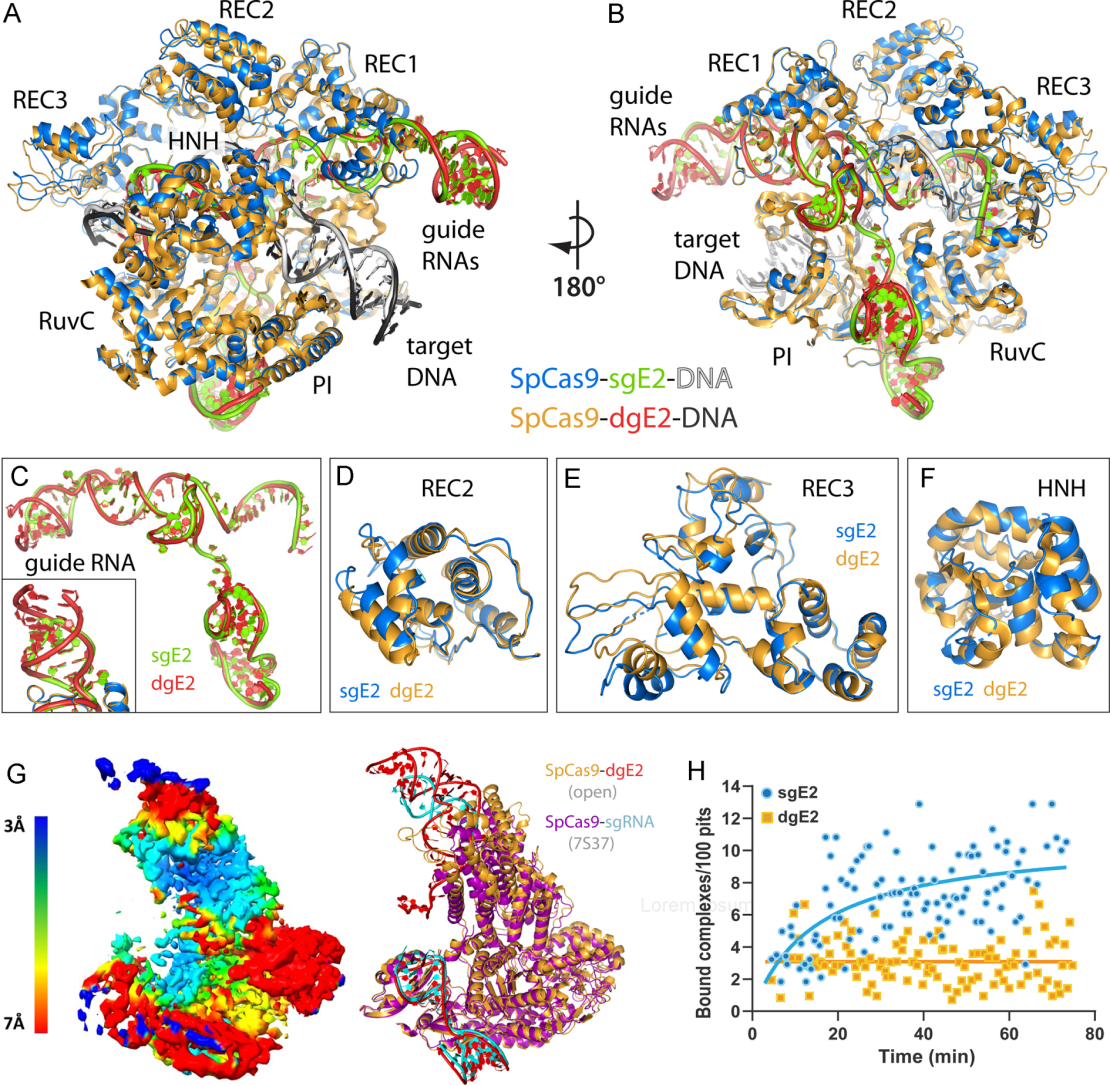
Activity, binding, and cryo-EM reconstructions of Cas9 ternary complexes guided by sgRNA and dgRNA with an E2 spacer. (**A**) Cryo-EM structures of ternary sgE2 (PDB ID: 8G1I) and dgE2 (PDB ID: 8FZT) complexes are shown superimposed and (**B**) rotated 180°. The single and dual guide RNAs are shown in (**C**) with a close-up of the GNRA tetraloop of sgE2 and the open stem of dgE2 shown (inset). The REC2 (**D**), REC3 (**E**), and HNH (**F**) domains from the global superimposition are shown. (**G**) Low-resolution 3D reconstruction of a dgE2 Cas9 ternary open complex color-coded by local resolution of 3–7 angstroms. Fitting of a Cas9 “open” structure previously solved by Doudna and colleagues (PDB ID: 7S37) to the low-resolution structure reconstructed in panel (G) (PDB ID: 9B2K). (**H**) Target dsDNA binding by sgE2 and dgE2 measured by single-molecule convex lens-induced confinement (CLiC) microscopy (n = 2).

To investigate more nuanced conformational changes across guide architectures, we solved matched cryo-EM structures of sgE2 and dgE2 Cas9 ternary complexes. A dgE2 complex was resolved to 3.03 Å, while an sgE2 complex was resolved to 3.12 Å (Supplementary Fig. S11A and B). Refined structures of each complex appear quite similar with a global root mean square deviation (RMSD) of 1.8 Å^2^ (Fig. 3A and B) and both are quite similar to commonly observed “closed” (catalytically competent) conformations [21]. The density corresponding to the repeat terminus of dgE2 was visible for five additional base pairs, as expected (Fig. 3C). Domains with visible shifts between sgE2 and dgE2 were REC2, REC3, and HNH (Fig. 3D–F). These domains are known to act together through conformational checkpoints to sense proper R-loop structure, discriminate against mismatches, and license catalysis [79–83], supporting a role for guide architecture in influencing catalytic activity and substrate specificity. Because shifts of this magnitude can also arise from differences in spacer sequence, we interpret these structural differences cautiously. However, the same spacer sequence was used for both complexes, with the only difference being the guide RNA architecture.

Particles of the sgE2 complex selected for all high-resolution 2D classes yielded a single, well-refined 3D map. However, data for the dgE2 complex yielded an additional prominent high contrast 2D class for nearly half of the particle images (Supplementary Fig. S11C), which corresponded to an alternative class with a map refined to 4.2 Å. Despite attempts to improve resolution with additional data, the best resolution obtained was 3.67 Å. A previously reported “open” conformation structure [84] can be partially fit to this alternative dgE2 complex density map (Fig. 3G), suggesting that about half of the 2D classes observed for dgE2 may be trapped in conformations similar to open, catalytically incompetent states.

To empirically test the formation of trapped alternative states, we selected the E2 spacer for single-molecule binding studies. Using convex lens-induced confinement (CLiC) [85] to track individual binding events between Cas9 RNPs and a plasmid DNA target, we found that sgE2 RNP binding increased over time, while dgE2 RNPs achieved maximum yet incomplete binding within a few minutes, before the start of data collection (Fig. 3H). Thus, dgE2 RNP binding saturated quickly and incompletely while sgE2 RNP binding continued over the course of the experiment, supporting the hypothesis that a fraction of dgE2 RNPs may become trapped in non-productive conformations.

**Figure 4.**
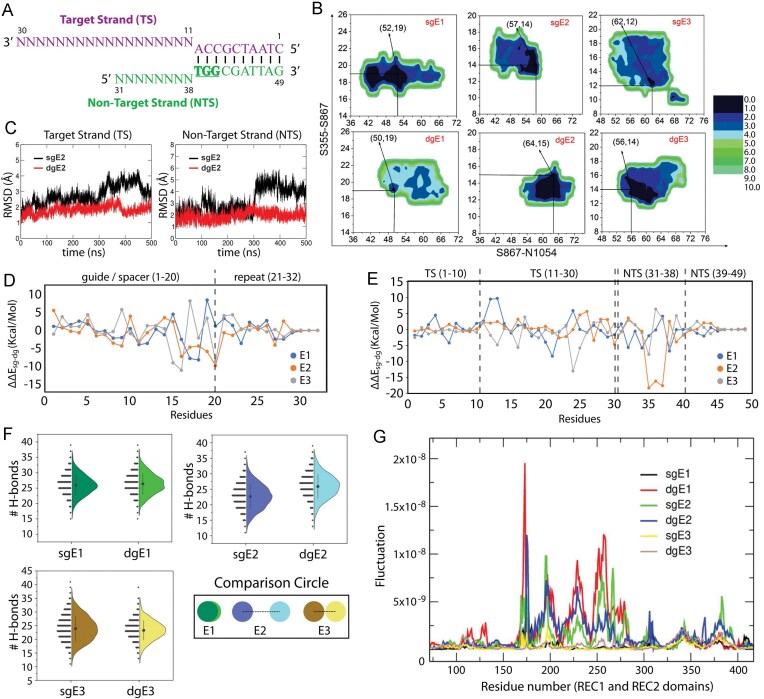
Molecular dynamics simulations identify energetics of TS and NTS DNA, fluctuations in REC domain residues, and global hydrogen bond counts that differ for sgRNA and dgRNA ternary Cas9 complexes. (**A**) Schematic illustration of the TS and NTS and their complementary base pairing used in MD simulation modeling. Numbering convention is continuous from the 5′ end of the TS (nts 1–30) to the 3′ end of the NTS (nts 31–49). (**B**) PMF plots using S867-S355 and S867-N1054 amino acid distances in Cas9 calculated by reweighting 500 ns GaMD trajectories in AMBER 18. The coordinates corresponding to PMF = 0 are indicated by an arrow. (**C**) RMSD graphs of the DNA backbone of the TS (left) and NTS (right) within sgE2- and dgE2-guided Cas9 ternary complexes. RMSD values were calculated from the 500 ns GaMD trajectories. (**D**) Per residue energy decomposition plot of the repeat structure (crRNA:tracrRNA pairing region) of sgRNA and dgRNA in Cas9 ternary complexes. (**E**) Per residue energy decomposition plot of the TS and NTS DNA within the single and dual RNA-guided Cas9 ternary complexes. (**F**) Number of hydrogen bonds between target DNA and Cas9 were calculated in modeled sgRNA and dgRNA Cas9 ternary complexes with E1, E2, and E3 spacer sequences. The values were calculated in VMD using every 100 frames from the 500 ns trajectory. The central point represents the mean value of the histogram distribution. The comparison circle algorithm was used to compare various parameters from the MD simulation trajectories (RMSD of DNA strands, interdomain angles, and H-bond counts) and illustrate the deviation between E1, E2, and E3 sgRNA and dgRNA Cas9 ternary complexes. (**G**) Mobility plot of the REC1 and REC2 domains from visualizing principal component projections in VMD. All calculations were performed using the 500 ns GaMD trajectories.

To gain insight into energetics and dynamics, we performed GaMD simulations with modeled E1, E2, and E3 sgRNA or dgRNA ternary Cas9 complexes [56, 57] (Fig. 4A). We generated PMF plots, which depict low-energy conformation sampling during simulations, using Cα distances between HNH and RuvC amino acids previously reported and characterized by FRET [86] (Fig. 4B). The point of lowest energy (PMF = 0) occurred in the region of 50–65 Å (S867-N1054) and 12–20 Å (S867-S355) (Fig. 4B and Supplementary Fig. S12) as expected [62]. Sampling of distances corresponded with the catalytic state of Cas9 (Supplementary Fig. S12) and the backbone of Cas9 was relatively stable in all complexes based on RMSD calculations (Supplementary Fig. S13). While RMSD values for guide RNA spacers and target strand (TS) and non-target strand (NTS) DNA fluctuated modestly for all complexes (Supplementary Figs S14 and S15), E2 complexes showed significant differences in TS and NTS RMSD at 300 ns (Fig. 4C). We calculated root mean square fluctuation (RMSF) values for R-loop heteroduplexes between spacer RNA and TS, which were consistently low at ∼2 Å (Supplementary Fig. S14D), and the DNA target, which was much higher, up to ∼7 Å for the NTS of dgE2 (nt 31–33) (Supplementary Fig. S16). These results suggest that altered dynamics in the TS and NTS contribute to differential activity of the model dgE2 and sgE2 complexes.

To better understand energetic differences, we performed molecular mechanics-generalized Born surface area (MM/GBSA) analysis. Fluctuations in energy differences for all guide RNAs appeared in the spacer seed region (nt 14–20) and beginning of the repeat structure (nt 21–32) (Fig. 4D). Significant energy fluctuations were also observed in the TS (nt 11–30) and the NTS (nt 31–38) (Fig. 4E). These fluctuations suggest a source of sequence-dependent energetic differences between guide architectures. Violin plot representations of the geometric mean of hydrogen bond interactions between target DNA and Cas9 showed large differences between guide architectures for E2 (Fig. 4F). Using the comparison circle algorithm, which considers H-bonds, interdomain angles, and RMSD of DNA strands to find overall variation, high variation was found between dgE2 and sgE2 complexes (Fig. 4F).

**Figure 5.**
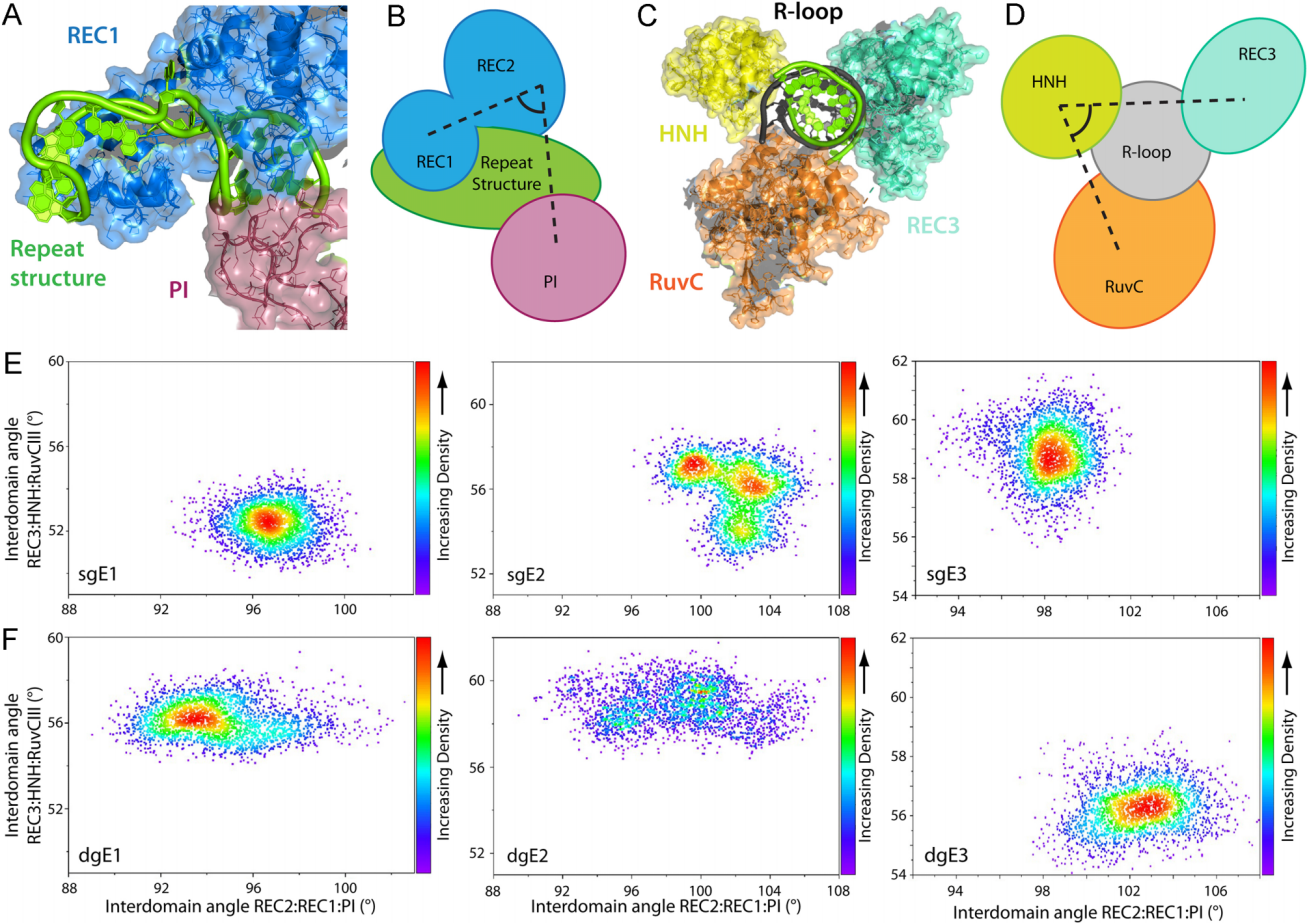
Dynamic REC domain motions differ for sgRNA and dgRNA ternary Cas9 complexes in MD simulations. Structural rendering of the sgE2 Cas9 cryo-EM and illustrations indicating the interdomain angles used to prepare density plots for (**A, B**) the REC1 and PI domains with the repeat structure and (**C, D**) the REC3, RuvC, and HNH domains with the R-loop structure. Density plots between the REC3:HNH:RuvCIII and REC2:REC1:PI domains of (**E**) sgRNA and (**F**) dgRNA for spacers E1, E2, and E3 in Cas9 ternary complexes.

To investigate Cas9 domain dynamics, we performed principal component analysis (PCA) of Cas9 using Cα atoms. PCA showed changes for both guide architectures of the same spacer, suggesting repeat structure-specific effects (Supplementary Fig. S17). The residue mobilities in Principal Component 1 (PC1) indicate that most fluctuations are experienced by the REC1 and REC2 domains (Fig. 4G). We then compared interdomain angles surrounding the repeat structure (REC2:REC1:PI) (Fig. 5A and B) versus the R-loop structure (REC3:HNH:RuvC-II) (Fig. 5C and D). The distribution of angles was more scattered for dgRNAs, indicating more dynamic domain movement (Fig. 5E and F). Changes in the REC2:REC1:PI angles were particularly more pronounced for dgRNA. These suggest that differences in guide architectures may be sensed during early target DNA binding by the PI and REC1/2 domains, which would be transmitted to REC3, HNH, and RuvC domains involved in PAM-distal R-loop structure sensing and catalysis, respectively. Differences in interdomain angle values for E1, E2, and E3 also indicate spacer sequence- and repeat structure-dependent protein dynamics.

### Repeat structure and spacer sequence are codependent

To distinguish the relative contribution of TS or NTS sequences to catalytic activity, we generated synthetic DNA targets where the NTS was varied to that of E1 through E5 or randomized while maintaining the same E2 TS and perfect pairing outside of the 20 nt target sequence (Supplementary Fig. S18A). Cas9 guided by dgE2 consistently cleaved all targets at 70%–80% of sgE2 cleavage irrespective of the variable NTS sequence, which reflected the cleavage of a normal E2 target duplex (Supplementary Fig. S18A). In contrast, using an E1 or E5 TS combined with the E2 NTS resulted in similar cleavage levels for both sgRNA and dgRNA, reflecting the typical cleavage of normal E1 and E5 targets (Supplementary Fig. S18B). These results suggest the TS, which interacts with the spacer to form the R-loop structure, rather than the NTS, plays a greater role in driving differential activity for guide architectures (Supplementary Fig. S18C and D).

### Guide RNA repeat structure contributes to Cas9 specificity

**Figure 6.**
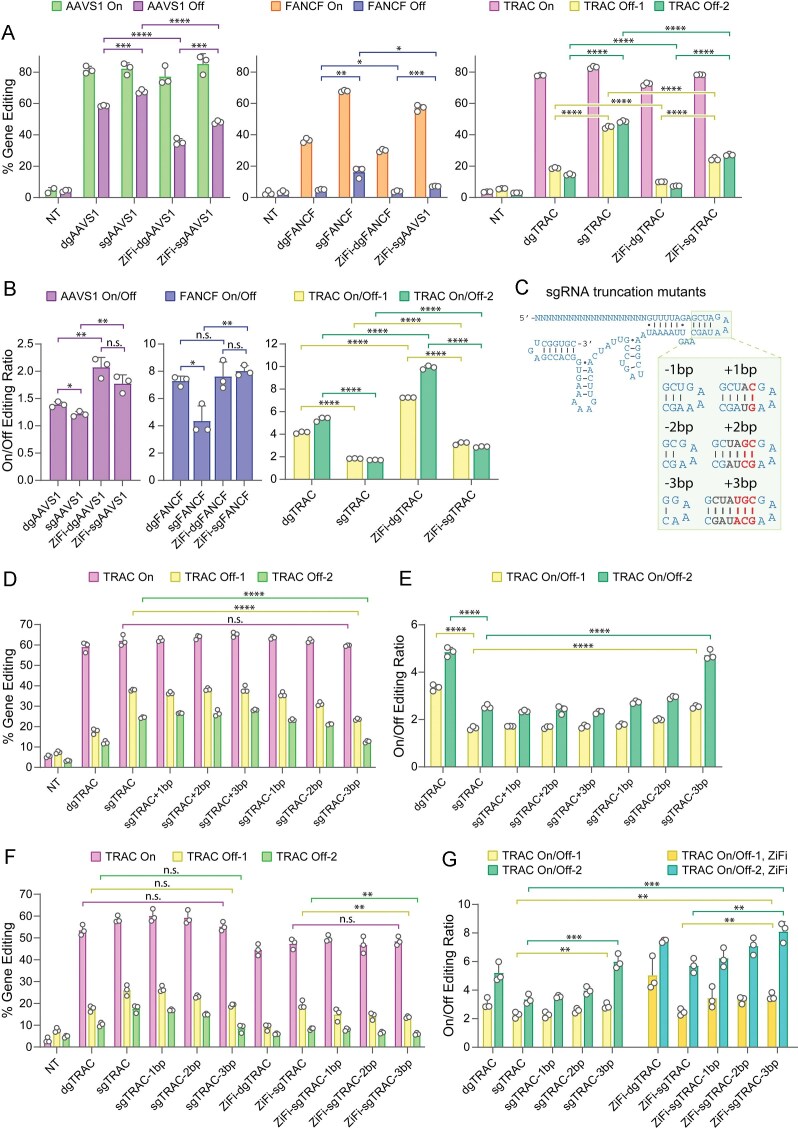
Efficiency and specificity of Cas9 guided by sgRNA, dgRNA, and grtRNA. (**A**) Gene editing at AAVS1, FANCF, and TRAC genes and off-target loci was quantified by nanopore amplicon sequencing and CRISPResso2 (*n* = 3). Dual guide and single guide RNAs were compared with and without ZiFi-Cas9. (**B**) On/off-target editing ratios determined from quantified gene editing data shown in panel (A). (**C**) Illustration of the sequence-level mutations used to create stem mutants of sgTRAC. (**D**–**G**) Gene editing and on/off ratios at the TRAC gene and two off-target sites with sgTRAC stem mutants were quantified by nanopore amplicon sequencing (*n* = 3). For all panels, error is reported as SEM, and unpaired t-test *P*-values are n.s. = not significant, * >.05, ** >.01, *** >.005, ^****^ >.0001. NT, no treatment.

To determine if Cas9 specificity is altered between guide architectures, we targeted three endogenous genes, AAVS1, FANCF, and TRAC-tgt15 (TRAC), which also includes four previously characterized off-target sites in the human genome [82]. dgRNAs offered similar on-target editing as sgRNAs at AAVS1 and TRAC (Fig. 6A), as well as higher specificity profiles (on/off target ratio) at all off-target sites (Fig. 6B). To explore a possible connection with allosteric regulatory mechanisms known to sense R-loop integrity, we combined two key mutations from the LZ3-Cas9 high-fidelity enzyme [87], T769I in the RuvC-HNH linker 1 and G915M in HNH-RuvC linker 2, with the REC3 domain mutation R691A found in HiFi-Cas9 [54]. Average on-target editing with this new mutant, which we refer to as ZiFi-Cas9, was slightly reduced across all guides (Fig. 6A), including E1, E2, and E3 guides (Supplementary Fig. S19A and B), but also provided reductions in off-target editing (Fig. 6A and B). Improvements in on/off target editing by dgRNA were generally additive with ZiFi-Cas9 (Fig. 6B).

To experimentally determine if flexibility or dynamics of the dgRNA repeat structure can modulate off-target editing, we extended or truncated the stem adjacent to the GAAA tetraloop in sgTRAC (Fig. 6C). While adding base pairs had no effect, truncating the stem lowered off-target editing while largely preserving on-target editing, mimicking dgTRAC activity (Fig. 6D). On/off-target editing ratios improved by ~1.3-fold and 1.85-fold when compared to sgRNA for off-target sites 1 and 2, respectively, using sgTRAC-3bp and Cas9 (Fig. 6E). They further improved to ~1.55-fold and 2.4-fold for off-target sites 1 and 2, respectively, when sgTRAC-3bp was paired with ZiFi-Cas9 (Fig. 6F and G). For TRAC gene editing, the 3-bp truncation showed no significant loss of on-target editing (Fig. 6D and F).

### A Cas9 GRC motif regulates catalysis and specificity

**Figure 7.**
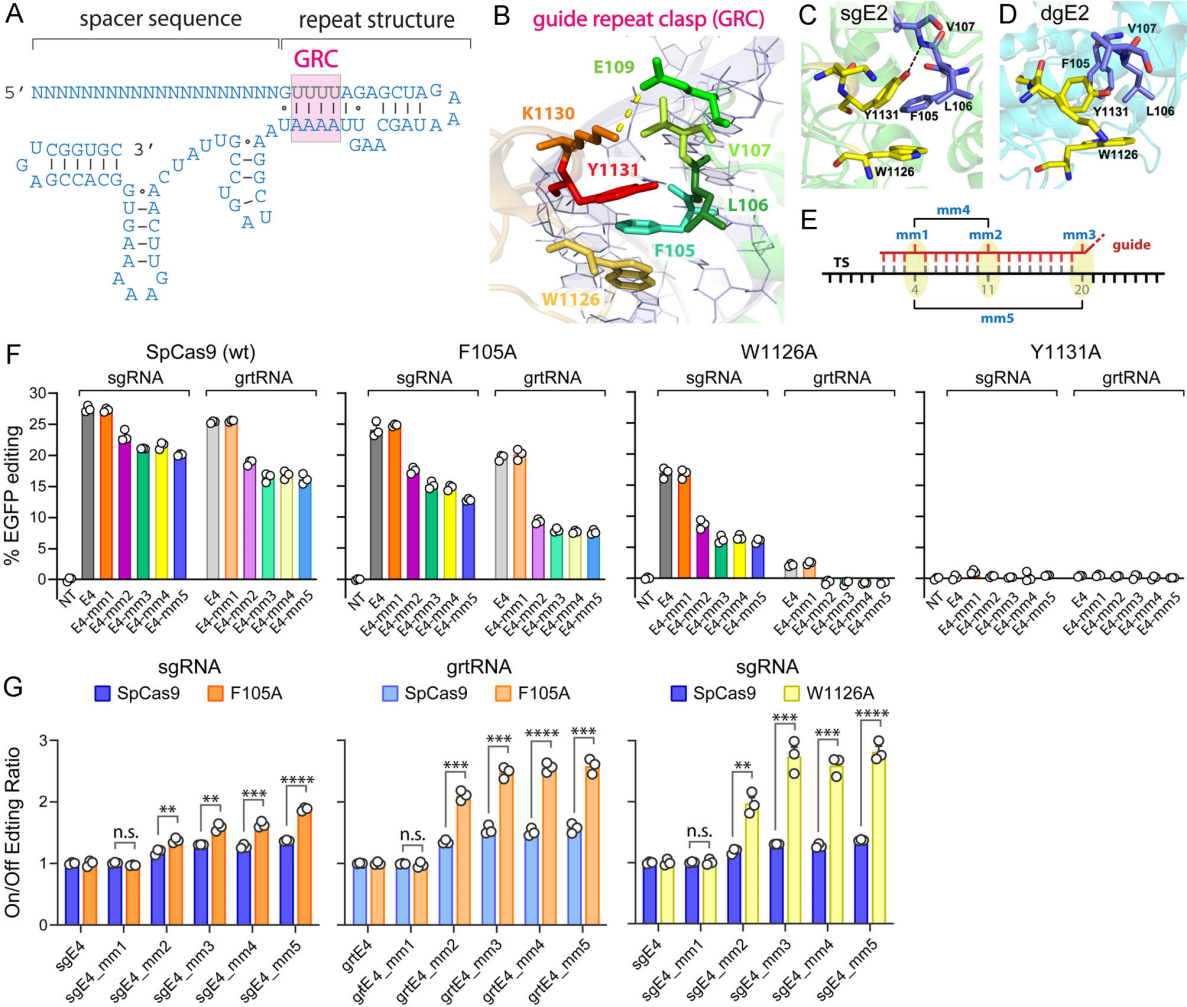
The guide repeat clasp (GRC) motif senses guide RNA structure and controls Cas9 activity. (**A**) Schematic of GRC interaction with the guide RNA repeat structure. (**B**) Close-up of GRC structural organization (from PDB ID: 4UN3). PI domain, REC1 domain, and guide RNA repeat are shown as semi-transparent cartoons in light orange, green, and slate blue, respectively. Guide RNA is shown as lines, while GRC residues are shown as sticks. (**C, D**) GRC amino acid interactions observed in GaMD simulations for sgE2 and dgE2 complexes. (**E**) Illustration of mismatched guide pairing with target DNA for off-target analyses. (**F**) Editing of EGFP with the E4 spacer and mismatch-containing sgRNA or grtRNA and GRC mutants measured by flow cytometry (*n* = 3). (**G**) On/off target editing ratios calculated from panel (F) data using mismatch-containing sgRNAs or grtRNAs with the E4 spacer. Error is reported as SEM. Unpaired t-test *P*-value: n.s. = not significant, * >.05, ** >.01, *** >.005, ^****^ >.0001.

Specificity modulation by TRAC dgRNA or 3-bp guide repeat-truncated sgRNAs, referred to as grtRNAs from here forward, may employ a mechanism that is distinct from, but overlapping with, known R-loop structure-sensing mechanisms exploited by high-fidelity variants, including the ZiFi-Cas9 variant introduced here. Structural inspection revealed that the guide RNA repeat-antirepeat (RAR) duplex appears to act as a nexus for stabilizing interaction between Cas9 REC1 and PI domains (Fig. 7A and B). REC1 residues E103-H116 and PI residues W1126-Y1131 form loops that make substantial direct contact with the guide RAR duplex, including surface complementarity, hydrogen bonding, and charge-charge interactions [21]. REC1 residues F105, L106, V107, and E109 and PI residues W1126, K1130, and Y1131 specifically come together to clasp around the RAR duplex, with E109 and K1130 forming a salt bridge and F105, W1126, and Y1131 interdigitating, supported by L106 and V107, to form a stable hydrophobic pocket (Fig. 7B). In addition, the peptide backbone carbonyl oxygens immediately N-terminal and C-terminal to F105, as well as the backbone carbonyl oxygen between K1130 and Y1131, accept hydrogen bonds from the 2′-hydroxyl groups of conserved uridines U3, U4, and U5 in the guide RNA repeat sequence [21]. These positions correspond with 2′-hydroxyls known to be critical for catalysis through previous chemical probing and guide RNA modification campaigns [30, 32, 39, 41, 50, 88–90]. When we compared low-energy conformers from GaMD simulations for sgE2 and dgE2 complexes, we observed a π–π interaction between Y1131 and F105, a slightly parallel offset π–π interaction between W1126 and F105, and an H-bond between Y1131 and V107 in the sgE2 complex (Fig. 7C). However, in the dgE2 complex, the partial π–π interaction between F105 and W1126 is absent, and the H-bond between Y1131 and V107 is lost (Fig. 7D). Thus, the reduced catalytic activity of dgE2 complexes might be associated with reduced stability or rearrangement of the GRC motif.

To assess the importance of the GRC, we created three individual Cas9 mutants, F105A, W1126A, and Y1131A, which should destabilize the GRC hydrophobic core to varying degrees (Fig. 7B–D). These were paired with single mismatch-containing E4 sgRNAs and grtRNAs that mimic off-target editing at five different positions along the spacer (Fig. 7E and Supplementary Table S1). F105A modestly reduced on-target editing but also reduced off-target editing, resulting in significantly higher on/off-target editing ratios across all mismatched guides except mm1, which bears a single PAM-distal mismatch (Fig. 7F and G). Combining F105A with grtRNA further increased on/off-target editing ratios, albeit at an additional cost of on-target editing (Fig. 7G). W1126A showed a significant decrease in overall activity, losing nearly all editing when paired with grtRNA. However, W1126A improved the on/off target editing ratio significantly at all mismatched positions except mm1 when paired with sgRNAs (Fig. 7G). Both F105A and W1126A mutants had no effect on on/off target editing ratios for PAM-distal mm1, suggesting that the impact on fidelity is limited to more PAM-proximal mismatched targets. In contrast, Y1131A completely abrogated editing (Fig. 7F), presumably by irreversibly compromising GRC stability. Together, these results demonstrate a critical role for the GRC and guide repeat architecture in regulating Cas9 activity.

**Figure 8.**
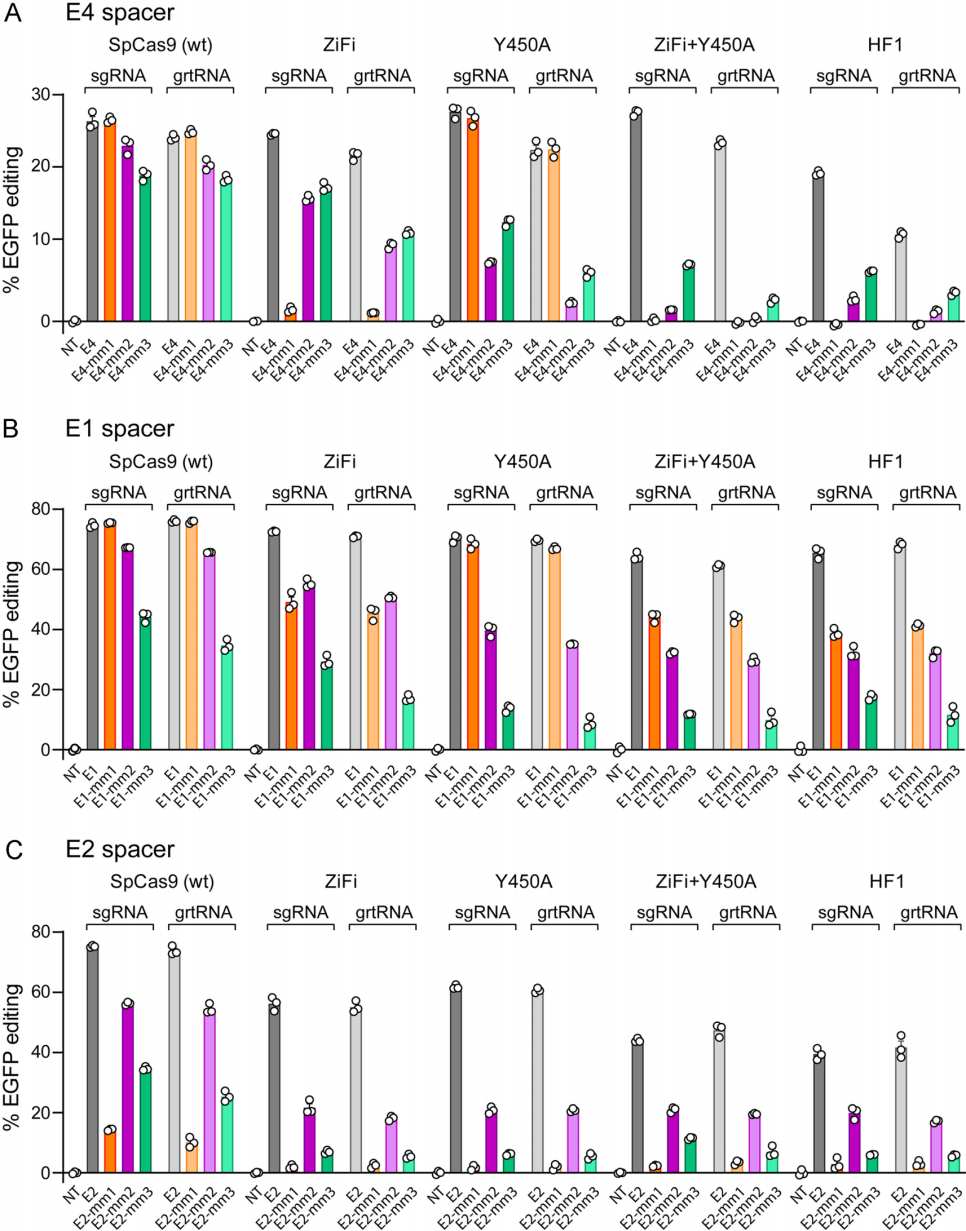
Reduction in off-target editing by ZiFi-Y450A Cas9 and grtRNA is influenced by spacer sequence and mismatch position. (**A**–**C**) Editing of EGFP with the perfect match or mismatch-containing E4, E1, and E2 spacers using sgRNA or grtRNA and SpCas9 (wt), ZiFi, Y450A, ZiFi-Y450A, or HF1 Cas9 variants. % EGFP editing was measured by flow cytometry (*n* = 3). mm1, mm2, and mm3 represent PAM-distal (spacer position 4), PAM-central (spacer position 11), and PAM-proximal mismatches (spacer position 20), respectively. Error bars are reported as SEM.

Synergism between ZiFi-Cas9 and grtRNA suggests that multiple checkpoint mechanisms might be combined to potentially boost Cas9 fidelity. ZiFi mutations were designed to engage the REC domain checkpoint, which primarily senses PAM-distal mismatches [54, 79, 81, 82]. In support of this hypothesis, ZiFi-Cas9 editing with mm1 (PAM-distal) sgRNA was substantially more reduced compared to editing with mm2 (PAM-central) and mm3 (PAM-proximal) sgRNAs with E1, E2, and E4 spacers, especially in the case of E4 (Fig. 8). In contrast, the bipartite seed checkpoint is proposed to sense PAM-central or PAM-proximal mismatches via direct spacer interactions with tyrosine 450 (Y450) [91, 92]. Indeed, a Y450A mutant had little or no effect on mm1 sgRNA editing but substantially reduced mm2 and mm3 sgRNA editing across E1, E2, and E4 spacers (Fig. 8). When ZiFi-Cas9 and Y450A were individually paired with grtRNA, mm2 and mm3 editing was further reduced, while mm1 editing was not for all three spacers (Fig. 8). These results suggest that the effects of grtRNA on editing are translated through the GRC to participate in Cas9 fidelity, with a preference for discriminating against PAM-proximal mismatched targets.

We sought to create a single high-fidelity variant, combining ZiFi and Y450A mutations to make ZiFi-Y450A Cas9, that could pair with grtRNA to achieve better discrimination against a broader diversity of mismatched targets. Remarkably, editing by all five mismatched E4 grtRNAs with ZiFi-Y450A was almost entirely eliminated, while E4 grtRNA on-target editing was maintained at ~90% of normal Cas9 with an E4 sgRNA (Fig. 8A and Supplementary Fig. S21). Combining Y450A with individual or double ZiFi mutations did not identify better combinations (Supplementary Fig. S20), suggesting a synergy between all four of the point mutations used to create ZiFi-Y450A. Editing by HF1, a previously characterized high-fidelity variant, exhibited off-target editing similar to ZiFi-Y450A but with a less favorable reduction in on-target editing (Fig. 8A and Supplementary Fig. S21). Testing these same mismatched guide configurations with E1 and E2 spacers against EGFP also showed reduced off-target editing with grtRNAs and ZiFi, Y450A, ZiFi-Y450A, and HF1 Cas9 variants (Fig. 8B and C, Supplementary Fig. S21). E1 and E2 grtRNAs reduced mm3 (PAM-proximal) editing but not mm1 (PAM-distal) or mm2 (PAM-central), similar to observations for the E4 spacer sequence. grtRNA also supported the same on-target editing as sgRNA for both E1 and E2 spacers across all Cas9 variants. For the E2 spacer, this is significant since grtRNA rescued the substantial loss in editing observed previously when using a dgRNA. However, suppression of off-target editing was not as strong for E1 and E2 spacers, and E2 was particularly sensitive to high-fidelity variants, showing overall larger reductions in on-target editing. While these results demonstrate the potential to combine GRC modulation with other catalytic checkpoint mechanisms to further reduce off-target editing, they also underscore the variability in spacer sequence dependence commonly reported for high-fidelity Cas9 variants [54, 87, 92–94].

**Figure 9.**
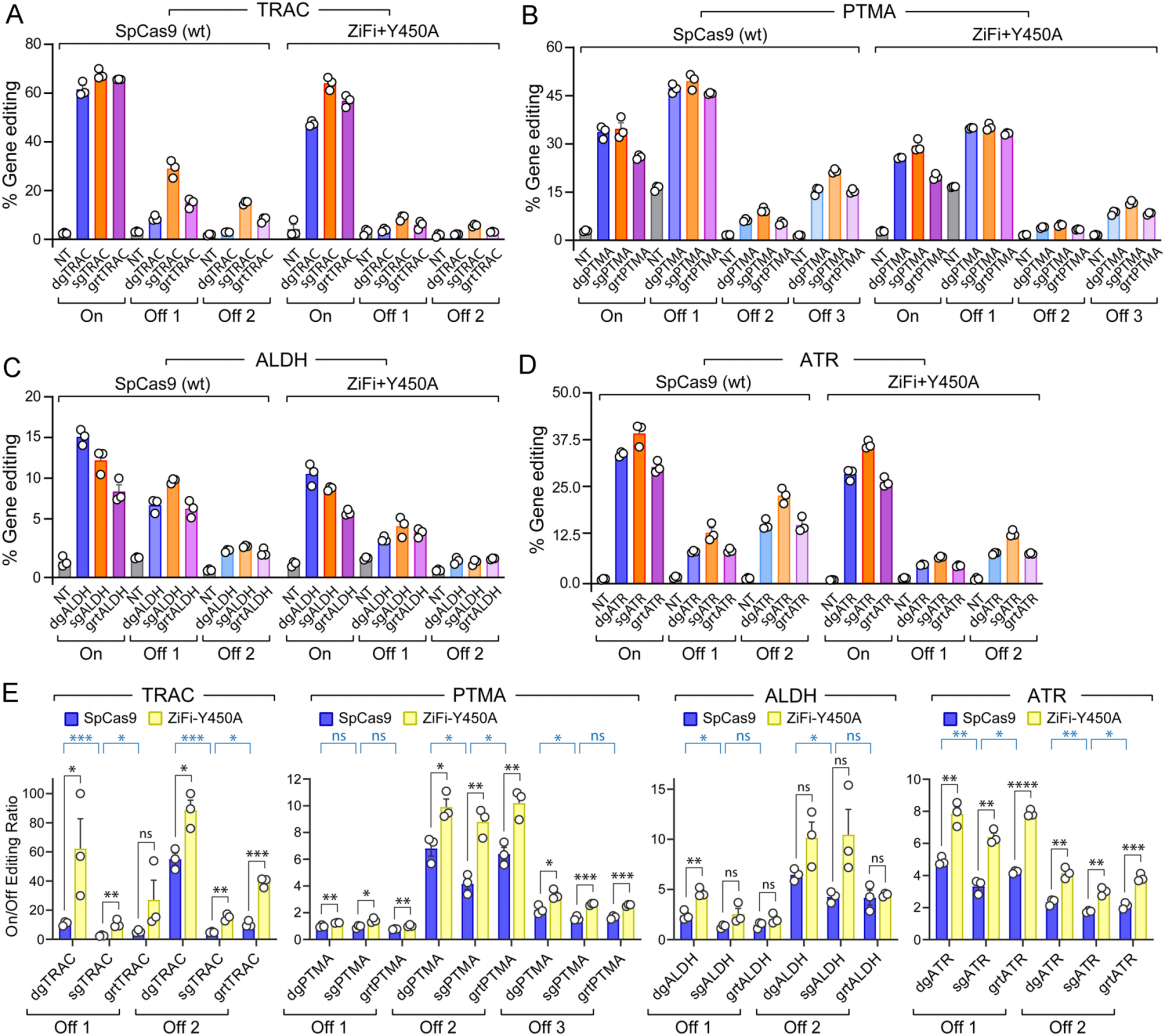
ZiFi-Y450A Cas9, dgRNA, and grtRNA typically improve the on/off-target editing ratio for endogenous loci. (**A**–**D**) Editing of endogenous on- and off-target loci with Cas9 or ZiFi-Y450A and dgRNA, sgRNA, or grtRNA targeting the TRAC, PTMA, ALDH, and ATR genes. Editing was quantified by nanopore amplicon sequencing of on-target and candidate off-target loci and CRISPResso2 analysis (*n* = 3). (**E**) On/off-target editing ratios for data presented in panels (A–D). Error is reported as SEM. Unpaired t-test *P*-value: n.s. = not significant, * >.05, ** >.01, *** >.005, ^****^ >.0001.

To assess on- and off-target editing at endogenous loci, we paired dgRNA, sgRNA, and grtRNA with Cas9 or ZiFi-Y450A and targeted the TRAC, ALDH1A3 (ALDH), ATR, and PTMA genes [87] (Fig. 9A–D). On-target editing was similar for dgRNA and sgRNA across all four genes, while grtRNA was similar to dgRNA for TRAC and ATR but moderately reduced for PTMA and ALDH. However, grtRNA and dgRNA generated similar levels of off-target editing, which was consistently lower than sgRNA for most off-target loci (Fig. 9E). When paired with ZiFi-Y450A, the same trends were observed, with small reductions in on-target editing but greater reductions in off-target editing (Fig. 9). RNP dose and editing duration could influence on/off-target editing, potentially contributing to our observations. However, when we performed TRAC and ALDH editing with 10, 20, or 40 pmols of guide RNA for 24 or 48 h, as opposed to our typical 72 h, we found no statistically significant differences in on/off target editing ratios across doses and only small differences for a few time points (Supplementary Fig. S22). Thus, dgRNA and grtRNA are driving improvements in on/off-target editing by mechanisms distinct from dose and timing effects. In summary, ZiFi-Y450A, which we refer to more simply as ZiFY combined with dgRNA or grtRNA consistently supported lower off-target editing at endogenous loci (Fig. 9E), demonstrating the potential to combine multiple checkpoint mechanisms to achieve higher-fidelity editing by Cas9.

## Discussion

Although Cas9 is well-characterized among CRISPR–Cas enzymes, its regulation and mechanisms when guided by a natural dgRNA have been largely unexplored. Here, while comparing the activity of Cas9 guided by dgRNA and sgRNA, we noted that dgRNA often worked as well or better than sgRNA but performance depended on spacer sequence. Yet spacer sequence and predicted RNA structural features could not explain differential activity. Instead, we found that differential activity between the two guide architectures arose from the guide RNA repeat region and was traced to changes in target DNA and Cas9 structural dynamics, suggesting guide architecture could also influence specificity. Indeed, dgRNA and grtRNA, a hybrid design that conferred partial dgRNA-like repeat structure dynamics onto sgRNA, provided consistent reductions in off-target editing. Improvements in Cas9 fidelity with dgRNA and grtRNA were generally additive with high fidelity Cas9 variants designed to utilize the REC domain and bipartite seed checkpoints. These results support the identification of a previously unappreciated checkpoint mechanism governed by the GRC motif, its interaction with the guide RNA repeat, and its communication with known checkpoint mechanisms. This study now offers a mechanistic explanation for discrepancies in dgRNA versus sgRNA activity and unlocks a potential path toward higher-fidelity enzymes. The GRC has the potential to be tuned, either through mutagenesis of Cas9, modification of guide RNA, or both, and combined with mutations characterized in other high-fidelity variants to further improve Cas9 specificity.

Assembly of Cas9 RNP complexes involves docking of guide RNA onto the Cas9 protein [20, 25]. The correct guide RNA repeat structure creates a favorable interaction nexus, enabling the REC1 and PI domains to physically connect and clasp together around the RNA duplex. The GRC motif is stabilized by a salt bridge, hydrogen bonding, and hydrophobic packing [21] that requires the guide RNA repeat structure and is therefore not resolved or observed in Cas9 apo structures [84]. This clasp interaction is essential for activity, as demonstrated by GRC mutants like Y1131A, and likely serves as a critical RNP assembly checkpoint. However, sensing of repeat structure and dynamics also influences RNP target engagement and catalysis. This effect is best explained by communication with other catalytic checkpoint mechanisms through allostery. Though the GRC clasp is quite distant from the HNH and RuvC catalytic centers, the PI and REC domains are both linked to target DNA engagement through PAM recognition and R-loop interaction, respectively, and are implicated in known allosteric activation mechanisms [25, 62, 81, 82, 95–99]. Conformational shifts in the REC2, REC3, and HNH domains, while modest, were apparent in matched ternary cryo-EM structures, supporting an allosteric link to catalytic efficiency and specificity.

Guide RNA repeat architecture and spacer sequence are codependent, where each appears to influence the other to steer Cas9 catalytic efficiency. This observation also suggests collaboration of the GRC with checkpoint mechanisms that directly interact with the R-loop. The REC domain checkpoint senses R-loop integrity while the bipartite seed checkpoint, which utilizes Y450 in the REC1 domain, surveys PAM-proximal R-loop formation [79, 81, 91]. Both checkpoint mechanisms are known to influence Cas9 specificity and can be modulated with amino acid mutations to increase catalytic fidelity [87, 91–93, 99]. In our study, MD simulations identified dynamics arising from the spacer, especially the seed region, the DNA TS and NTS, and Cas9 REC1 and REC2 domains centered around the PAM-proximal R-loop and repeat structure. And grtRNAs can provide additive improvements in specificity when paired with mutations that act through the REC domain checkpoint (ZiFi-Cas9) and bipartite seed checkpoint (Y450A-Cas9), suggesting distinct but interdependent mechanisms.

Altered dynamics can change the energetic landscape of Cas9 conformational transitions required to engage and cleave target DNA [100, 101], which can induce lower off-target editing by partially inhibiting allostery or catalysis [98, 99, 102]. Many high-fidelity Cas9 variants function by mildly reducing target affinity or altering allosteric networks. For example, the Cas9 HF1 variant was rationally designed to reduce the dwell time of Cas9 binding on target substrates by reducing charge–charge interactions with the R-loop structure [92]. Mutations of variants selected by directed evolution are predicted to function by similar mechanisms [93]. Unfortunately, these mutations often come at the cost of reduced on-target editing efficiency and there appears to be a limit to the degree of fidelity achievable. Nonetheless, combining multiple independent mechanisms might help maintain on-target editing while further minimizing off-target editing [87, 98].

A more flexible or less stable repeat region, such as with dgRNA or grtRNA, can increase or decrease cleavage and editing efficiency depending on the spacer sequence. These results would suggest an intrinsic sequence preference for dgRNA versus sgRNA, which would enable better predictability for guide architecture selection. However, sequence logo generation and partial randomization of the PAM-distal R-loop followed by sequencing for two separate spacer sequences did not reveal consequential sequence preferences between sgRNA and dgRNA. Likewise, guide RNA structure predictions did not reveal strong correlations between spacer sequence, predicted structural features, and sgRNA or dgRNA editing preferences. Together, these results suggest that preferences may lie predominantly in the PAM-proximal spacer region and be embedded in crosstalk between Cas9 checkpoint and GRC mechanisms. Swapping non-complementary NTS sequences, combined with MD studies, also supports a role for the PAM-proximal R-loop in coordinating with the GRC to dictate Cas9 activity and specificity. We found that dgRNA and grtRNA usually conferred lower off-target editing, especially for PAM-proximal mismatched targets, though the magnitude of this effect was spacer-dependent. Unfortunately, spacer sequence-dependent effects remain a significant challenge in creating universal, high-fidelity Cas9 enzymes with high on-target but low off-target editing [85, 86, 88–90].

A commonly held assertion has been that dgRNAs do not perform as well as sgRNAs. However, this study establishes that dgRNAs can sometimes be superior in efficiency and specificity, making a compelling case to include dgRNAs, and now grtRNA designs, in more mechanistic investigations and when optimizing on/off target editing for specific target sequences. While only a limited number of spacer sequences could be investigated here, we found that one spacer sequence, E2, showed substantial differential activity between sgRNA and dgRNA, nominating it as a model for detailed analyses. E2 provided an informative example. However, it is unclear to what extent structural and functional results will translate broadly to other spacer sequences. To fully explore the potential for the GRC to improve fidelity, deep mutagenesis or directed evolution techniques may be required for both the guide RNA repeat region and Cas9 protein. Combinations of GRC mutants and guide repeat modifications should enable even higher fidelity Cas9 enzymes when paired with other mutations, as demonstrated with our rationally designed ZiFY variant.

Roles for the guide RNA repeat structure and the GRC in Cas9 fidelity have not been previously reported. Amino acids involved in formation of the GRC motif have not appeared in other high-fidelity Cas9 variants [87, 93], and the guide RNA repeat sequence and structure have remained unchanged since the earliest published CRISPR–Cas9 studies [20, 25]. The grtRNA design introduced here is distinct from previously reported truncated guide RNAs [103–105], where the 5′ end of the spacer is simply shortened, as these modulate R-loop structure/stability sensing through REC domain checkpoint mechanisms [105]. A recent example of 5′-truncated guide RNAs that enhance single-base mismatch discrimination further supports this distinction [106].

The principles uncovered here provide a framework for developing new, more specific CRISPR–Cas tools and safer therapeutics. These include focused mechanistic studies on the effects of repeat structure on checkpoint mechanisms, optimization of guide repeat structures, and combining grtRNAs and GRC mutants with other high-fidelity strategies, like existing Cas9 variants [87, 93] or 5′ truncated spacers [103, 105]. Our findings may also translate to other Cas9-based platforms, like base editing or prime editing where binding specificity is paramount or where sgRNAs are necessary but might now be replaced with grtRNAs [8, 12, 13]. Chemical modifications in the guide repeat region are expected to alter stability and dynamics and could therefore impact Cas9 fidelity. Thus, guide RNA chemical modification campaigns should consider the impact on Cas9 specificity [41]. The grtRNA design also offers a more compact sgRNA and the potential for further optimization. Finally, the phenomenon of an RNP assembly checkpoint, where binding to a unique RNA structure or sequence is prerequisite for catalysis, suggests the potential for mechanisms like the GRC to impact efficiency and specificity broadly across other RNA-guided enzymes and CRISPR–Cas systems.

## Supplementary Material

gkag710_Supplemental_Files

## Data Availability

All data supporting the findings of this study are available within the paper and its Extended Data. Cryo-EM structures have been deposited in the RCSB Protein Databank with DOIs https://doi.org/10.2210/pdb8g1t/pdb, https://doi.org/10.2210/pdb8fzt/pdb, and https://doi.org/10.2210/pdb9b2k/pdb.

## References

[1] Cong L, Ran FA, Cox D et al. Multiplex genome engineering using CRISPR/Cas systems. Science. 2013;339:819–23. 10.1126/science.1231143. 23287718 PMC3795411

[2] Mali P, Yang L, Esvelt KM et al. RNA-guided human genome engineering via Cas9. Science. 2013;339:823–6. 10.1126/science.1232033. 23287722 PMC3712628

[3] Sander JD, Joung JK. CRISPR–Cas systems for editing, regulating and targeting genomes. Nat Biotechnol. 2014;32:347–55. 10.1038/nbt.2842. 24584096 PMC4022601

[4] Hilton IB, D’Ippolito AM, Vockley CM et al. Epigenome editing by a CRISPR–Cas9-based acetyltransferase activates genes from promoters and enhancers. Nat Biotechnol. 2015;33:510–7. 10.1038/nbt.3199. 25849900 PMC4430400

[5] Kennedy EM, Kornepati AV, Cullen BR. Targeting hepatitis B virus cccDNA using CRISPR/Cas9. Antiviral Res. 2015;123:188–92. 10.1016/j.antiviral.2015.10.004. 26476375

[6] Dai WJ, Zhu LY, Yan ZY et al. CRISPR–Cas9 for *in vivo* gene therapy: promise and hurdles. Mol Ther Nucleic Acids. 2016;5:e349. 10.1038/mtna.2016.58. 28131272 PMC5023403

[7] Du D, Qi LS. CRISPR technology for genome activation and repression in mammalian cells. Cold Spring Harb Protoc. 2016;2016:pdb.prot090175. 10.1101/pdb.prot090175. 26729910

[8] Komor AC, Kim YB, Packer MS et al. Programmable editing of a target base in genomic DNA without double-stranded DNA cleavage. Nature. 2016;533:420–4. 10.1038/nature17946. 27096365 PMC4873371

[9] Vojta A, Dobrinic P, Tadic V et al. Repurposing the CRISPR–Cas9 system for targeted DNA methylation. Nucleic Acids Res. 2016;44:5615–28. 10.1093/nar/gkw159. 26969735 PMC4937303

[10] Maji B, Moore CL, Zetsche B et al. Multidimensional chemical control of CRISPR–Cas9. Nat Chem Biol. 2017;13:9–11. 10.1038/nchembio.2224. 27820801 PMC5531067

[11] Gaudelli NM, Komor AC, Rees HA et al. Programmable base editing of A*T to G*C in genomic DNA without DNA cleavage. Nature. 2017;551:464–71. 10.1038/nature24644. 29160308 PMC5726555

[12] Anzalone AV, Randolph PB, Davis JR et al. Search-and-replace genome editing without double-strand breaks or donor DNA. Nature. 2019;576:149–57. 10.1038/s41586-019-1711-4. 31634902 PMC6907074

[13] Anzalone AV, Koblan LW, Liu DR. Genome editing with CRISPR–Cas nucleases, base editors, transposases and prime editors. Nat Biotechnol. 2020;38:824–44. 10.1038/s41587-020-0561-9. 32572269

[14] Yarnall MTN, Ioannidi EI, Schmitt-Ulms C et al. Drag-and-drop genome insertion of large sequences without double-strand DNA cleavage using CRISPR-directed integrases. Nat Biotechnol. 2023;41:500–12. 10.1038/s41587-022-01527-4. 36424489 PMC10257351

[15] Makarova KS, Grishin NV, Shabalina SA et al. A putative RNA-interference-based immune system in prokaryotes: computational analysis of the predicted enzymatic machinery, functional analogies with eukaryotic RNAi, and hypothetical mechanisms of action. Biol Direct. 2006;1:7. 10.1186/1745-6150-1-7. 16545108 PMC1462988

[16] Barrangou R, Fremaux C, Deveau H et al. CRISPR provides acquired resistance against viruses in prokaryotes. Science. 2007;315:1709–12. 10.1126/science.1138140. 17379808

[17] Bhaya D, Davison M, Barrangou R. CRISPR–Cas systems in bacteria and archaea: versatile small RNAs for adaptive defense and regulation. Annu Rev Genet. 2011;45:273–97. 10.1146/annurev-genet-110410-132430. 22060043

[18] Briner AE, Donohoue PD, Gomaa AA et al. Guide RNA functional modules direct Cas9 activity and orthogonality. Mol Cell. 2014;56:333–9. 10.1016/j.molcel.2014.09.019. 25373540

[19] Chylinski K, Makarova KS, Charpentier E et al. Classification and evolution of type II CRISPR–Cas systems. Nucleic Acids Res. 2014;42:6091–105. 10.1093/nar/gku241. 24728998 PMC4041416

[20] Jinek M, Chylinski K, Fonfara I et al. A programmable dual-RNA-guided DNA endonuclease in adaptive bacterial immunity. Science. 2012;337:816–21. 10.1126/science.1225829. 22745249 PMC6286148

[21] Nishimasu H, Ran FA, Hsu PD et al. Crystal structure of Cas9 in complex with guide RNA and target DNA. Cell. 2014;156:935–49. 10.1016/j.cell.2014.02.001. 24529477 PMC4139937

[22] Hendel A, Bak RO, Clark JT et al. Chemically modified guide RNAs enhance CRISPR–Cas genome editing in human primary cells. Nat Biotechnol. 2015;33:985–9. 10.1038/nbt.3290. 26121415 PMC4729442

[23] Doench JG, Fusi N, Sullender M et al. Optimized sgRNA design to maximize activity and minimize off-target effects of CRISPR–Cas9. Nat Biotechnol. 2016;34:184–91. 10.1038/nbt.3437. 26780180 PMC4744125

[24] Ma J, Koster J, Qin Q et al. CRISPR-DO for genome-wide CRISPR design and optimization. Bioinformatics. 2016;32:3336–8. 27402906 10.1093/bioinformatics/btw476 PMC6095119

[25] Jiang F, Doudna JA. CRISPR–Cas9 structures and mechanisms. Annu Rev Biophys. 2017;46:505–29. 10.1146/annurev-biophys-062215-010822. 28375731

[26] Taemaitree L, Shivalingam A, El-Sagheer AH et al. “Split-and-click” sgRNA. Methods Mol Biol. 2021;2162:61–78. 32926378 10.1007/978-1-0716-0687-2_5

[27] Chen Z, Devi G, Arif A et al. Tetrazine-ligated CRISPR sgRNAs for efficient genome editing. ACS Chem Biol. 2022;17:1045–50. 10.1021/acschembio.2c00116. 35446558 PMC9127786

[28] Fonfara I, Le Rhun A, Chylinski K et al. Phylogeny of Cas9 determines functional exchangeability of dual-RNA and Cas9 among orthologous type II CRISPR–Cas systems. Nucleic Acids Res. 2014;42:2577–90. 10.1093/nar/gkt1074. 24270795 PMC3936727

[29] Rahdar M, McMahon MA, Prakash TP et al. Synthetic CRISPR RNA-Cas9-guided genome editing in human cells. Proc Natl Acad Sci USA. 2015;112:E7110–7. 26589814 10.1073/pnas.1520883112 PMC4697396

[30] Mir A, Alterman JF, Hassler MR et al. Heavily and fully modified RNAs guide efficient SpyCas9-mediated genome editing. Nat Commun. 2018;9:2641. 10.1038/s41467-018-05073-z. 29980686 PMC6035171

[31] Schubert MS, Cedrone E, Neun B et al. Chemical modification of CRISPR gRNAs eliminate type I interferon responses in human peripheral blood mononuclear cells. J Cytokine Biol. 2018;3:121. 30225466 10.4172/2576-3881.1000121 PMC6138052

[32] O’Reilly D, Kartje ZJ, Ageely EA et al. Extensive CRISPR RNA modification reveals chemical compatibility and structure-activity relationships for Cas9 biochemical activity. Nucleic Acids Res. 2019;47:546–58. 30517736 10.1093/nar/gky1214 PMC6344873

[33] Shapiro J, Iancu O, Jacobi AM et al. Increasing CRISPR efficiency and measuring its specificity in HSPCs using a clinically relevant system. Mol Ther Methods Clin Dev. 2020;17:1097–107. 10.1016/j.omtm.2020.04.027. 32478125 PMC7251314

[34] Scott T, Urak R, Soemardy C et al. Improved Cas9 activity by specific modifications of the tracrRNA. Sci Rep. 2019;9:16104. 10.1038/s41598-019-52616-5. 31695072 PMC6834579

[35] Okada K, Aoki K, Tabei T et al. Key sequence features of CRISPR RNA for dual-guide CRISPR–Cas9 ribonucleoprotein complexes assembled with wild-type or HiFi Cas9. Nucleic Acids Res. 2022;50:2854–71. 10.1093/nar/gkac100. 35166844 PMC8934663

[36] O’Geen H, Henry IM, Bhakta MS et al. A genome-wide analysis of Cas9 binding specificity using ChIP-seq and targeted sequence capture. Nucleic Acids Res. 2015;43:3389–404. 10.1093/nar/gkv137. 25712100 PMC4381059

[37] Sanson KR, Hanna RE, Hegde M et al. Optimized libraries for CRISPR–Cas9 genetic screens with multiple modalities. Nat Commun. 2018;9:5416. 10.1038/s41467-018-07901-8. 30575746 PMC6303322

[38] Chen J, Chen Y, Huang L et al. Trans-nuclease activity of Cas9 activated by DNA or RNA target binding. Nat Biotechnol. 2025;43:558–68. 38811761 10.1038/s41587-024-02255-7

[39] Yin H, Song CQ, Suresh S et al. Structure-guided chemical modification of guide RNA enables potent non-viral *in vivo* genome editing. Nat Biotechnol. 2017;35:1179–87. 10.1038/nbt.4005. 29131148 PMC5901668

[40] Zhang H, Kelly K, Lee J et al. Self-delivering, chemically modified CRISPR RNAs for AAV co-delivery and genome editing *in vivo*. Nucleic Acids Res. 2023;52:977–97. 10.1093/nar/gkad1125 PMC10810193 38033325

[41] Barber HM, Pater AA, Gagnon KT et al. Chemical engineering of CRISPR guide RNAs for therapeutic application. Nat Rev Drug Discov. 2025;24:209–30. 39690326 10.1038/s41573-024-01086-0 PMC13347398

[42] Damha MJ, Ogilvie KK. Oligoribonucleotide synthesis. The silyl-phosphoramidite method. Methods Mol Biol. 1993;20:81–114. 8242149 10.1385/0-89603-281-7:81

[43] Carriero S, Damha MJ. Inhibition of pre-mRNA splicing by synthetic branched nucleic acids. Nucleic Acids Res. 2003;31:6157–67. 10.1093/nar/gkg824. 14576302 PMC275466

[44] Zuris JA, Thompson DB, Shu Y et al. Cationic lipid-mediated delivery of proteins enables efficient protein-based genome editing *in vitro* and *in vivo*. Nat Biotechnol. 2015;33:73–80. 10.1038/nbt.3081. 25357182 PMC4289409

[45] Anders C, Jinek M. *In vitro* enzymology of Cas9. Methods Enzymol. 2014;546:1–20. 25398333 10.1016/B978-0-12-801185-0.00001-5 PMC5074358

[46] Anders C, Niewoehner O, Duerst A et al. Structural basis of PAM-dependent target DNA recognition by the Cas9 endonuclease. Nature. 2014;513:569–73. 10.1038/nature13579. 25079318 PMC4176945

[47] Lorenz R, Bernhart SH, Honer Zu Siederdissen C et al. ViennaRNA package 2.0. Algorithms Mol Biol. 2011;6:26. 22115189 10.1186/1748-7188-6-26 PMC3319429

[48] Mathews DH, Disney MD, Childs JL et al. Incorporating chemical modification constraints into a dynamic programming algorithm for prediction of RNA secondary structure. Proc Natl Acad Sci USA. 2004;101:7287–92. 10.1073/pnas.0401799101. 15123812 PMC409911

[49] Crooks GE, Hon G, Chandonia JM et al. WebLogo: a sequence logo generator. Genome Res. 2004;14:1188–90. 10.1101/gr.849004. 15173120 PMC419797

[50] Kartje ZJ, Barkau CL, Rohilla KJ et al. Chimeric guides probe and enhance Cas9 biochemical activity. Biochemistry. 2018;57:3027–31. 10.1021/acs.biochem.8b00107. 29746102

[51] Martin-Pintado N, Yahyaee-Anzahaee M, Campos-Olivas R et al. The solution structure of double helical arabino nucleic acids (ANA and 2′F-ANA): effect of arabinoses in duplex-hairpin interconversion. Nucleic Acids Res. 2012;40:9329–39. 10.1093/nar/gks672. 22798499 PMC3467067

[52] Barkau CL, O’Reilly D, Rohilla KJ et al. Rationally designed anti-CRISPR nucleic acid inhibitors of CRISPR–Cas9. Nucleic Acid Ther. 2019;29:136–47. 10.1089/nat.2018.0758. 30990769 PMC6555185

[53] Scott S, Xu ZM, Kouzine F et al. Visualizing structure-mediated interactions in supercoiled DNA molecules. Nucleic Acids Res. 2018;46:4622–31. 10.1093/nar/gky266. 29684182 PMC5961182

[54] Vakulskas CA, Dever DP, Rettig GR et al. A high-fidelity Cas9 mutant delivered as a ribonucleoprotein complex enables efficient gene editing in human hematopoietic stem and progenitor cells. Nat Med. 2018;24:1216–24. 10.1038/s41591-018-0137-0. 30082871 PMC6107069

[55] Sali A, Blundell TL. Comparative protein modelling by satisfaction of spatial restraints. J Mol Biol. 1993;234:779–815. 10.1006/jmbi.1993.1626. 8254673

[56] Jiang F, Taylor DW, Chen JS et al. Structures of a CRISPR–Cas9 R-loop complex primed for DNA cleavage. Science. 2016;351:867–71. 10.1126/science.aad8282. 26841432 PMC5111852

[57] Huai C, Li G, Yao R et al. Structural insights into DNA cleavage activation of CRISPR–Cas9 system. Nat Commun. 2017;8:1375. 10.1038/s41467-017-01496-2. 29123204 PMC5680257

[58] Pettersen EF, Goddard TD, Huang CC et al. UCSF Chimera—a visualization system for exploratory research and analysis. J Comput Chem. 2004;25:1605–12. 10.1002/jcc.20084. 15264254

[59] Maier JA, Martinez C, Kasavajhala K et al. ff14SB: improving the accuracy of protein side chain and backbone parameters from ff99SB. J Chem Theory Comput. 2015;11:3696–713. 10.1021/acs.jctc.5b00255. 26574453 PMC4821407

[60] Ivani I, Dans PD, Noy A et al. Parmbsc1: a refined force field for DNA simulations. Nat Methods. 2016;13:55–8. 10.1038/nmeth.3658. 26569599 PMC4700514

[61] Zgarbova M, Otyepka M, Sponer J et al. Refinement of the Cornell et al. Nucleic acids force field based on reference quantum chemical calculations of glycosidic torsion profiles. J Chem Theory Comput. 2011;7:2886–902. 10.1021/ct200162x. 21921995 PMC3171997

[62] Palermo G, Miao Y, Walker RC et al. CRISPR–Cas9 conformational activation as elucidated from enhanced molecular simulations. Proc Natl Acad Sci USA. 2017;114:7260–5. 10.1073/pnas.1707645114. 28652374 PMC5514767

[63] Salomon-Ferrer R, Gotz AW, Poole D et al. Routine microsecond molecular dynamics simulations with AMBER on GPUs. 2. Explicit solvent particle mesh Ewald. J Chem Theory Comput. 2013;9:3878–88. 10.1021/ct400314y. 26592383

[64] Gotz AW, Williamson MJ, Xu D et al. Routine microsecond molecular dynamics simulations with AMBER on GPUs. 1. Generalized born. J Chem Theory Comput. 2012;8:1542–55. 22582031 10.1021/ct200909j PMC3348677

[65] Case DA, Aktulga HM, Belfon K et al. AmberTools. J Chem Inf Model. 2023;63:6183–91. 10.1021/acs.jcim.3c01153. 37805934 PMC10598796

[66] Miao Y, Feher VA, McCammon JA. Gaussian accelerated molecular dynamics: unconstrained enhanced sampling and free energy calculation. J Chem Theory Comput. 2015;11:3584–95. 10.1021/acs.jctc.5b00436. 26300708 PMC4535365

[67] Roe DR, Cheatham TE 3rd. PTRAJ and CPPTRAJ: software for processing and analysis of molecular dynamics trajectory data. J Chem Theory Comput. 2013;9:3084–95. 10.1021/ct400341p. 26583988

[68] Humphrey W, Dalke A, Schulten K. VMD: visual molecular dynamics. J Mol Graphics. 1996;14:33–8. 10.1016/0263-7855(96)00018-5. 8744570

[69] Miao Y, Sinko W, Pierce L et al. Improved reweighting of accelerated molecular dynamics simulations for free energy calculation. J Chem Theory Comput. 2014;10:2677–89. 10.1021/ct500090q. 25061441 PMC4095935

[70] Kollman PA, Massova I, Reyes C et al. Calculating structures and free energies of complex molecules: combining molecular mechanics and continuum models. Acc Chem Res. 2000;33:889–97. 10.1021/ar000033j. 11123888

[71] Sudhakar S, Barkau CL, Chilamkurthy R et al. Binding to the conserved and stably folded guide RNA pseudoknot induces Cas12a conformational changes during ribonucleoprotein assembly. J Biol Chem. 2023;299:104700. 10.1016/j.jbc.2023.104700. 37059184 PMC10200996

[72] Jiang F, Zhou K, Ma L et al. A Cas9-guide RNA complex preorganized for target DNA recognition. Science. 2015;348:1477–81. 10.1126/science.aab1452. 26113724

[73] Koshkin AA, Nielsen P, Meldgaard M et al. LNA (locked nucleic acid): an RNA mimic forming exceedingly stable LNA:LNA duplexes. J Am Chem Soc. 1998;120:13252–3. 10.1021/ja9822862.

[74] Graf R, Li X, Chu VT et al. sgRNA sequence motifs blocking efficient CRISPR/Cas9-mediated gene editing. Cell Rep. 2019;26:1098–1103. 10.1016/j.celrep.2019.01.024. 30699341 PMC6352712

[75] Huszar K, Welker Z, Gyorgypal Z et al. Position-dependent sequence motif preferences of SpCas9 are largely determined by scaffold-complementary spacer motifs. Nucleic Acids Res. 2023;51:5847–63. 10.1093/nar/gkad323. 37140059 PMC10287927

[76] Thyme SB, Akhmetova L, Montague TG et al. Internal guide RNA interactions interfere with Cas9-mediated cleavage. Nat Commun. 2016;7:11750. 10.1038/ncomms11750. 27282953 PMC4906408

[77] Labuhn M, Adams FF, Ng M et al. Refined sgRNA efficacy prediction improves large- and small-scale CRISPR–Cas9 applications. Nucleic Acids Res. 2018;46:1375–85. 10.1093/nar/gkx1268. 29267886 PMC5814880

[78] Liang Z, Huang C, Xia Y et al. Identification of functional sgRNA mutants lacking canonical secondary structure using high-throughput FACS screening. Cell Rep. 2024;43:114290. 10.1016/j.celrep.2024.114290. 38823012

[79] Chen JS, Dagdas YS, Kleinstiver BP et al. Enhanced proofreading governs CRISPR–Cas9 targeting accuracy. Nature. 2017;550:407–10. 10.1038/nature24268. 28931002 PMC5918688

[80] Sung K, Park J, Kim Y et al. Target specificity of Cas9 nuclease via DNA rearrangement regulated by the REC2 domain. J Am Chem Soc. 2018;140:7778–81. 10.1021/jacs.8b03102. 29874063

[81] Dagdas YS, Chen JS, Sternberg SH et al. A conformational checkpoint between DNA binding and cleavage by CRISPR–Cas9. Sci Adv. 2017;3:eaao0027. 10.1126/sciadv.aao0027. 28808686 PMC5547770

[82] Pacesa M, Lin CH, Clery A et al. Structural basis for Cas9 off-target activity. Cell. 2022;185:4067–81. 10.1016/j.cell.2022.09.026. 36306733 PMC10103147

[83] Bravo JPK, Liu MS, Hibshman GN et al. Structural basis for mismatch surveillance by CRISPR–Cas9. Nature. 2022;603:343–7. 10.1038/s41586-022-04470-1. 35236982 PMC8907077

[84] Jinek M, Jiang F, Taylor DW et al. Structures of Cas9 endonucleases reveal RNA-mediated conformational activation. Science. 2014;343:1247997. 10.1126/science.1247997. 24505130 PMC4184034

[85] Leslie SR, Fields AP, Cohen AE. Convex lens-induced confinement for imaging single molecules. Anal Chem. 2010;82:6224–9. 10.1021/ac101041s. 20557026 PMC3172322

[86] Sternberg SH, LaFrance B, Kaplan M et al. Conformational control of DNA target cleavage by CRISPR–Cas9. Nature. 2015;527:110–3. 10.1038/nature15544. 26524520 PMC4859810

[87] Schmid-Burgk JL, Gao L, Li D et al. Highly parallel profiling of Cas9 variant specificity. Mol Cell. 2020;78:794–800. 10.1016/j.molcel.2020.02.023. 32187529 PMC7370240

[88] Pater AA, Barber HM, Sudhakar S et al. Chemical control of 2′-hydroxyl-dependent Cas9 target engagement enables CRISPR RNA ribose replacement. Nat Rev Drug Discov. 2025;24:209–30. 10.1038/s41573-024-01086-0. 39690326 PMC13347398

[89] Yin H, Song CQ, Suresh S et al. Partial DNA-guided Cas9 enables genome editing with reduced off-target activity. Nat Chem Biol. 2018;14:311–6. 10.1038/nchembio.2559. 29377001 PMC5902734

[90] Rueda FO, Bista M, Newton MD et al. Mapping the sugar dependency for rational generation of a DNA–RNA hybrid-guided Cas9 endonuclease. Nat Commun. 2017;8:1610. 10.1038/s41467-017-01732-9. 29151576 PMC5694763

[91] Pacesa M, Loeff L, Querques I et al. R-loop formation and conformational activation mechanisms of Cas9. Nature. 2022;609:191–6. 10.1038/s41586-022-05114-0. 36002571 PMC9433323

[92] Kleinstiver BP, Pattanayak V, Prew MS et al. High-fidelity CRISPR–Cas9 nucleases with no detectable genome-wide off-target effects. Nature. 2016;529:490–5. 10.1038/nature16526. 26735016 PMC4851738

[93] Rabinowitz R, Offen D. Single-base resolution: increasing the specificity of the CRISPR–Cas system in gene editing. Mol Ther. 2021;29:937–48. 10.1016/j.ymthe.2020.11.009. 33248248 PMC7938333

[94] Wu X, Kriz AJ, Sharp PA. Target specificity of the CRISPR–Cas9 system. Quant Biol. 2014;2:59–70. 10.1007/s40484-014-0030-x. 25722925 PMC4338555

[95] Palermo G, Chen JS, Ricci CG et al. Key role of the REC lobe during CRISPR–Cas9 activation by ‘sensing’, ‘regulating’, and ‘locking’ the catalytic HNH domain. Q Rev Biophys. 2018;51:e91. 30555184 10.1017/S0033583518000070 PMC6292676

[96] Palermo G, Miao Y, Walker RC et al. Striking plasticity of CRISPR–Cas9 and key role of non-target DNA, as revealed by molecular simulations. ACS Cent Sci. 2016;2:756–63. 10.1021/acscentsci.6b00218. 27800559 PMC5084073

[97] Palermo G, Ricci CG, Fernando A et al. Protospacer adjacent motif-induced allostery activates CRISPR–Cas9. J Am Chem Soc. 2017;139:16028–31. 10.1021/jacs.7b05313. 28764328 PMC5905990

[98] Saha A, Arantes PR, Palermo G. Dynamics and mechanisms of CRISPR–Cas9 through the lens of computational methods. Curr Opin Struct Biol. 2022;75:102400. 10.1016/j.sbi.2022.102400. 35689914 PMC9398989

[99] Skeens E, Sinha S, Ahsan M et al. High-fidelity, hyper-accurate, and evolved mutants rewire atomic-level communication in CRISPR–Cas9. Sci Adv. 2024;10:eadl1045. 10.1126/sciadv.adl1045. 38446895 PMC10917355

[100] Zhang D, Hurst T, Duan D et al. Unified energetics analysis unravels SpCas9 cleavage activity for optimal gRNA design. Proc Natl Acad Sci USA. 2019;116:8693–8. 10.1073/pnas.1820523116. 30988204 PMC6500161

[101] Donohoue PD, Pacesa M, Lau E et al. Conformational control of Cas9 by CRISPR hybrid RNA-DNA guides mitigates off-target activity in T cells. Mol Cell. 2021;81:3637–49. 10.1016/j.molcel.2021.07.035. 34478654

[102] Li X, Wang C, Peng T et al. Atomic-scale insights into allosteric inhibition and evolutional rescue mechanism of *Streptococcus thermophilus* Cas9 by the anti-CRISPR protein AcrIIA6. Comput Struct Biotechnol J. 2021;19:6108–24. 10.1016/j.csbj.2021.11.010. 34900128 PMC8632846

[103] Fu Y, Sander JD, Reyon D et al. Improving CRISPR–Cas nuclease specificity using truncated guide RNAs. Nat Biotechnol. 2014;32:279–84. 10.1038/nbt.2808. 24463574 PMC3988262

[104] Rose JC, Popp NA, Richardson CD et al. Suppression of unwanted CRISPR–Cas9 editing by co-administration of catalytically inactivating truncated guide RNAs. Nat Commun. 2020;11:2697. 10.1038/s41467-020-16542-9. 32483117 PMC7264211

[105] Kiernan KA, Kwon J, Merrill BJ et al. Structural basis of Cas9 DNA interrogation with a 5′ truncated sgRNA. Nucleic Acids Res. 2025;53:gkae1164. 39657754 10.1093/nar/gkae1164 PMC11724282

[106] Lee HJ, Kim HJ, Lee SJ. Mismatch intolerance of 5′-truncated sgRNAs in CRISPR/Cas9 enables efficient microbial single-base genome editing. Int J Mol Sci. 2021;22:6457. 34208669 10.3390/ijms22126457 PMC8235755

