# Supplementary material for: CRISPR RNA architecture steers Cas9 catalysis and fidelity via a guide repeat clasp motif
Source: Nucleic Acids Res. 2026 Aug 14;54(15):gkag710. doi: 10.1093/nar/gkag710 (PMC13473882; doi:10.1093/nar/gkag710)
Supplement: Supplementary file 1 [file gkag710_supplemental_files.zip › Chilamkurthy_NAR_Supplemental.pdf]

SUPPLEMENTAL MATERIAL

CRISPR RNA architecture steers Cas9 catalysis and fidelity via a guide repeat clasp motif

Ramadevi Chilamkurthy[1], Sruthi Sudhakar[2], Adrian A. Pater[1], Michael S. Bosmeny[1], Francis Stabile[3], Adam Katolik[4], S. Harikrishna[2], Fatima El Azzouzi[1], Rolf Turk[5], Masad J. Damha[4], Sabrina Leslie[3,6], Sergey Korolev[7,*], P.I. Pradeepkumar[2,*], and Keith T. Gagnon[1,*]

[1] Department of Biochemistry, Wake Forest University School of Medicine, Winston-Salem, NC, USA.

[2] Department of Chemistry, Indian Institute of Technology Bombay, Powai, Mumbai, India.

[3] Department of Physics, McGill University, Montreal, Quebec, Canada.

[4] Department of Chemistry, McGill University, Montreal, Quebec, Canada.

[5] Integrated DNA Technologies, Coralville, Iowa, USA.

[6] Michael Smith Laboratory and Department of Physics and Astronomy, University of British Columbia, Vancouver, British Columbia, Canada

[7] Department of Biochemistry and Molecular Biology, School of Medicine, Saint Louis University, St. Louis, Missouri, USA.

[§] These authors contributed equally.

[*] Corresponding authors: pradeep@chem.iitb.ac.in, sergey.korolev@health.slu.edu, keith.gagnon@wfusm.edu

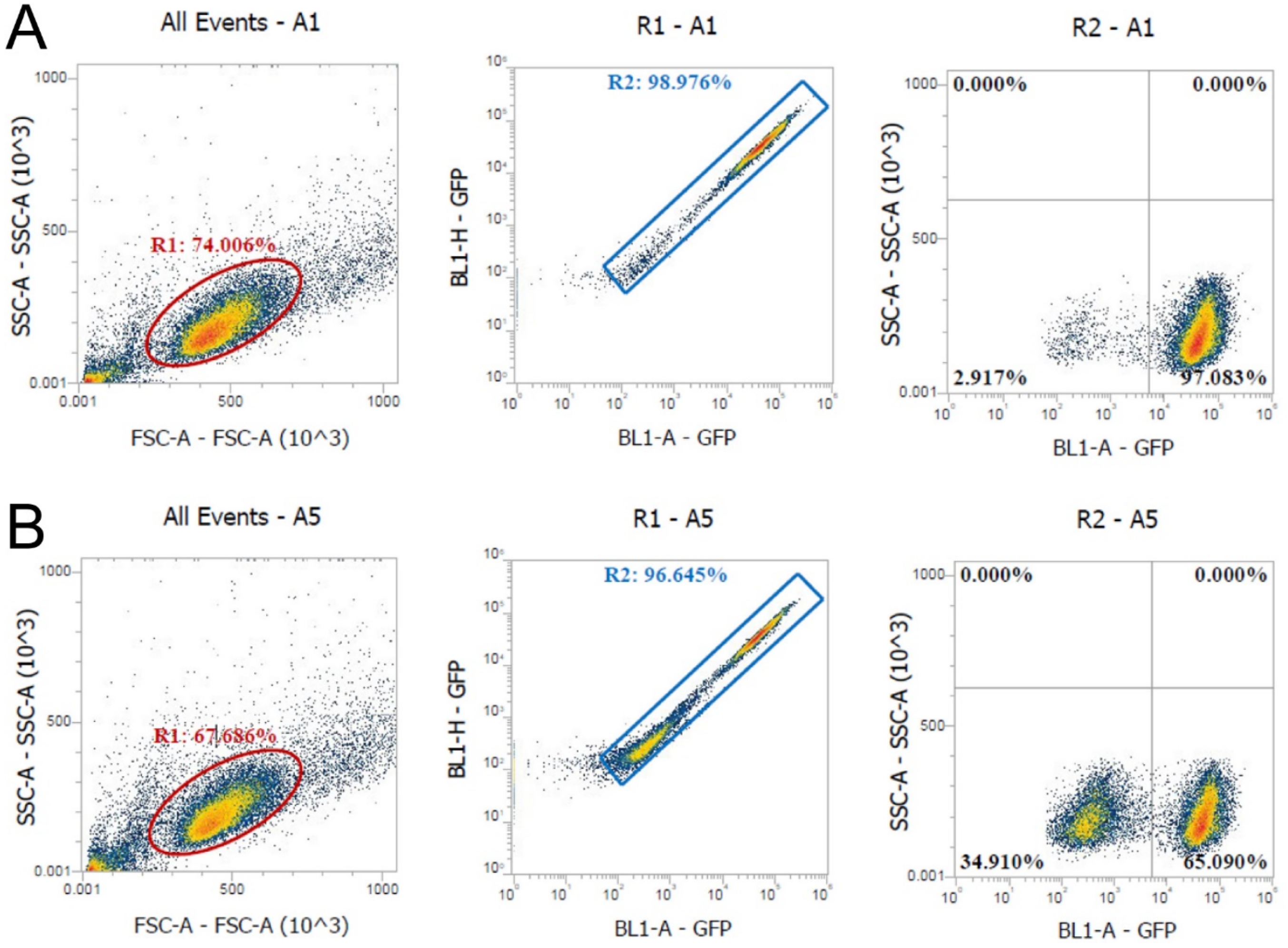

Figure S1. Representative gating and example data for flow cytometry quantification of EGFP editing in HEK 293T cells. Flow cytometry of (**A**) non-treated and (**B**) guide RNA transfected HEK293T cells. Gate 1: forward vs. side scatter (FSC vs SSC) density plot (for identifying cell population of interest and excluding debris). Gate 2: BL1-Area vs. BL-1-Height density plot (for excluding doublets). Gate 3: BL1-A vs. SSC-A two parameter density plot (for distinguishing EGFP positive cells and non fluorescent cells).

A **Selected Guides from 255 Spacer (Alt-R) Screen**

Gene	dgRNA Editing (%)	sgRNA Editing (%)	Protospacer
ANKRD52	30.23	83.10	CCACGTTGAGGCTGCTCAAC
BLVRA	44.67	76.00	ACACGAAGCCAATCAGGTTC
BOP1	52.89	72.18	GTACGATGACTTCCCCCACG
C1orf112	72.47	91.29	TTATTATGGACTGGTGCTTA
BNIPL	65.69	83.39	GGTGTTCCGAATGGGACCAC
ATAD3A	31.86	34.13	AAGAATGCCACGCTTGTCGC
ANKEF1	56.34	57.31	CCCATTCGGTCTTGCACATC
ATP6V0A2	88.40	88.83	TGTTTGGATAGGGGTACACG
AEBP1	94.45	94.84	GAACTCCTCGATCTCGTCGT
ATAD3C	76.96	77.26	GTCAAAGGACGCCCTGAATC
ANKRD13D	96.29	86.18	CTTCGACACAATGCCAACGT
C19orf84	84.45	73.62	GGGGGCCTACACCTTCCAAC
C16orf90	70.55	54.82	CGCACCCCCCAACATCTACG
ARL2	90.48	69.68	AACTACTTTGAGAGCACCGA
C17orf99	61.79	35.06	GCGGGCCAACTTCACTCTGC

B

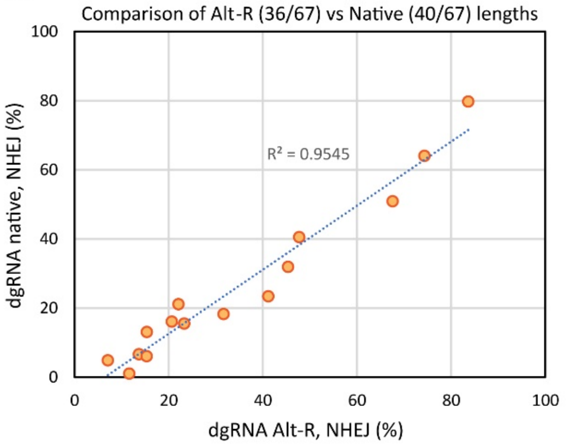

C

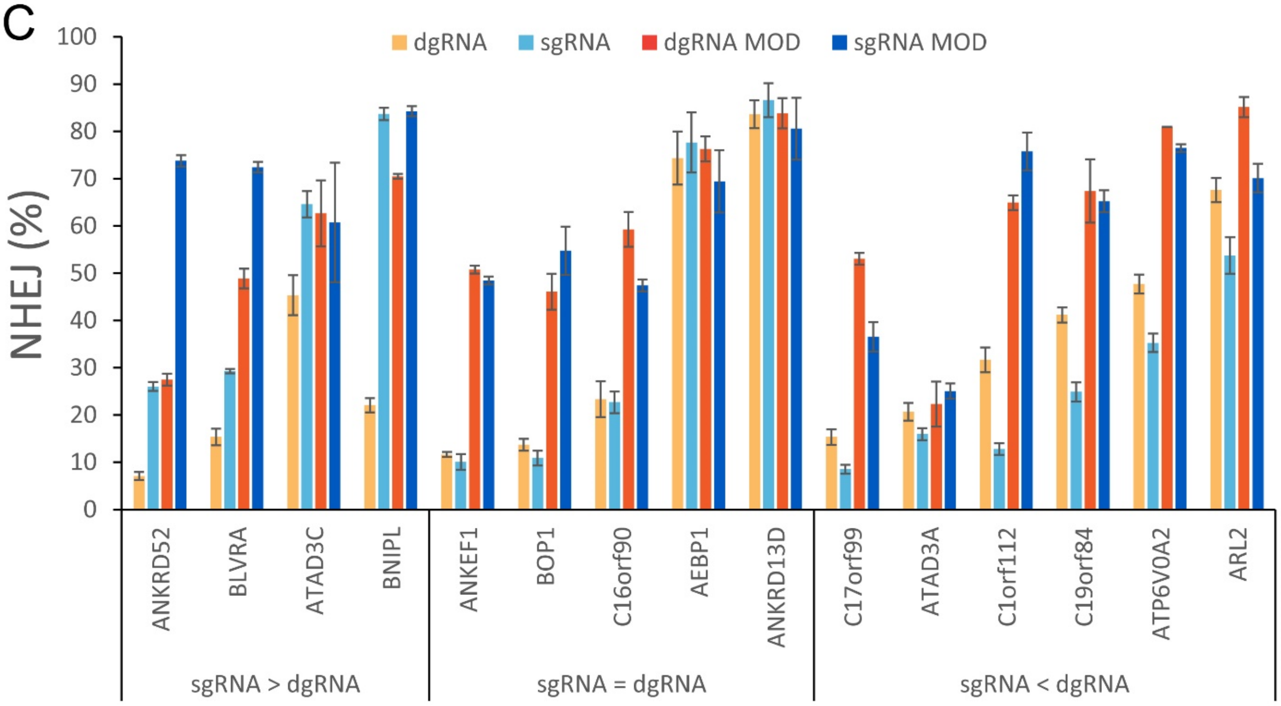

Figure S2. Editing of a subset of endogenous genes selected from 255 endogenous loci with native and chemically modified sgRNA- and dgRNA-guided Cas9. (**A**) Fifteen guides targeting endogenous genes were initially selected based on performance in a screen of 255 loci using a commercially available AltR-CRISPR system. Five guides were selected where sgRNA outperformed dgRNA, five where sgRNA and dgRNA performed equally, and five where dgRNA outperformed sgRNA. (**B**) sgRNAs and dgRNAs were resynthesized as native, unmodified guides and the native unmodified dgRNAs compared to chemically modified AltR-CRISPR dgRNAs. Chemical modification (AltR-CRISPR), proprietarily designed to increase stability of the crRNA-tracrRNA repeat and resist nuclease degradation, generally improved the gene editing performance of all fifteen guides with high correlation (R^2 = 0.9545). However, overall trends remained the same and modification did not generally improve the performance of any individual guide RNA, as evidenced by the R^2 value, suggesting that performance is intrinsic to sequence-structural features not affected by the AltR-CRISPR modification scheme. (**C**) Comparison of gene editing activity for all fifteen guides as native and chemically modified (MOD) (AltR-CRISPR) sgRNAs and dgRNAs. Guides are grouped based on performance of native (unmodified) sgRNA versus native (unmodified) dgRNA. Error is reported as S.D. n=3 experimental replicates.

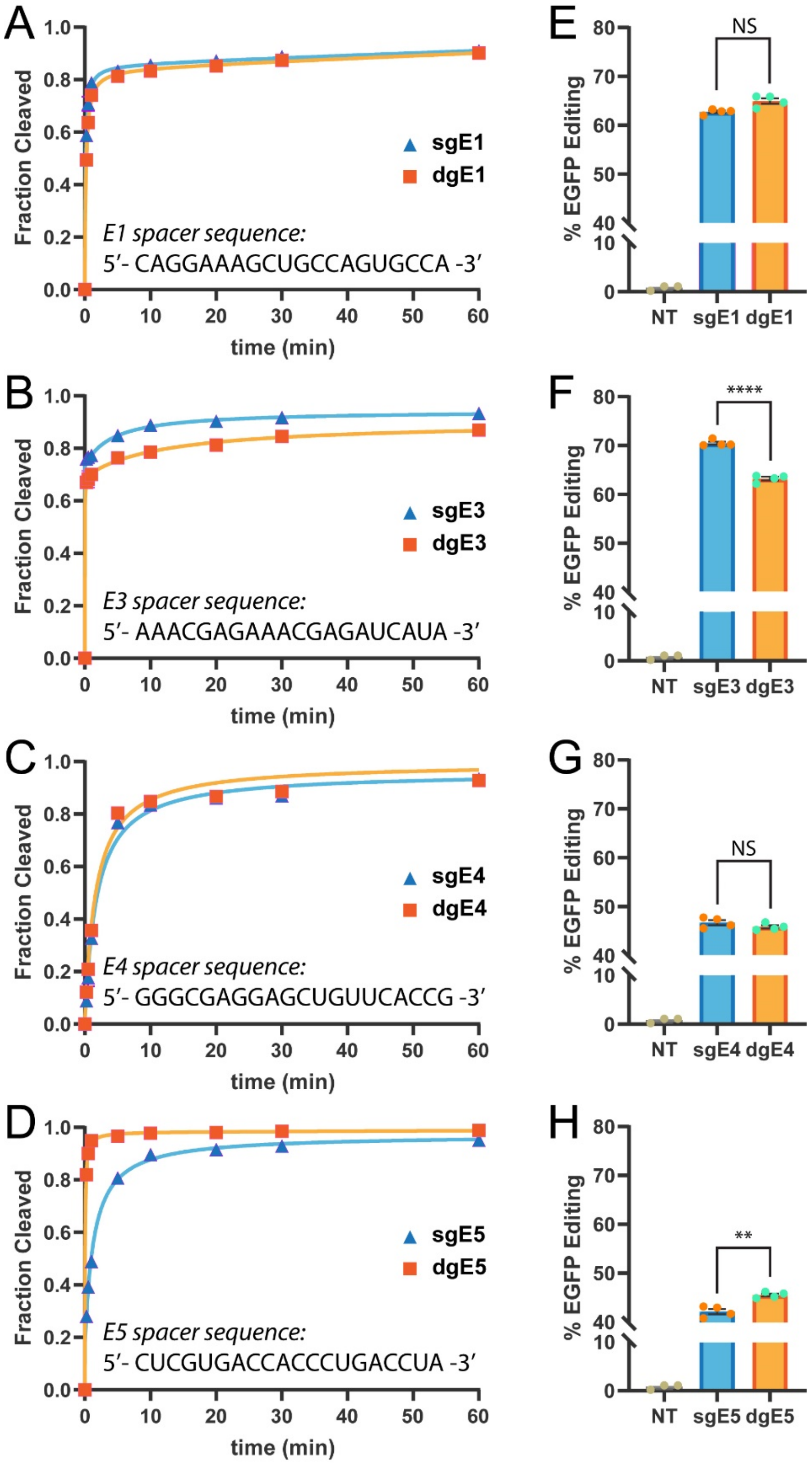

<u>Figure S3</u>. Catalytic activity of Cas9 guided by sgRNA and dgRNA bearing E1, E3, E4, and E5 spacer sequences. (**A**-**D**) *In vitro* time course cleavage assays for E1, E3, E4, and E5 spacer sequences (n=2 experimental replicates). (**E**-**H**) Cell-based EGFP editing (knockout) assays for E1, E3, E4, and E5 spacer sequences (n=4 experimental replicates). Error is reported as S.E.M. Unpaired t-test p-values, ** = 0.0016, **** = <0.0001, NS = not significant.

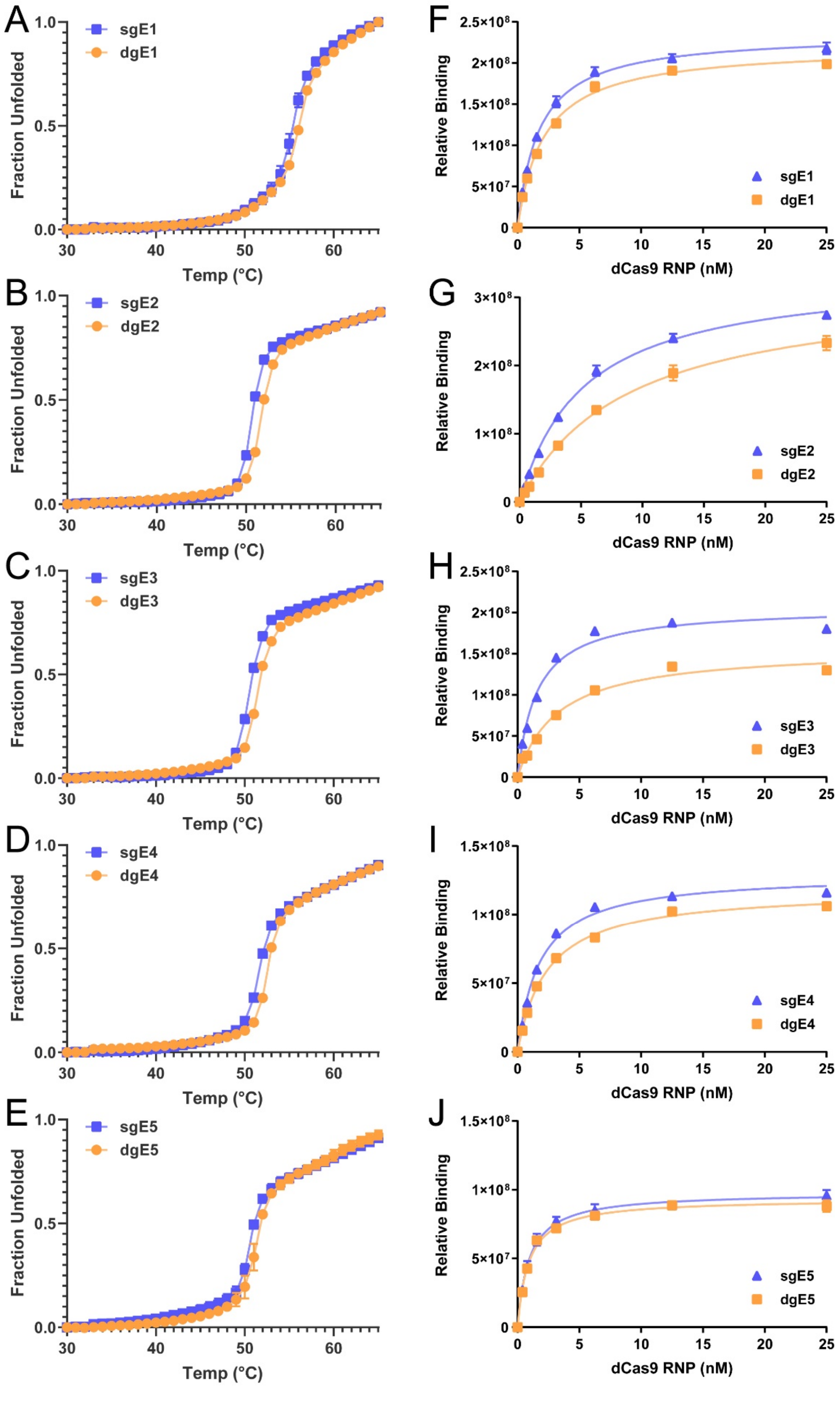

<u>Figure S4</u>. Thermal stability and target DNA binding of Cas9 RNPs guided by sgRNA and dgRNA bearing E1, E2, E3, E4, and E5 spacer sequences. (**A**-**E**) RNP thermal stability measured by absorbance at 280 nm (n=2 experimental replicates). (**F**-**J**) dsDNA target binding affinity measured by dot blot equilibrium binding (n=2 experimental replicates). Relative Binding is densitometry values from phosphorimager scanning normalized to samples lacking dCas9 RNP (background radiolabeled DNA binding). Error is reported as S.E.M.

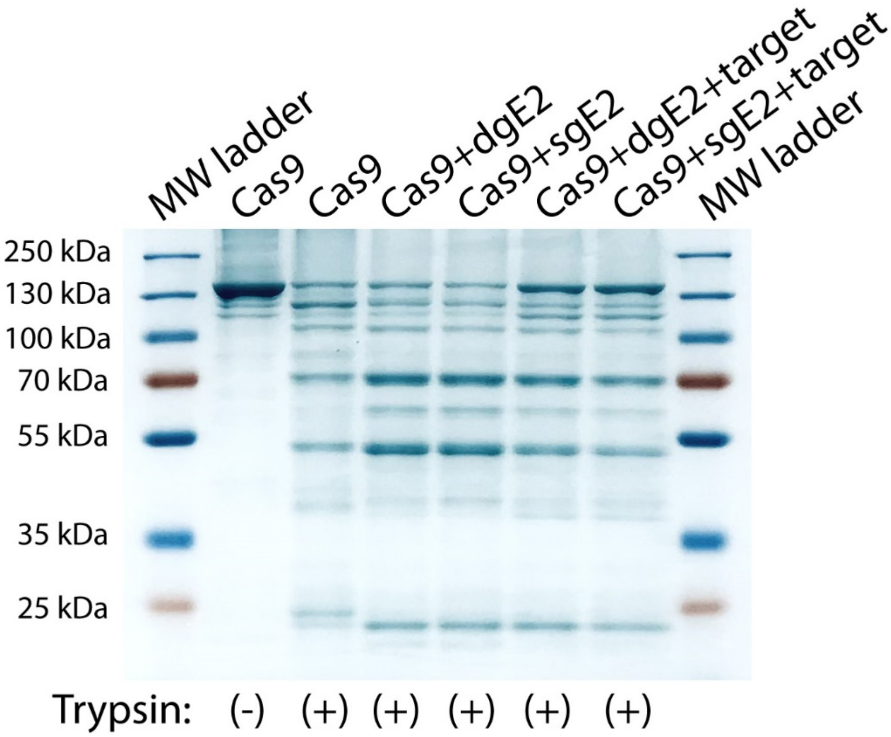

<u>Figure S5</u>. **No major differences are observed in trypsin hydrolysis cleavage patterns between Cas9 assembled with dgE2 and sgE2 or bound to E2 DNA target.** Resolution of limited trypsin hydrolysis products by SDS-PAGE for Cas9 in RNP and ternary (target bound) complexes followed by staining with coomassie brilliant blue G-250.

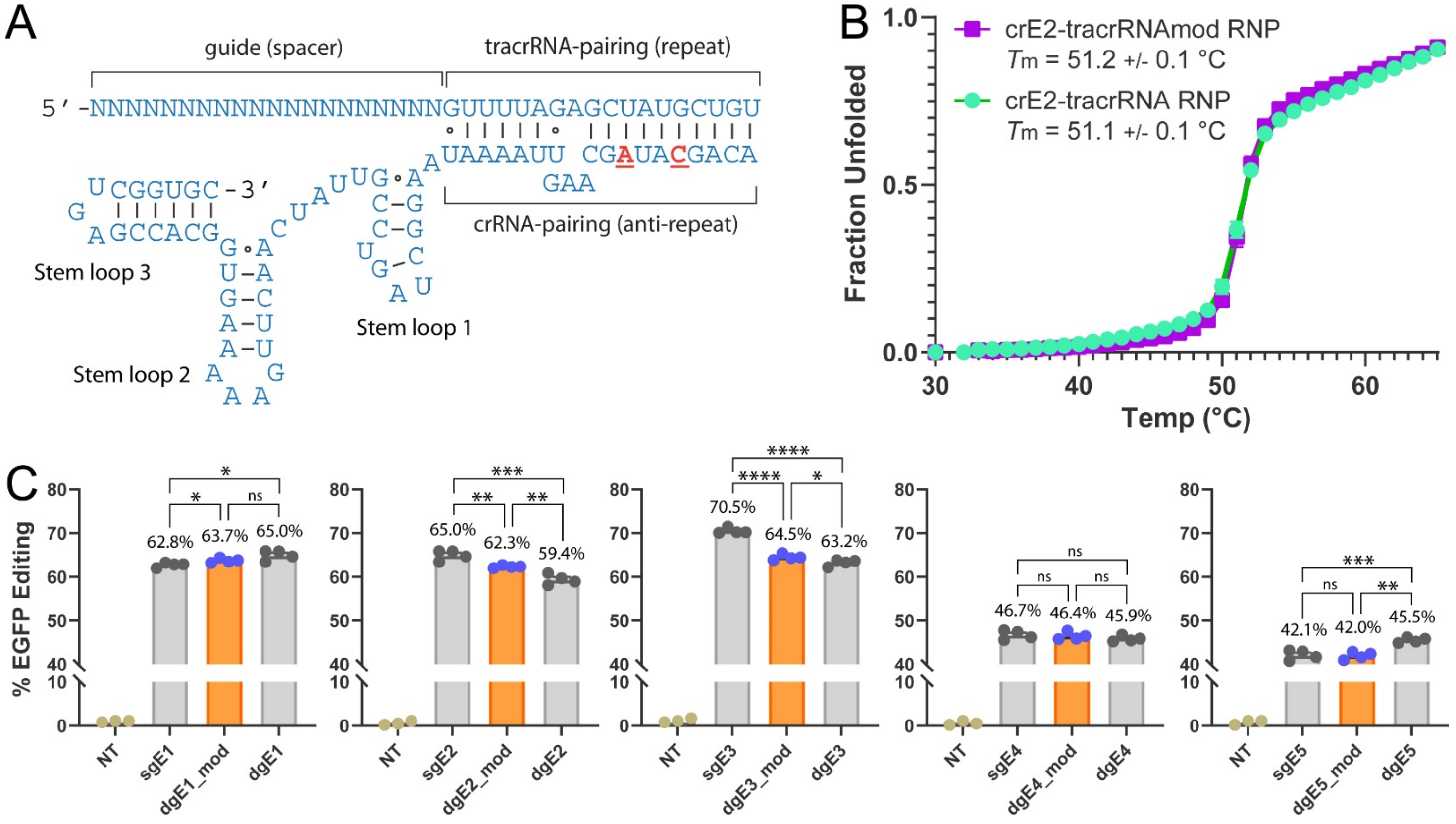

Figure S6. Thermal stability and gene editing activity of dgRNA-guided Cas9 with locked nucleic acid modifications in the tracrRNA repeat sequence. (**A**) Schematic representation of dgRNA. Locked nucleic acid (LNA) modifications are indicated by red, bold, underlined nucleotides. (**B**) Thermal stability of Cas9 RNPs assembled with E2 crRNA and modified and unmodified tracrRNA measured by absorbance at 280 nm (n=2). Range of values reported as +/-. (**C**) Cell-based EGFP editing assays for dgRNAs with chemically modified tracrRNA (tracrRNAmod) (n=4). Gene editing percentages shown above bar graphs are reported as the mean. Error is S.E.M. Unpaired t-test p-value: n.s. = not significant, * > 0.05, ** > 0.01, *** > 0.005, **** > 0.0001.

A

Folding Features Characterized with Vienna RNA

Architecture	Structural Feature	H statistic	p-value	Significant?
dual	MFE_kcal_mol	2.98	0.2250	No
dual	frac_paired	2.22	0.3292	No
dual	hairpins	1.36	0.5061	No
dual	max_nesting	3.23	0.1985	No
dual	GC_content	4.35	0.1137	No
single	MFE_kcal_mol	2.40	0.3010	No
single	frac_paired	4.21	0.1221	No
single	hairpins	1.84	0.3989	No
single	max_nesting	1.71	0.4254	No
single	GC_content	4.35	0.1137	No

B

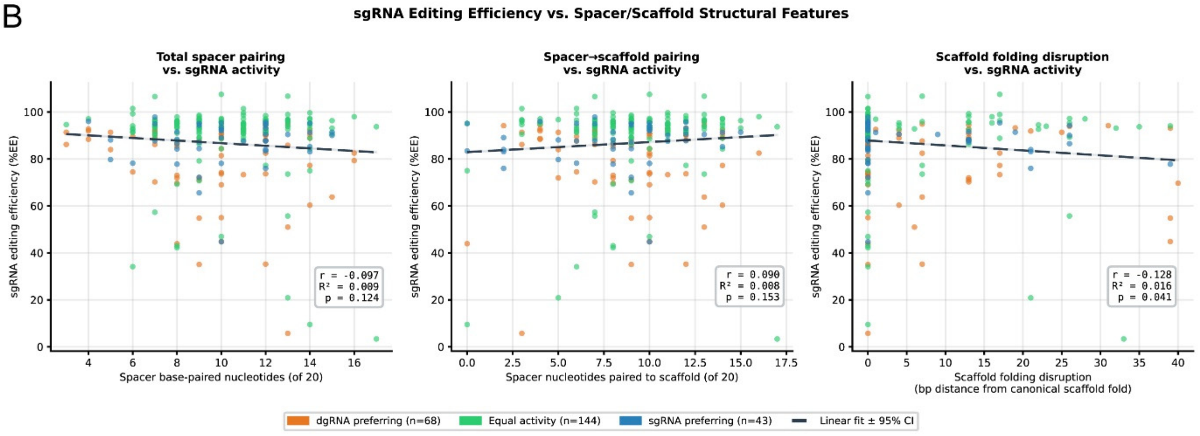

C

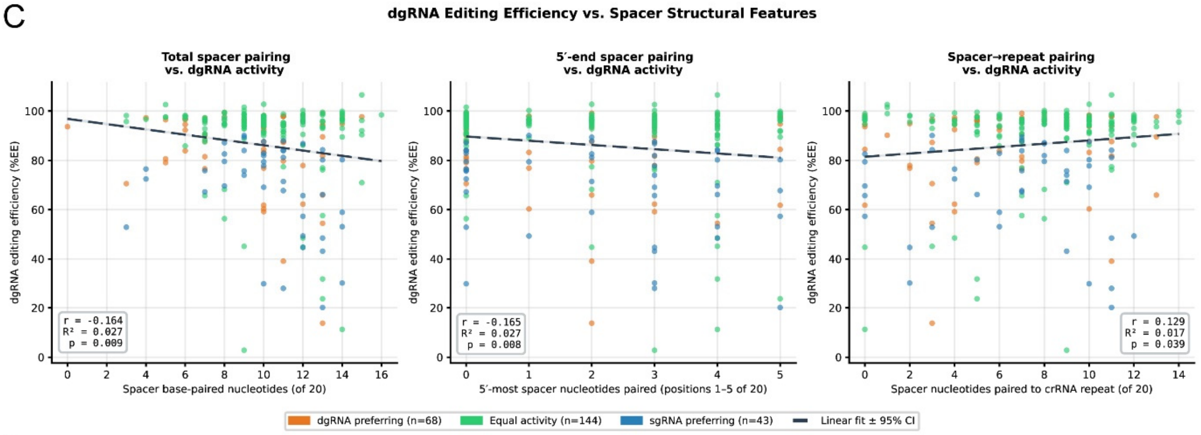

D

Pearson Correlation Summary: Structural Features vs. Editing Efficiency

Structural Metric	Architecture	n	Overall Pearson r	Overall R^2	Overall p-value	r | dgRNA subset (n=68)	r | equal subset (n=144)	r | dgRNA subset (n=43)
Total spacer pairing	sgRNA	255	-0.097	0.009	0.124	-0.176	-0.121	+0.090
Spacer -> scaffold pairing	sgRNA	255	+0.090	0.008	0.153	+0.023	+0.114	+0.075
Scaffold fold disruption	sgRNA	255	**-0.128**	0.016	**0.041**	-0.043	-0.146	-0.018
Total spacer pairing	dgRNA	255	**-0.164**	0.027	**0.009**	-0.169	-0.131	-0.292
5′-end pairing (nts 1-5)	dgRNA	255	**-0.165**	0.027	**0.008**	-0.129	**-0.179***	-0.229
Spacer -> repeat pairing	dgRNA	255	**+0.129**	0.017	**0.039**	+0.146	+0.155	-0.086

** p < 0.05 within that subset. Overall p-values in bold red = significant at α = 0.05. Rows 1–3: sgRNA-context metrics vs. sgRNA %EE. Rows 4–6: 2-part-context metrics vs. 2-part %EE.*

Figure S7. Structural features of guide RNA folding are poor predictors of absolute editing efficiency and reveal only modest architecture-dependent associations. (**A**) Kruskal-Wallis test results for ten structural features (five per guide architecture: MFE, fraction paired, hairpin count, maximum nesting depth, and GC content) comparing distributions across guide RNA editing-preference architectures. No feature reaches significance at $\alpha = 0.05$. (**B**) Scatter plots of sgRNA editing efficiency (%EE) versus three structural metrics measured in the sgRNA scaffold: total spacer base-pairing (left), spacer → scaffold pairing (middle), and scaffold fold disruption relative to the canonical structure (right). Linear fit with 95% confidence interval is shown. Correlation statistics are shown (inset). (**C**) Scatter plots of dgRNA editing efficiency versus three structural metrics measured in the dgRNA scaffold: total spacer base-pairing (left), 5′-end spacer pairing (nucleotide positions 1–5; middle), and spacer → crRNA repeat pairing (right). (**D**) Pearson correlation summary table for all six structural metrics versus editing efficiency, including overall statistics and subset-specific correlations by format-preference group.

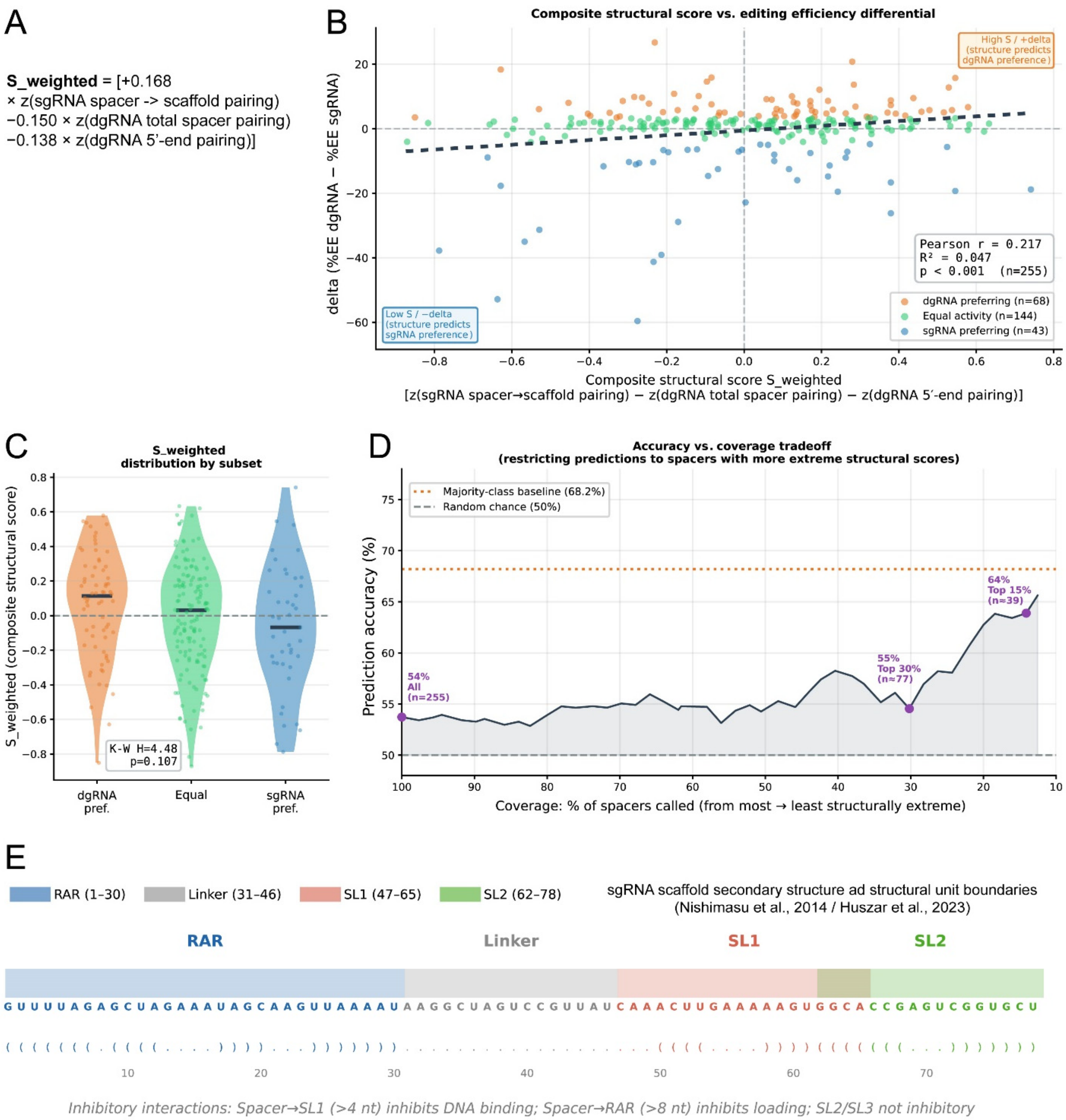

F

Metric	r (Δ%EE)	p-value	r (sgRNA %EE)	p-value
Spacer→RAR pairings	−0.057	0.365	−0.080	0.202
Spacer→SL1 pairings	+0.078	0.216	+0.015	0.815
Spacer→SL2 pairings	+0.063	0.317	−0.101	0.109
SL1 dimer MFE (RNAcofold)	+0.034	0.586	+0.078	0.216
RAR dimer MFE (RNAcofold)	+0.117	0.063	+0.080	0.201

G

Guide	sp→RAR (sgRNA)	sp→SL1 (sgRNA)	GCC motif	S_weighted
E1	0	0	Yes	-0.342
E2	0	0	No	+0.008
E3	0	1	No	+0.267
E4	5	0	No	-0.180
E5	0	0	No	+0.699

Figure S8. Composite structural scoring framework and scaffold unit dissection for the 255 reference spacer dataset and EGFP guides. (**A**) Formula defining the composite structural score S_weighted, derived from a multiple regression of z-scored structural features against Δ%EE. (**B**) Scatter plot of S_weighted versus Δ%EE (%EE dgRNA − %EE sgRNA) across all 255 reference spacers (Pearson r = 0.217, $R^2 = 0.047$, $p < 0.001$), color-coded by format-preference group. (**C**) Violin plots of S_weighted distributions for dgRNA-preferring, equal-activity, and sgRNA-preferring spacer subsets. Kruskal-Wallis test result is shown (inset). (**D**) Accuracy–coverage tradeoff curve showing prediction accuracy when restricting calls to spacers with the most extreme S_weighted values (from most to least structurally extreme, x-axis runs right to left from 100% to 10% coverage). Majority-class baseline (68.2%) and random chance (50%) are shown as reference lines. (**E**) Annotated sgRNA scaffold sequence with structural unit boundaries (repeat-antirepeat, RAR; Linker; stemloop 1, SL1; stemloop 2, SL2) and canonical dot-bracket secondary structure. (**F**) Pearson correlation table for scaffold-unit-specific structural metrics (spacer → RAR, spacer → SL1, and spacer → SL2 pairings; SL1 and RAR dimer MFEs from RNAcofold) versus both Δ%EE and sgRNA %EE. P-values indicate no statistical significance. (**G**) Per-guide summary table for EGFP spacers E1–E5 listing spacer → RAR pairings (sgRNA context), spacer → SL1 pairings (sgRNA context), presence of a GCC motif, and S_weighted score.

A

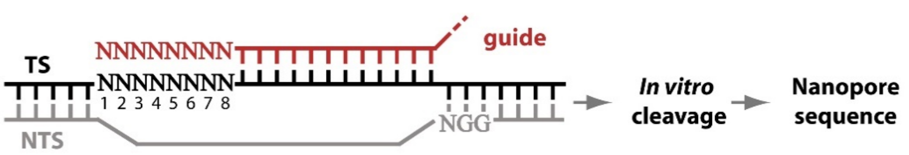

B

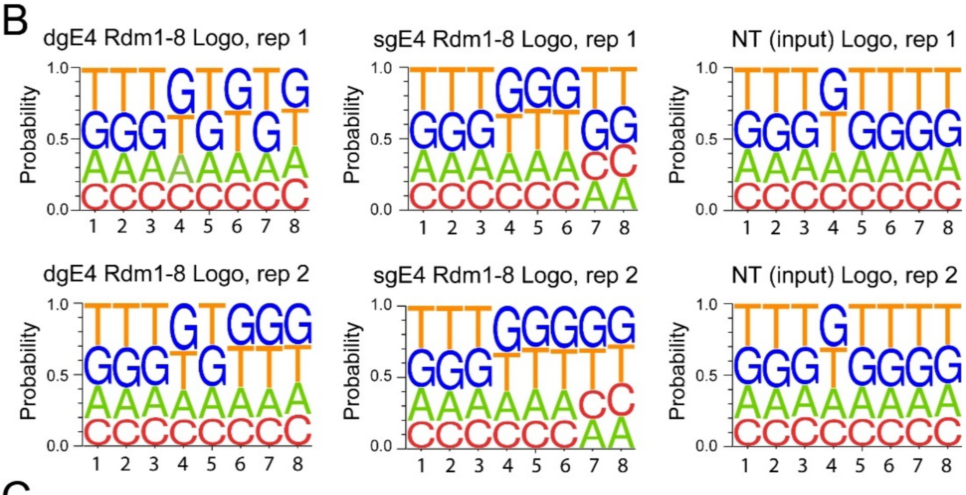

C

Position	sgE4 A	dgE4 A	sgE4 C	dgE4 C	sgE4 G	dgE4 G	sgE4 T	dgE4 T	p-value	
1	23.2%	23.4%	19.2%	19.0%	27.2%	27.5%	30.3%	30.1%	1.46E-04	***
2	21.6%	21.8%	18.9%	18.7%	28.3%	28.5%	31.2%	31.1%	1.15E-02	*
3	21.8%	21.8%	20.7%	20.1%	27.9%	28.3%	29.6%	29.9%	7.67E-10	****
4	20.7%	20.5%	19.2%	18.7%	32.7%	33.2%	27.3%	27.6%	2.95E-12	****
5	22.1%	22.1%	19.7%	19.1%	29.2%	29.1%	29.0%	29.6%	1.77E-10	****
6	21.2%	21.2%	20.0%	19.5%	30.0%	30.0%	28.8%	29.3%	7.04E-08	****
7	20.4%	20.3%	20.5%	19.9%	29.6%	29.8%	29.5%	30.0%	1.19E-10	****
8	22.4%	22.5%	23.7%	22.2%	26.9%	28.9%	27.0%	26.4%	8.13E-108	****

D

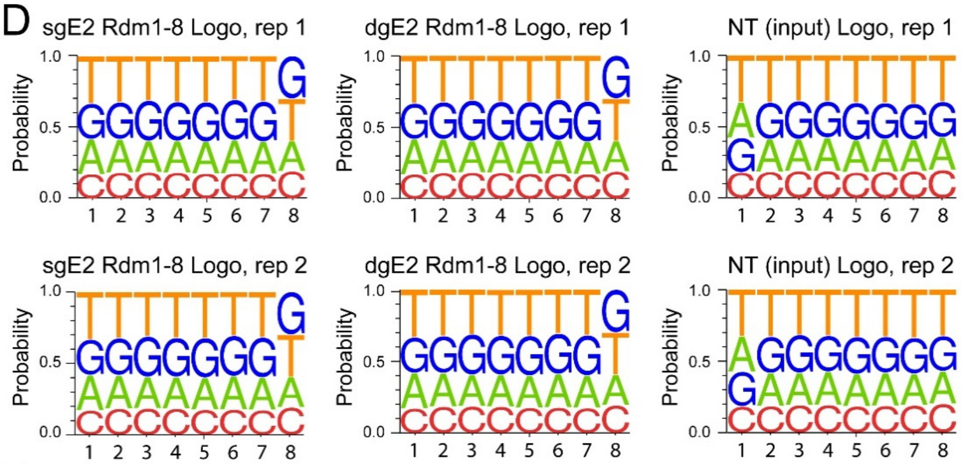

E

Position	sgE2 A	dgE2 A	sgE2 C	dgE2 C	sgE2 G	dgE2 G	sgE2 T	dgE2 T	p-value	
1	24.6%	24.9%	17.5%	17.3%	24.9%	25.7%	33.0%	32.1%	1.36E-01	NS
2	23.3%	22.7%	18.7%	18.9%	26.0%	26.2%	31.9%	32.1%	4.19E-01	NS
3	21.9%	21.4%	18.7%	18.7%	28.1%	28.4%	31.4%	31.5%	6.33E-01	NS
4	22.3%	22.1%	18.5%	19.0%	27.4%	28.3%	31.8%	30.6%	1.09E-02	*
5	22.0%	21.1%	17.8%	18.0%	28.0%	28.3%	32.2%	32.5%	1.12E-01	NS
6	23.1%	22.9%	18.1%	18.1%	28.2%	28.6%	30.5%	30.4%	5.05E-01	NS
7	22.4%	21.9%	17.6%	17.5%	28.3%	29.1%	31.8%	31.5%	1.84E-01	NS
8	21.2%	20.6%	19.3%	18.9%	30.1%	31.3%	29.4%	29.2%	3.88E-02	*

Figure S9. Determination of sequence preferences induced by sgRNAs versus dgRNAs for E2 and E4 spacers using nanopore sequencing of *in vitro* cleavage products. (**A**) Illustration of dual randomization of the 8 PAM-distal nt of guide RNA and target DNA to identify potential sequence preferences for Cas9 guided by dgRNA versus sgRNA. (**B**) Sequence logos generated for replicates 1 and 2 after nanopore sequencing of E4-Rdm8 target cut with partially randomized dgE4 and sgE4. (**C**) Breakdown of average percent occurrence of individual nt at each position from nanopore sequencing and statistical significance of results at each position. (**D**) Sequence logos generated for replicates 1 and 2 after nanopore sequencing of E2-Rdm8 target cut with partially randomized dgE2 and sgE2. (**E**) Breakdown of average percent occurrence of individual nt at each position from nanopore sequencing and statistical significance of results at each position.

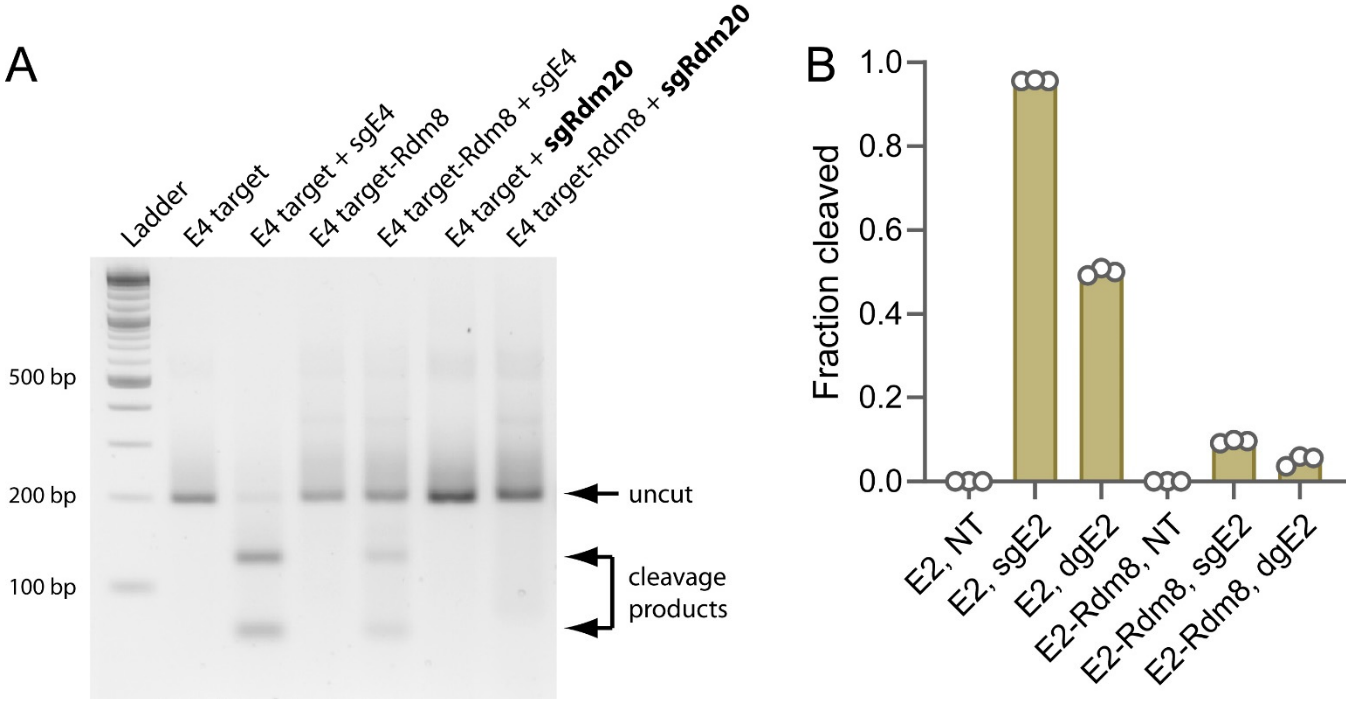

Figure S10. (**A**) Separation of *in vitro* cleavage products on an agarose gel. Target DNA is either E4 or E4 with the first 8 nts of spacer target region randomized (E4 target-Rdm8). Guides used were either perfectly complementary sgRNA (sgE4) or sgRNA with all 20 spacer nts randomized (sgRdm20). (**B**) Quantification of *in vitro* cleavage products from agarose gel separation. Target DNA is either E2 or E2 with the first 8 nuts of the spacer target region randomized (E2-Rdm8). Guides used were either perfectly complementary sgRNA (sgE2) or dgRNA (dgE2) (n=3).

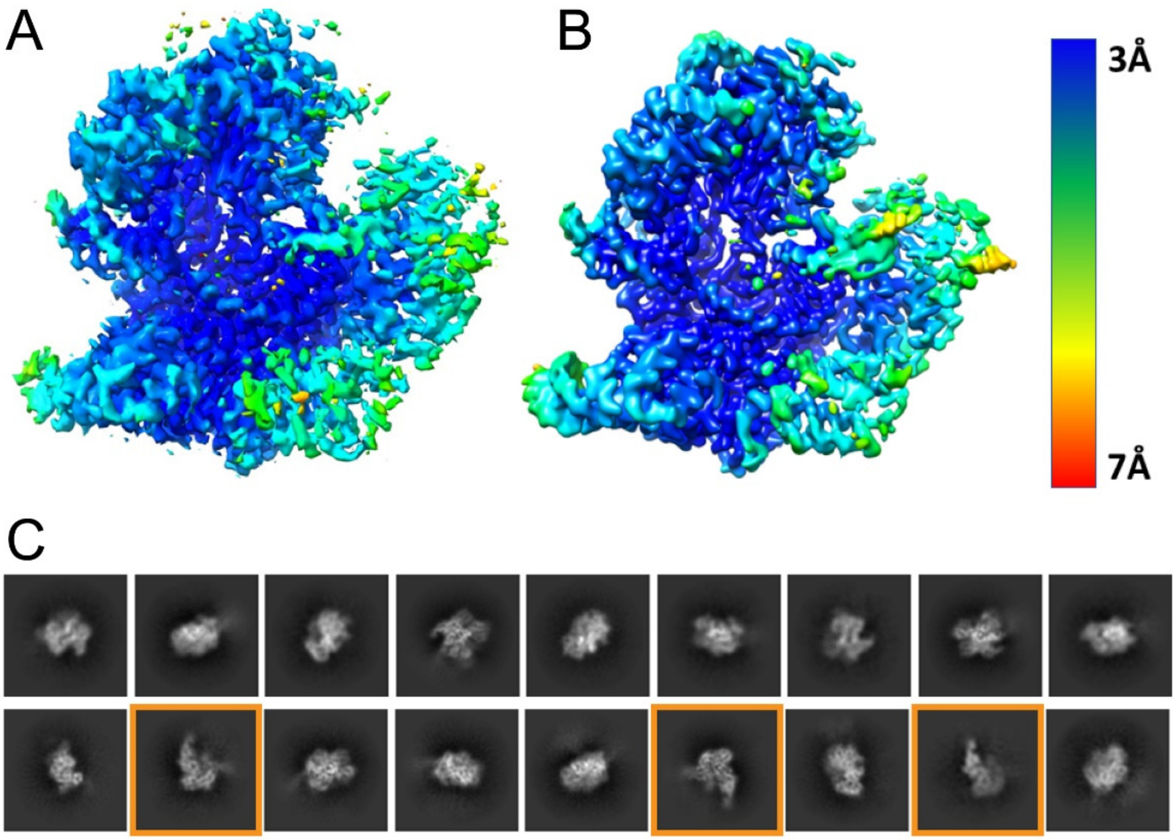

Figure S11. Electron density resolution and 2D classification of single and dual guide Cas9 ternary complexes solved by cryo-EM. Final electron density maps for closed forms of sgE2 (**A**) and dgE2 (**B**) ternary complexes solved by cryo-EM 3D reconstruction color-coded by local resolution of 3-7 angstroms. (**C**) 2D classifications for dgE2 Cas9 ternary complexes. 2D classifications enclosed in orange boxes are classified as “open” complexes.

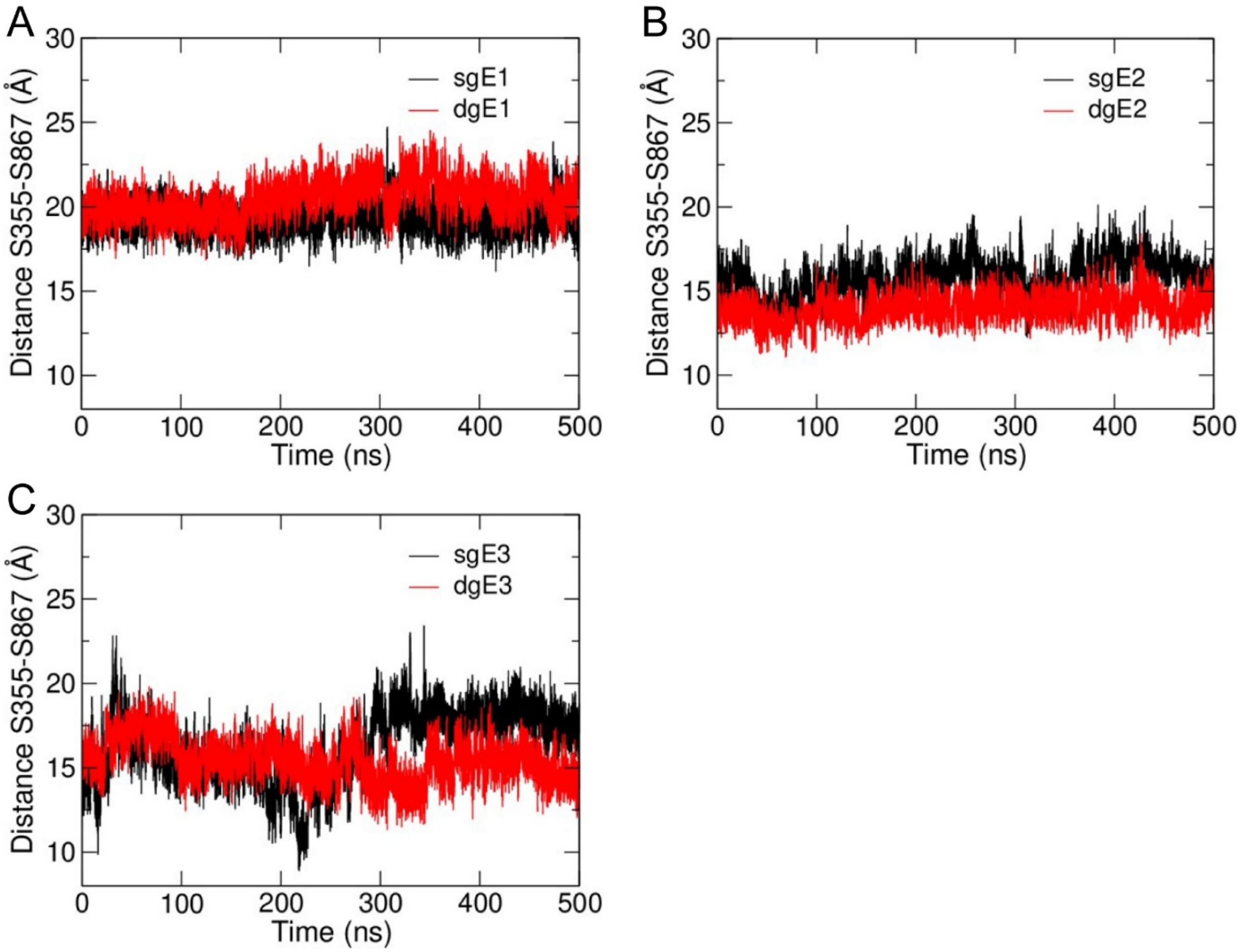

<u>Figure S12</u>. Time dependent distance plots of the S355-S867 amino acids in modeled sgRNA and dgRNA Cas9 ternary complexes with E1 (**A**), E2 (**B**), and E3 (**C**) spacer sequences from the 500 ns GAMD trajectories.

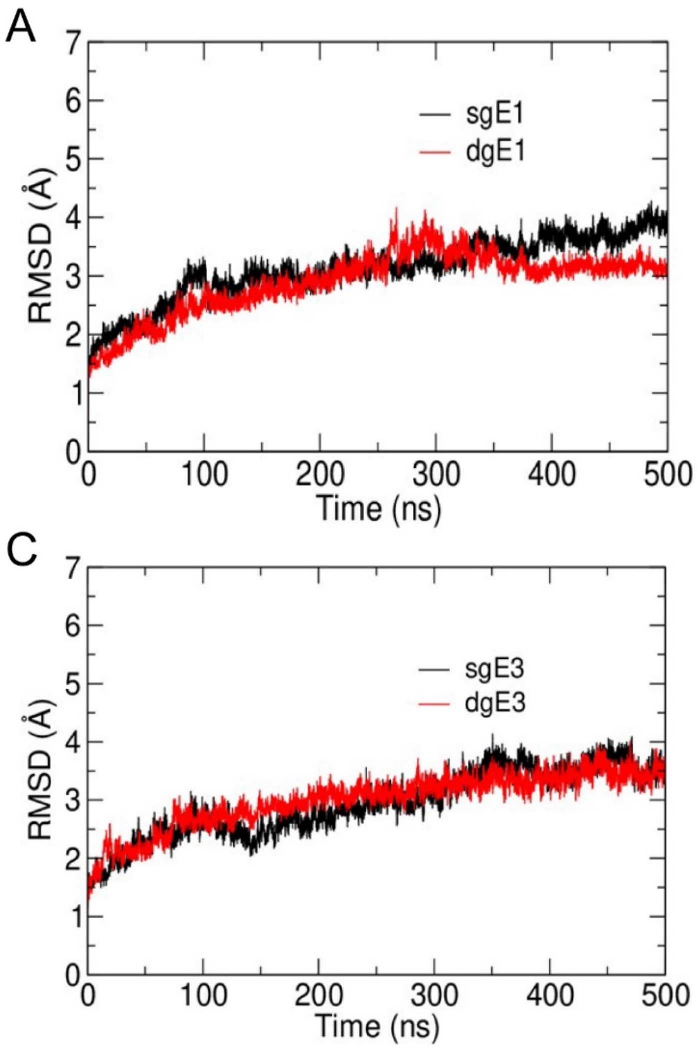

<u>Figure S13</u>. RMSD graphs of the protein backbone in modeled sgRNA and dgRNA Cas9 ternary complexes with E1 (**A**), E2 (**B**), and E3 (**C**) spacer sequences. The RMSD values were calculated from the 500 ns GAMD trajectories.

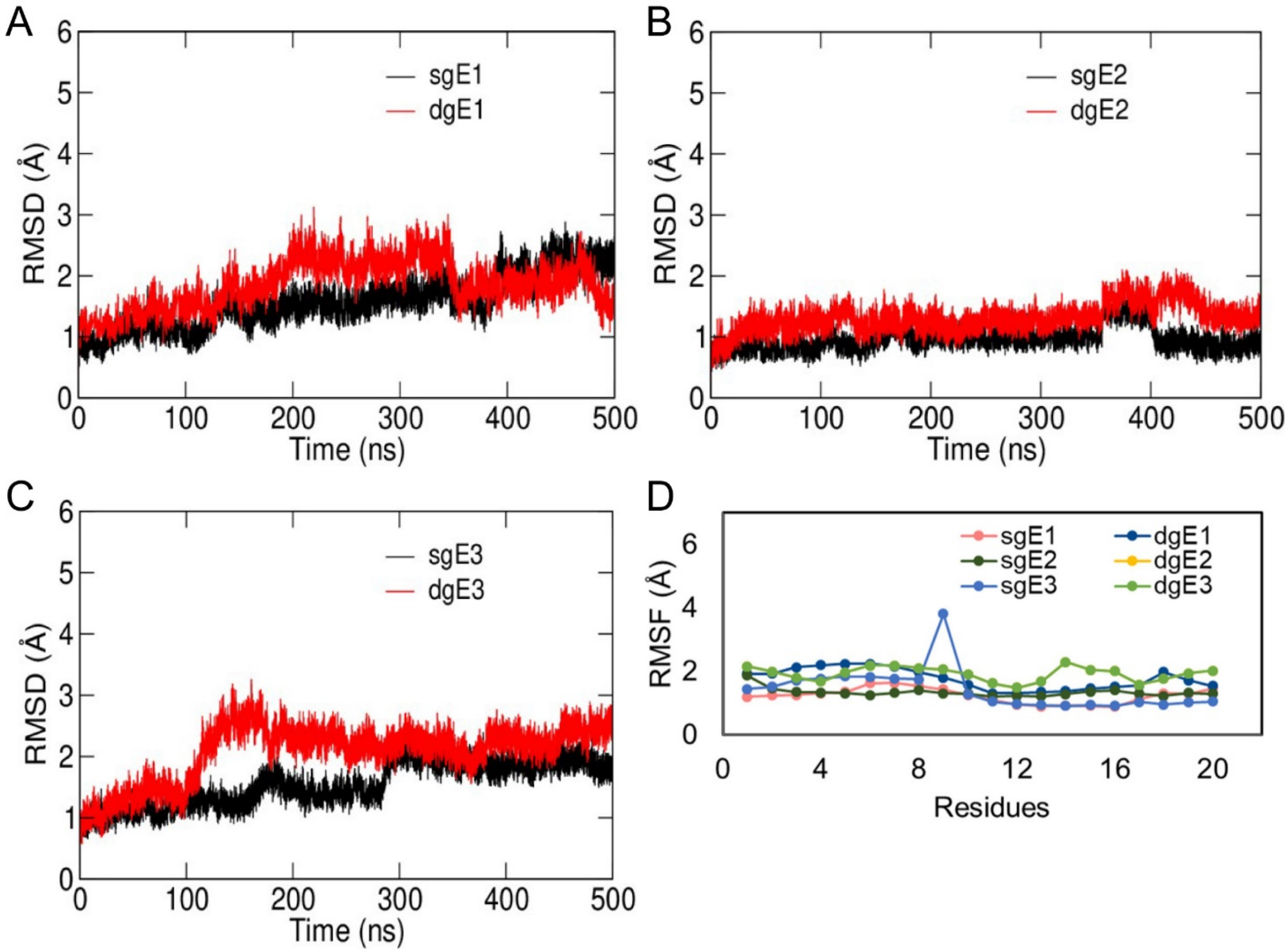

<u>Figure S14</u>. RMSD graphs of the guide RNA backbone in modeled sgRNA and dgRNA Cas9 ternary complexes with E1 (**A**), E2 (**B**), and E3 (**C**) spacer sequences. The RMSD values were calculated from the 500 ns GAMD trajectories. Only the first 20 nucleotides were considered for the plots shown. (**D**) Per nucleotide RMSF of the guide RNA in modeled sgRNA and dgRNA Cas9 ternary complexes with E1, E2, and E3 spacer sequences

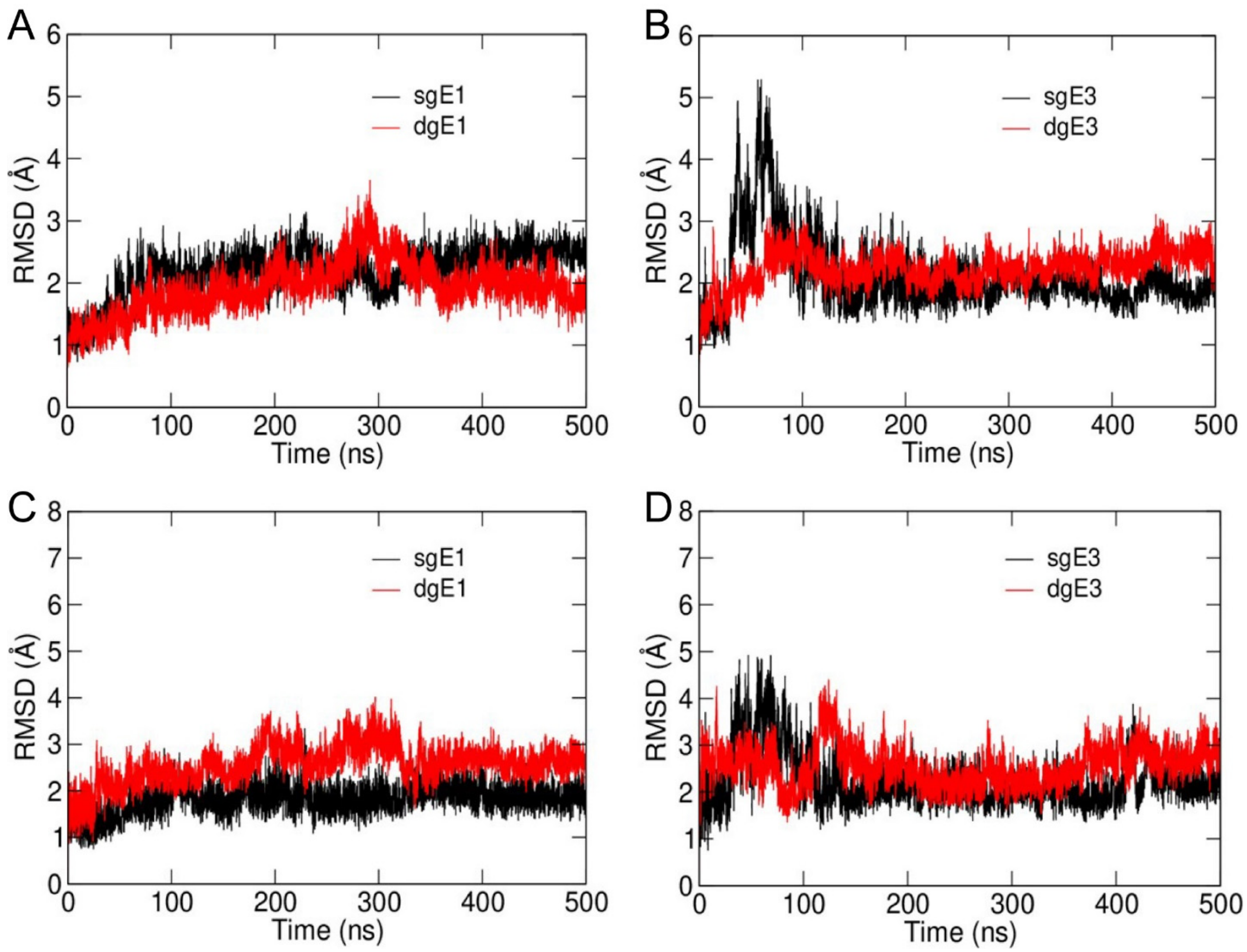

<u>Figure S15</u>. RMSD graphs of the DNA backbone in modeled sgRNA and dgRNA Cas9 ternary complexes for the target strand (TS) of E1 (**A**) and E3 (**B**) spacer sequences and for the non-target strand (NTS) of E1 (**C**) and E3 (**D**) spacer sequences. The RMSD values were calculated from the 500 ns GAMD trajectories.

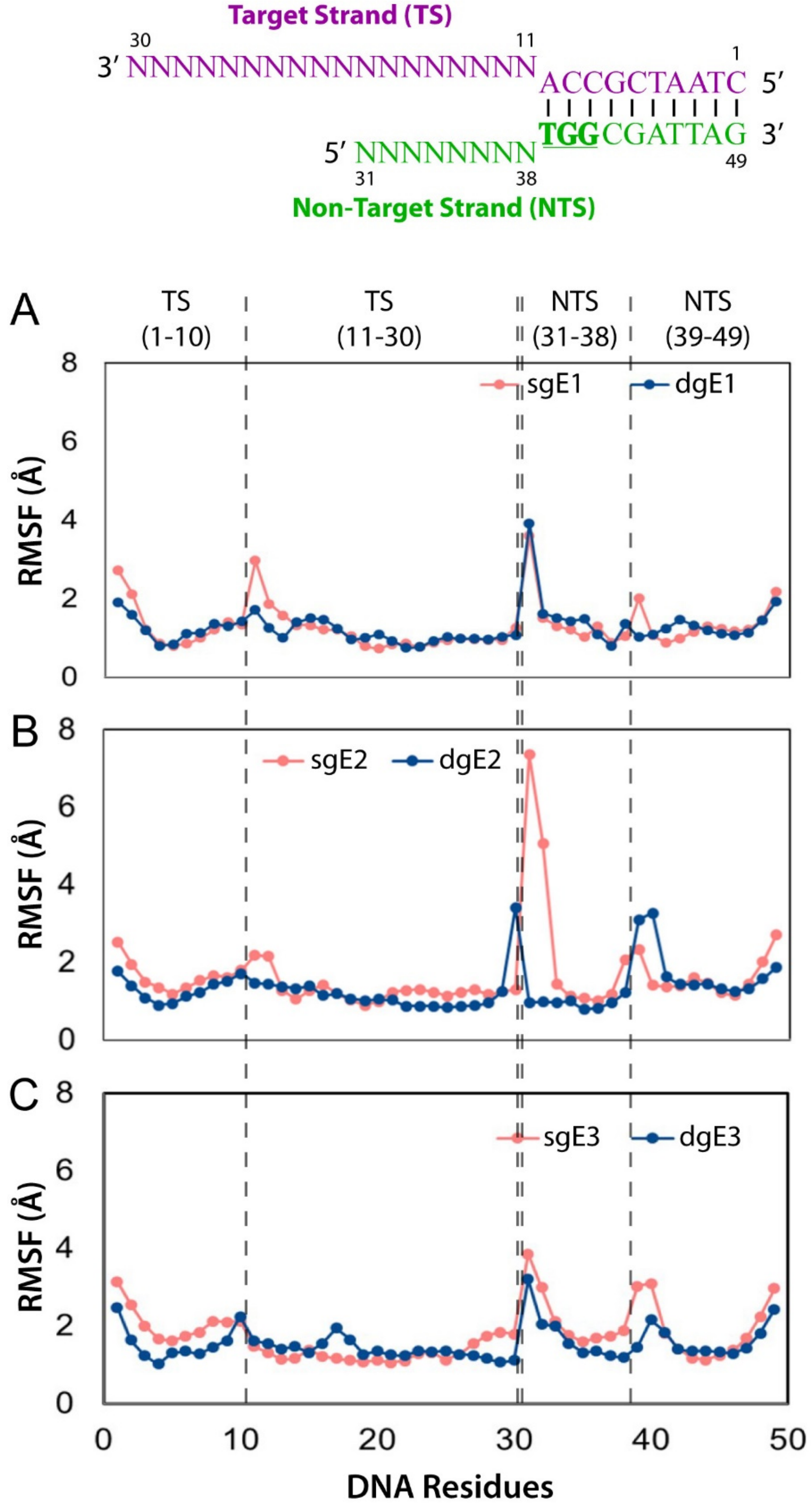

Figure S16. Per nucleotide RMSF of the TS and NTS DNA in modeled sgRNA and dgRNA Cas9 ternary complexes with E1 (**A**), E2 (**B**), and E3 (**C**) spacer sequences.

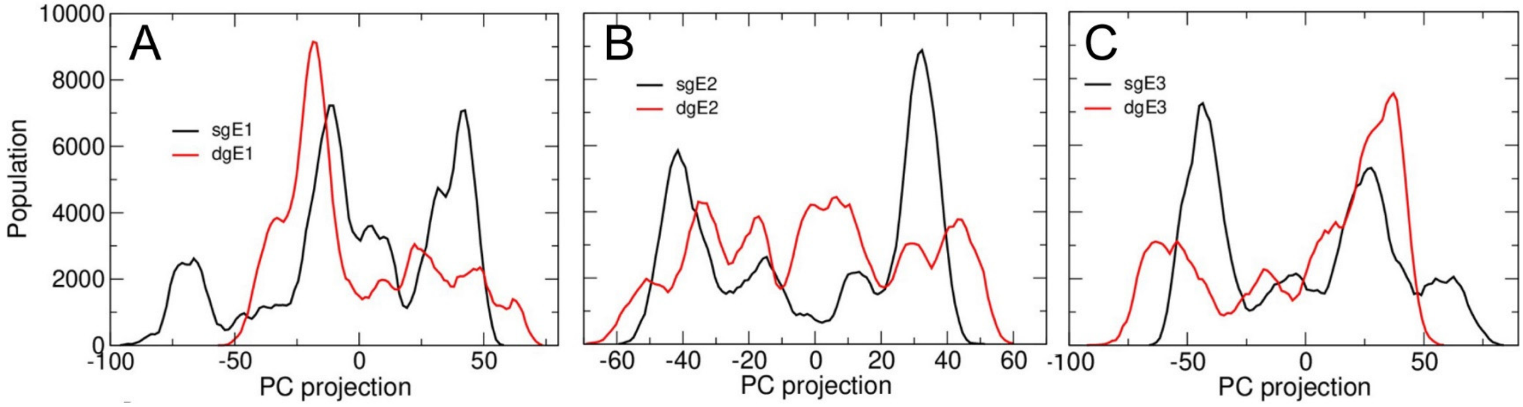

Figure S17. Histogram distribution showing the projection of PC1 of the protein backbone of (**A**) E1, (**B**) E2, and (**C**) E3 Cas9 sgRNA and dgRNA ternary complexes. All calculations have been performed using the 500 ns GaMD trajectories.

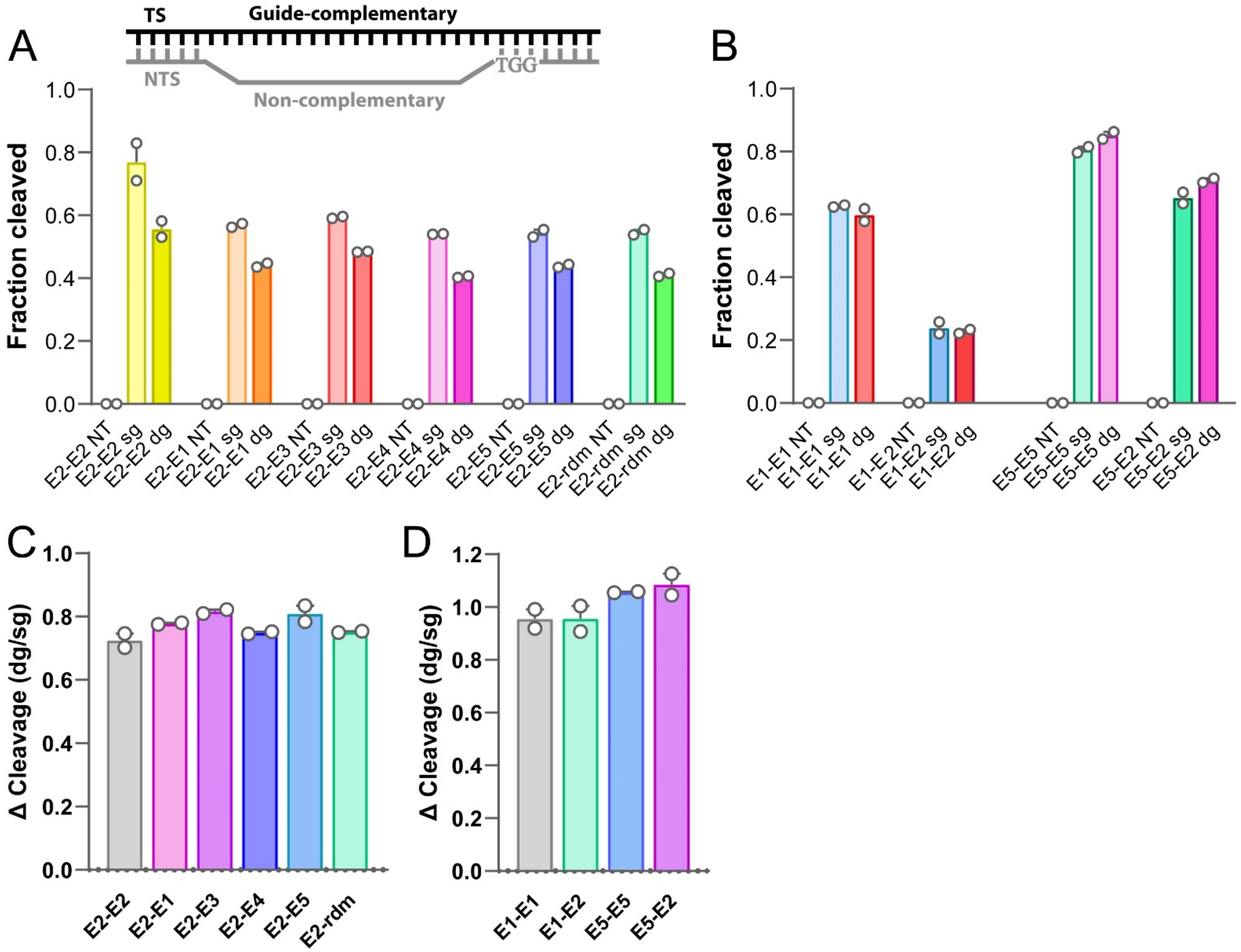

<u>Figure S18</u>. *In vitro* cleavage assays where a TS complementary to the E2 spacer sequence (**A**) or E1 and E5 spacer sequences (**B**) were held constant while the NTS was varied. For example, E2-E1 indicates a dsDNA with an E2 TS and an E1 NTS. E2-rdm indicates a randomized NTS sequence. n=2 replicates. (**C-D**) Quantification of data shown in panels A and B demonstrating the average difference in *in vitro* cleavage by Cas9 guided by E2, E1, or E5 guides with target DNA substrates containing a matched TS but unmatched and non-complementary NTS sequences (n=2).

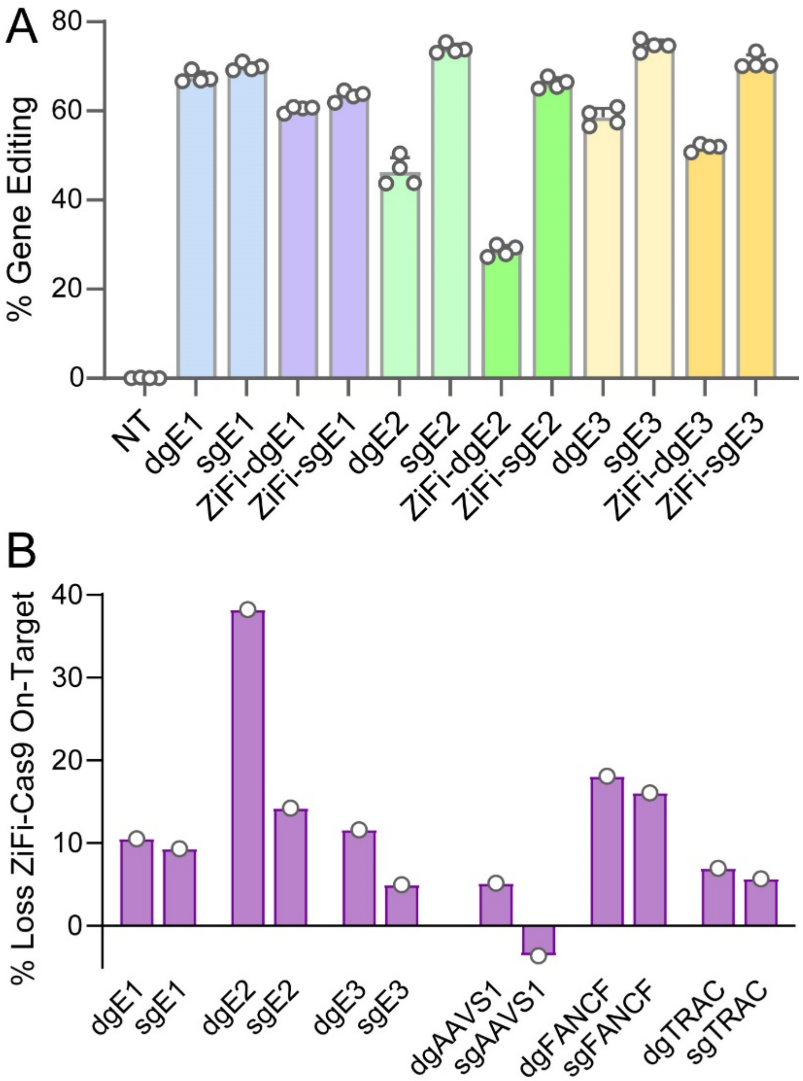

Figure S19. On-target and off-target editing with ZiFi-Cas9 and sgRNA and dgRNA. (**A**) Editing by E1, E2, and E3 dgRNAs or sgRNAs with normal or ZiFi-Cas9 quantified by EGFP knockout in HEK 293T cells and flow cytometry. Error is S.E.M. (**B**) Loss of average on-target editing for results shown in panel A and results shown in Figure 6A.

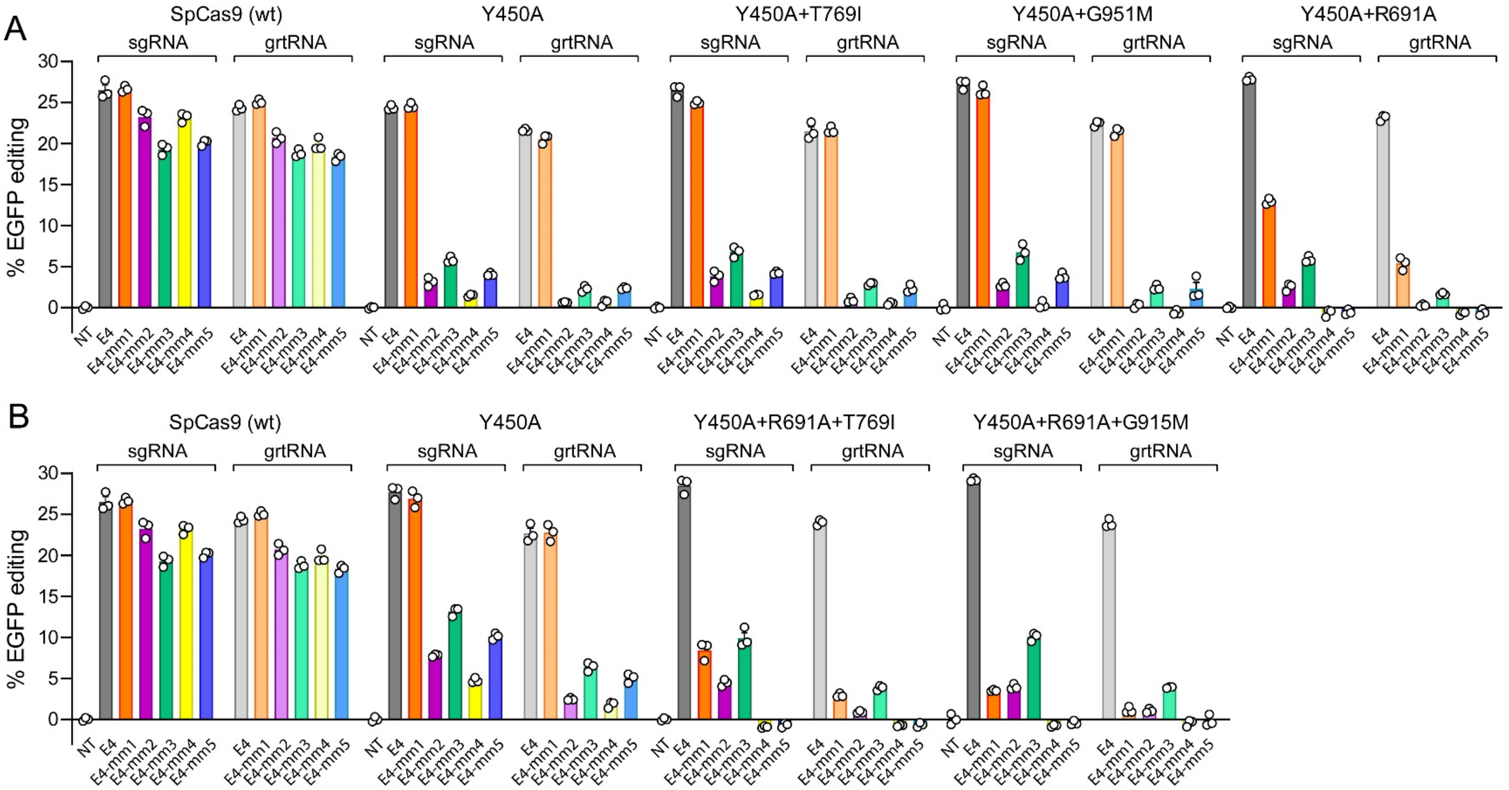

<u>Figure S20</u>. On and off target editing of EGFP with E4 guides and high-fidelity mutants of Cas9. (**A-B**) Screening of specific ZiFi mutations to identify those that contribute the greatest to ZiFi-Y450A performance using EGFP editing and E4 guides (n=3). Error bars are S.E.M.

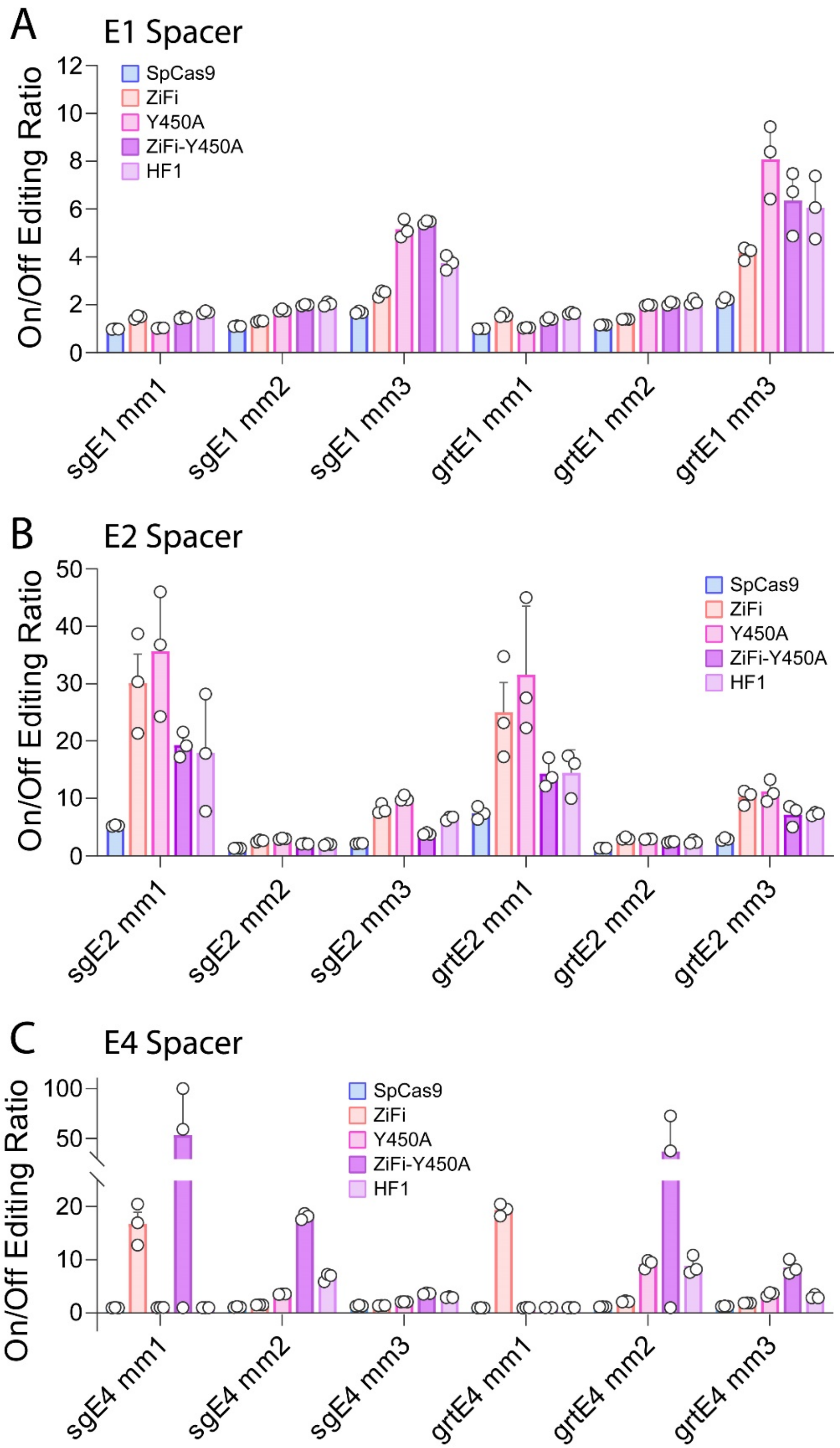

<u>Figure S21</u>. On/off target editing ratios for Cas9 variants using sgRNA and grtRNA. (**A-C**) On/off target editing ratios were calculated for E1, E2, and E4 spacers for sgRNA and grtRNA and various high-fidelity Cas9 mutants using results presented in Figure 8 (n=3). Error is reported as S.E.M.

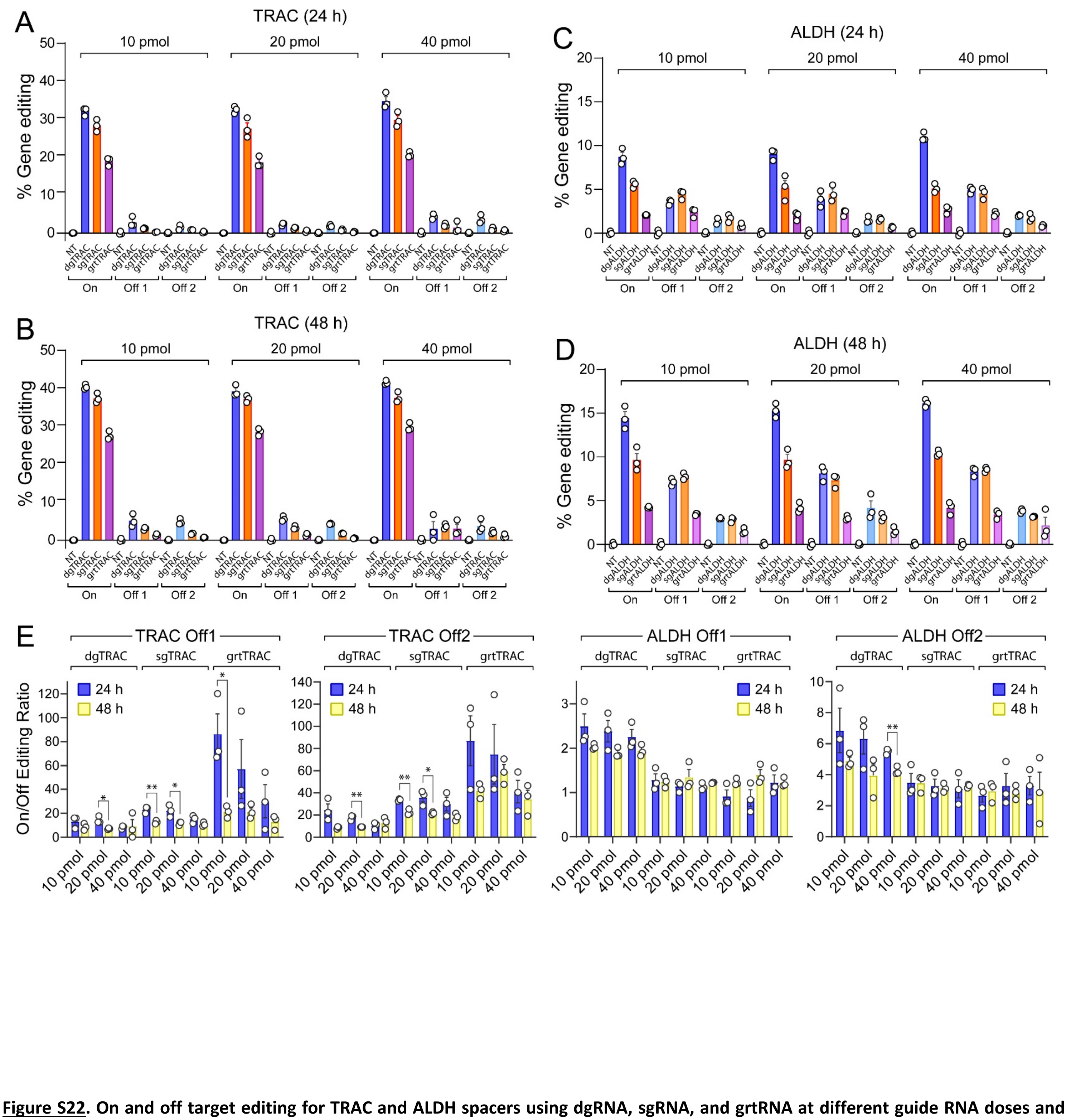

Figure S22. On and off target editing for TRAC and ALDH spacers using dgRNA, sgRNA, and grtRNA at different guide RNA doses and time points. HEK 293T cells were transfected with 10, 20, and 40 pmols of TRAC-targeting guide RNA and gDNA harvested at (**A**) 24 or (**B**) 48 h post-transfection. The same method was used to target the ALDH gene for (**C**) 24 h or (**D**) 48 h. Editing was quantified by nanopore sequencing and CRISPResso2. n=3 replicates. Error is reported as S.E.M. (**E**) On/off target editing ratios were calculated from data presented in panels A-D. Only statistically significant p-values are reported. Unreported p-values were n.s. Error is reported as S.E.M. Unpaired t-test p-value: n.s. = not significant, * > 0.05, ** > 0.01, *** > 0.005, **** > 0.0001.
